# Frequent copy number gains of *SLC2A3* and *ETV1* in testicular embryonal carcinomas

**DOI:** 10.1530/ERC-20-0064

**Published:** 2020-06-10

**Authors:** Andreas M Hoff, Sigrid M Kraggerud, Sharmini Alagaratnam, Kaja C G Berg, Bjarne Johannessen, Maren Høland, Gro Nilsen, Ole C Lingjærde, Peter W Andrews, Ragnhild A Lothe, Rolf I Skotheim

**Affiliations:** 1 Department of Molecular Oncology, Institute for Cancer Research, The Norwegian Radium Hospital, Oslo University Hospital, Oslo, Norway; 2 Institute for Clinical Medicine, Faculty of Medicine, University of Oslo, Oslo, Norway; 3 Department of Informatics, Faculty of Mathematics and Natural Sciences, University of Oslo, Oslo, Norway; 4 The Centre for Stem Cell Biology, Department of Biomedical Science, The University of Sheffield, Sheffield, UK

**Keywords:** testicular germ cell tumour, DNA copy number, 12p, SLC2A3, ETV1, embryonal carcinoma

## Abstract

Testicular germ cell tumours (TGCTs) appear as different histological subtypes or mixtures of these. They show similar, multiple DNA copy number changes, where gain of 12p is pathognomonic. However, few high-resolution analyses have been performed and focal DNA copy number changes with corresponding candidate target genes remain poorly described for individual subtypes. We present the first high-resolution DNA copy number aberration (CNA) analysis on the subtype embryonal carcinomas (ECs), including 13 primary ECs and 5 EC cell lines. We identified recurrent gains and losses and allele-specific CNAs. Within these regions, we nominate 30 genes that may be of interest to the EC subtype. By *in silico* analysis of data from 150 TGCTs from The Cancer Genome Atlas (TCGA), we further investigated CNAs, RNA expression, somatic mutations and fusion transcripts of these genes. Among primary ECs, ploidy ranged between 2.3 and 5.0, and the most common aberrations were DNA copy number gains at chromosome (arm) 7, 8, 12p, and 17, losses at 4, 10, 11, and 18, replicating known TGCT genome characteristics. Gain of whole or parts of 12p was found in all samples, including a highly amplified 100 kbp segment at 12p13.31, containing *SLC2A3.* Gain at 7p21, encompassing *ETV1*, was the second most frequent aberration. In conclusion, we present novel CNAs and the genes located within these regions, where the copy number gain of *SLC2A3* and *ETV1* are of interest, and which copy number levels also correlate with expression in TGCTs.

## Introduction

Patients with testicular germ cell tumours (TGCTs) have good prognosis due to high sensitivity to platinum-based chemotherapy (Voutsadakis 2014). The Surveillance, Epidemiology, and End Results (SEER) program (2008–2014) reported a 5-year survival rate of 95.3% for patients with all tumour stages and 73.7% for those with distant metastasis (Noone* et al.* 2018). However, long-term side effects and morbidity after chemotherapy is a problem in this young patient group, and therefore, research is essential (Oldenburg* et al.* 2007, Kraggerud* et al.* 2009). Young Caucasian men have the highest TGCT incidence (Rosen* et al.* 2011) with an unexplained, marked increase over the last 50 years, especially in industrialized countries. Genome-wide association studies (GWAS) and large-scale meta-analyses (Chung* et al.* 2013, Rajpert-De Meyts* et al.* 2016, Litchfield* et al.* 2017, Wang* et al.* 2017) have identified above 40 susceptibility loci. The mutation load has been found to be low, comparable to paediatric cancer types (Brabrand* et al.* 2015, Litchfield* et al.* 2015), but TGCTs are characterized by aneuploidy and a high degree of DNA copy number changes (Oosterhuis* et al.* 1989, Lothe* et al.* 1995, Taylor-Weiner* et al.* 2016).

TGCTs can be divided into two main histological types, seminomas and non-seminomas, with the latter comprising embryonal carcinomas (ECs), teratomas, choriocarcinomas, and yolk sac tumours. The various histological subtypes of TGCT have remarkably similar DNA copy number aberration (CNA) patterns, although some particular differences have been described (Kraggerud* et al.* 2002, Skotheim* et al.* 2006, Korkola* et al.* 2008). The isochromosome 12p and/or gain of 12p sequences are pathognomonic to TGCT and used for diagnostic purposes for extragonadal tumours of unknown origin (Sandberg* et al.* 1996). Most genome-wide DNA copy number studies of TGCTs to date have been performed using relatively low-resolution technologies, but recently TCGA published a multilevel genomics paper, including next-generation sequencing and high-resolution single nucleotide polymorphisms (SNPs) microarray analysis of 150 TGCTs, including 27 tumours classified as EC (18 pure EC and 9 mixed) according to the International Classification of Diseases for Oncology (ICD-O) morphological codes (Shen* et al.* 2018).

EC is a pluripotent histological subtype of TGCT that can be present alone or as one of several components in the tumour. ECs can be considered the malignant counterpart of embryonic stem (ES) cells, as both are pluripotent and have the capacity to differentiate. Identification of molecular differences between the two cell types may help resolve tumourigenic mechanisms and cellular pathways involved. We previously identified a discriminating gene expression signature between EC and ES cell lines, including a number of pluripotency and cancer-related genes (Alagaratnam* et al.* 2013). ES cell lines have been characterized for DNA CNAs on high-resolution SNP platforms (Närvä* et al.* 2010, Amps* et al.* 2011), where several higher-passage cells showed aberrations similar to those in TGCTs (Baker* et al.* 2007).

In this study, we profiled 13 pure primary EC tumours, as well as 12 cell lines (5 EC and 7 ES) on the high-resolution, whole-genome Affymetrix SNP 6.0 DNA copy number platform. We present a comprehensive overview of the EC subtype, identifying recurring regions of loss of heterozygosity (LOH) and focal regions of gains and losses, which harbour genes that may be of importance in EC development. These genes were further investigated in publically available multi-omics datasets and a transcriptional impact was confirmed for several of the genes.

## Materials and methods

### Sample preparations

Genomic DNA from 13 primary ECs had previously been isolated by phenol/chloroform extraction. Tumour percentage was estimated by an experienced pathologist on the basis of haematoxylin and eosin stained sections for 10/13 samples. For each case, the tumour percentage was calculated as the average of the tumour percentage of three sections, taken at either end and in the middle of the tumour sample used for DNA isolation. The median pathology tumour percentage was 49%, and ranged from 22% to 78%. Ten of the 13 primary ECs included in the current study have previously been analysed by chromosomal comparative genomic hybridization (cCGH; *n* = 6, Kraggerud* et al.* 2002) and array CGH (aCGH; *n* = 5, Skotheim* et al.* 2006), with one sample (EC no. 1838) analysed with both technologies.

Genomic DNA was isolated from five EC cell lines (NTERA2, 2102Ep, 833KE, TERA1, and NCCIT) and seven early-passage (<50 passages) ES cell lines (Shef3, Shef4, Shef6, Shef7, H7, H9, and H14 using the AllPrep DNA/RNA Mini kit (Qiagen). All cell lines were cultured, sorted by the SSEA3-antigen, and fingerprinted by analysis of short tandem repeats as previously described (Alagaratnam* et al.* 2013).

### DNA copy number profiling of primary tumours and cell lines

Three sets of samples were analysed for genome-wide DNA copy number on Affymetrix SNP 6.0 microarrays: primary EC tumours (*n* = 13), EC cell lines (*n* = 5), and ES cell lines (*n* = 7). For each sample, 500 ng of genomic DNA was used as input for the Cytogenetics Copy Number Assay protocol for Genome-Wide Human SNP 6.0 arrays (Thermo Fisher Scientific). The samples were individually processed and hybridized as described in the Affymetrix Cytogenetics Copy Number Assay User Guide (P/N 702607 Rev. 2).

### Data processing, target region analysis and statistics

The resulting cell intensity (CEL) files after hybridization were within recommended QC thresholds (CQC >0.4; MAPD <0.35). Signal extraction and pre-processing of the raw data was performed using the PennCNV protocol modified for Affymetrix genotyping arrays with Affymetrix Power Tools version 1.15.0 as described earlier (Sveen* et al.* 2016). HapMap samples previously analysed on the SNP Array 6.0 (*n* = 270), were used as reference for normalization, log R ratio (LRR), and B-allele frequency (BAF) calculation. Probes targeting the allosomes, control probes (*n* = 3643), duplicate probes (one of the two probes covering overlapping genomic loci (*n* = 187), and probes mapping to regions with recurrent high frequency aberrations in non-cancer samples from several organs (*n* = 6668) were removed (Sveen* et al.* 2016).

For copy number analysis, preprocessed LRR data results from primary tumours and cell lines were used for single-sample segmentation, using the Piecewise Constant Fitting (PCF) algorithm in the R package copynumber (version 1.14.0). The user-defined penalty parameter γ was set to 100 and the minimum number of probes per segment, k_min_ was set to 5. PCF segments with copy number estimates ≥0.15 were called as gains and segments with estimates ≤−0.15 were called as losses. The results were visualized using the copynumber R package. In addition, CNAs (gains, amplifications (defined as high gains >0.45), and deletions) were extracted for 27 target genes, earlier identified by our group as differentially expressed in EC vs ES (Alagaratnam* et al.* 2013).

For genomic identification of significant cancer related regions/genes, PCF segmented data for the primary ECs was used as input for the GISTIC 2.0.22 algorithm (Mermel* et al.* 2011). Copy number estimates >0.1 were called as copy number gain, while estimates <−0.1 were called as loss. The broad length cut-off was set to 0.5 (−brlen 0.5), the confidence level was set to 0.90 (−conf 0.90), normal arbitrated peel-off was performed (−armpeel 0), and we calculated the significance of deletions at a gene level (−genegistic 1), otherwise default settings. The reference genome file hg19.mat was used. Significant broad events were defined as events with a q-value <0.05, and significant focal events as events with q-values <0.25.

Preprocessed and normalized LRR and BAF data for the primary ECs was analysed using the allele-specific copy number analysis of tumours (ASCAT) v.2.3 algorithm to obtain allele-specific copy number estimates (Van Loo* et al.* 2010). ASCAT data were subsequently used to call regions with amplifications and LOH. However, as blood/germline DNA was not analysed, the LOH regions may include germline homozygous regions. By ASCAT, we also estimated ploidy and aberrant cell fraction of each tumour. The penalty parameter was set to 50 and discrete copy number states were determined relative to the median genome-wide copy number in each tumour sample.

The fraction of the genome with CNA or LOH was calculated as the number of aberrant bases out of the total number of bases with copy number and LOH estimate available, respectively.

Copy number estimates per gene were retrieved by mapping chromosomal segments from each sample to the R implemented transcript database TxDb.Hsapiens.UCSC.hg19.knownGene (v3.2.2) (Carlson *et al.* 2015), utilizing the findOverlaps function from the GenomicRanges R package (v1.28.3) (Lawrence* et al.* 2013). Gene symbols were collected using the R package org.Hs.eg.db (Carlson 2017) and updated to the approved symbols according to HUGO Gene Nomenclature Committee. For GISTIC, the output contains the genes located in the identified focal regions. However, to obtain a final target gene list, the regions identified with focal CNAs by GISTIC, were also manually examined for protein coding genes in Ensembl (Version 87, GRCh37) and these were added to the list of target genes. All genomic positions refer to genome version GRCh37 (hg19). Pseudogenes and genes annotated as non-coding in Ensembl were not considered.

Analysis of DNA and RNA level data from TCGA are described in the Supplementary Materials and methods (see section on supplementary materials given at the end of this article).

## Results

### DNA copy number aberrations in primary ECs, compared to EC and ES cell lines

**Figure 1:**
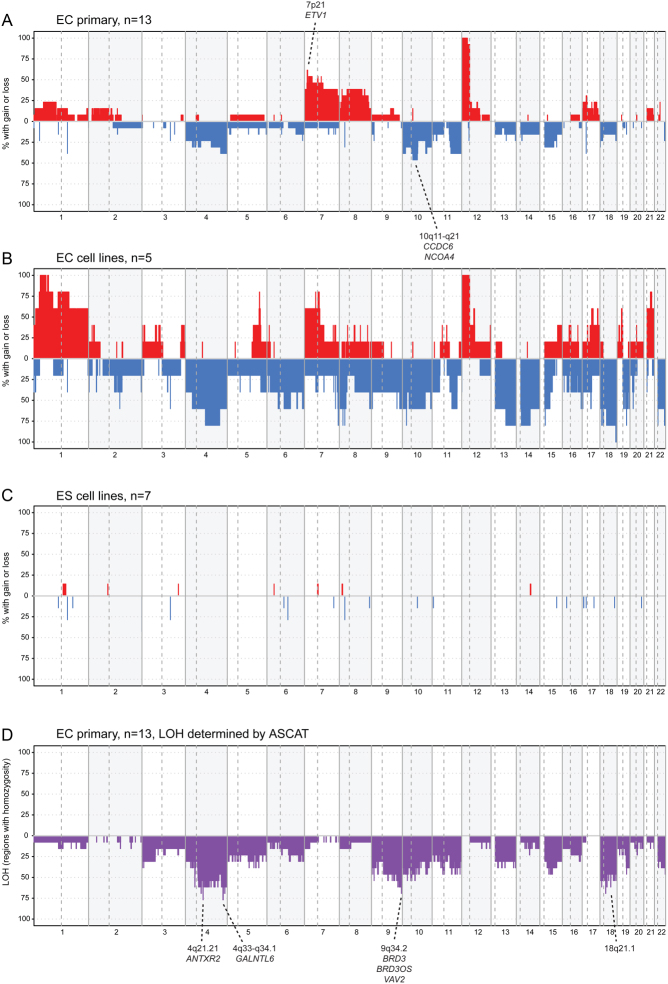
DNA copy number aberration frequency. Frequency plots showing gain (red) and loss (blue) identified from PCF segments, and data are plotted along chromosomes 1 to 22: (A) primary ECs (*n* = 13) with the two most frequent CNAs apart from the ubiquitous 12p amplification indicated; (B) EC cell lines (*n* = 5); and (C) ES cell lines (*n* = 7). In (D) frequencies of LOH among the primary ECs and the genes located in the regions with highest frequency are shown.

By use of PCF segmentation, we identified similar CNAs in primary ECs and EC cell lines (Fig. 1). In general, the frequencies of CNAs were higher for EC cell lines than for primary EC tumours. The most frequent aberrations observed for primary ECs were gain of 12p (100% of the samples) and gains of the whole or parts of chromosomes 7, 8, and 17 and losses of the whole or parts of chromosomes 4, 10, 11, 15, and 18 (>30%). From the PCF segmented data, apart from gain of 12p, the two most frequent aberrations were a region of gain at 7p21 (12,327,848–14,412,764) and a region of loss at 10q11-q21 (47,757,274–68,156,269). The 7p21 region, gained in 8/13 (>60%) ECs, contains only five genes (*ARL4A*, *DGKB*, *ETV1*, *SCIN*, and* VWDE*), whereas the region at 10q11-q21, lost in 6/13 (>45%) ECs, contains 81 genes (Table 1). Among the affected genes, *ETV1*, *CCDC6*, and *NCOA4* are causally implicated in cancer according to the Cancer Gene Census (Tate *et al.* 2019).

**Table 1:** Focal aberration events identified in primary ECs.

CNA type	Analysis	Cytoband	Start (bp)	End (bp)	Segment size (bp)	Genes in region
Gain	PCF	7p21	12,327,848	14,412,764	2,084,916	*ARL4A*, *DGKB*, *ETV1*, *SCIN*, *VWDE* (*ETV1* in COSMIC cancer gene census)
Gain	GISTIC	12p13.31	7,974,004	8,135,091	161,087	*SLC2A3*, *SLC2A14*
Gain	GISTIC	12p11.1	34,275,407	37,857,943	3,582,536	*-*
Gain	GISTIC	22q11.23	25,682,781	25,910,952	228,171	*LRP5L*
Loss	GISTIC	1p36.11	25,571,270	25,673,153	101,883	*RSRP1*^a^, *RHD*, *TMEM50A*^a^
Loss	GISTIC	1q21.3	152,747,127	152,773,904	26,777	*LCE1D*, *LCE1E*^b^, *LCE1F*
Loss	GISTIC	3q22.1	129,695,715	129,819,637	123,922	*ALG1L2*^b^, *TRH*^a^
Loss	PCF	10q11-21	47,757,274	68,156,269	20,398,995	81 genes, including COSMIC cancer genes: *CCDC6* and *NCOA4*
Loss	GISTIC	11q11	55,363,341	55,541,283	177,942	*OR4C6*, *OR4C11*, *OR4P4*, *OR4S2*
Loss	GISTIC	17p11.2	18,397,113	18,441,307	44,194	*LGALS9C*^a^

Regions of gain and loss significant by GISTIC analysis (FDR *q* values <0.25) and the most frequent non-significant focal region of gain and loss identified by PCF segmentation are listed. Genes located in the corresponding region are presented to the right.

^a^identified from Ensembl; ^b^Identified also in ES cell lines.

CNA, copy number aberration.

From the individual tumour CNA plots (Supplementary Fig. 1), we observed that the 13 primary ECs varied markedly in both number of gains and losses and the proportion of the genome affected by CNAs. The aberrations were typically broad events of chromosome arm-length, and median genome wide CNA for the 13 EC samples was 12% (mean 23%; Supplementary Fig. 1). Three samples (EC 28, EC 1740, and EC 1838) had only nine percent genome wide CNA, whereas the two samples with the highest percent of aberrations (EC 1017 and EC 3113) had 53 and 56% genome wide CNA.

We observed five recurrent, CNA regions in the seven early passage ES cell lines (Fig. 1 and Table 2), including focal loss in regions 1q21.3 and 3q22.1 (both in two ES cell lines). These regions overlap with larger segments of loss also found in primary ECs (Table 1) and covers the genes *LCE1E* and *ALG1L2*, respectively.

**Table 2:** Recurrent regions of loss in ES cell lines, showing loss in EC primary/cell lines.

Cytoband	Cell line	Start (bp)	End (bp)	Segment size (bp)	Mean Log R ratio	Detected in EC cell lines	Detected in primary ECs
2q37.3	H7	242,915,466	243,034,686	119,220	−0.61		
	H14	242,915,466	243,089,456	173,990	−0.49	2/5	1/13
	Shef6	242,915,466	243,089,456	173,990	−0.49		
1q21.3	H7	152,759,678	152,768,700	9022	−1.46	2/5	5/13
	Shef6	152,759,678	152,768,700	9022	−1.38		
3q22.1	Shef6	129,766,586	129,806,236	39,650	−0.96	3/5	3/13
	Shef7	129,763,698	129,806,236	42,538	−0.70		
6q16.1	H7	95,452,264	95,533,338	81,074	−0.75	3/5	0/13
	H14	95,442,761	95,533,338	90,577	−0.80		
8p21.2	H7	24,974,476	24,984,333	9857	−1.12	4/5	4/13
	H14	24,974,522	24,984,333	9811	−1.34		

### Significant DNA copy number events in primary ECs

PCF-segmented data from the 13 primary ECs were analysed with GISTIC to identify statistically significant CNAs, both in terms of chromosome arm-level (broad; Supplementary Table 1) and focal events. We identified three significant focal regions of gain, located at 12p13.31, 12p11.1, and 22q11.23; and five significant focal regions of loss, located at 1p36.11, 1q21.3, 3q22.1, 11q11, and 17p11.2 (Table 1 and Supplementary Fig. 2). Although the 1q21.3 and 3q22.1 segments covers the *LCE1E* and *ALG1L2* genes, also found to be lost in ES cell lines, they were not excluded from further analyses.

### Ploidy, allele-specific copy number profiles, and LOH in primary ECs

Ploidy estimates for the 13 tumours, as calculated by the ASCAT algorithm, ranged from 2.3 to 5.0. The ploidy values formed two clusters, one between 2.3 and 2.8 (9/13 tumours) and one between 4.4 and 5.0 (4/13 tumours; Supplementary Fig. 3). Individual allele-specific profiles of the 13 tumours are shown in Supplementary Fig. 4.

**Figure 2:**
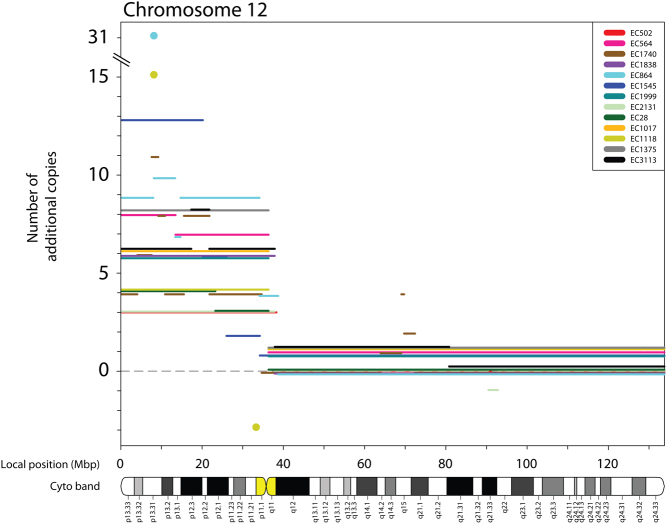
Minimal amplicon of 100 kbp on chromosome arm 12p. Copy number aberrations on chromosome 12 from 13 primary ECs, plotted by median adjusted copy number, from ASCAT analysis and genomic position. To allow visibility of all DNA copy number chromosome 12 segments, for each tumour, the lines were adjusted. Segments <0.5 Mb are enlarged as circles to increase their visibility.

The ASCAT analysis revealed a minimal amplicon of 100 kbp (chr12: 8,024,362–8,123,900) that was present at 15 and 31 additional copies in two individual tumours and gained across all 13 tumours (Fig. 2). For 12/13 ECs, this amplicon was the segment, or was included within the 12p segment, with the overall highest copy number. This segment contains the *SLC2A3* gene and parts of *SLC2A14*.

LOH was determined from the allele-specific copy number profiles for the primary ECs (Fig. 1). The fraction of the genome with LOH varied from 15 to 42%, with a median of 26%. LOH was detected in one or more samples for all the autosomal chromosomes, and encompassed larger regions for 6 of 13 ECs on chromosome arms 4q, 9q, 18p, and 18q. Within these broad regions of LOH, four additional focal regions of LOH were detected (4q21.21, 4q33-q34.1, 9q34.2, and 18q12.1), indicated as peaks in Fig. 1, and present in at least 9 of the 13 ECs (Table 3). Interestingly, a region on chromosome arm 9q showed frequent LOH but no copy number loss (Fig. 1), and is thus a copy neutral LOH.

**Table 3:** Allele-specific LOH identified in primary ECs.

Cytoband	Samples with LOH	Start (bp)	End (bp)	Segment size (bp)	Genes in region
4q21.21	10	80,576,187	81,079,422	503,235	*ANTXR2*
4q33-q34.1	10	171,243,266	172,739,515	1,496,249	*GALNTL6*
9q34.2	9	136,642,066	136,992,038	349,972	*BRD3*, *BRD3OS*, *VAV2*
18q12.1	9	25,987,834	26,905,128	917,294	-

Focal regions of LOH identified by ASCAT in primary ECs with corresponding genes located in the region.

### Differentially expressed genes associated with DNA copy number levels

**Figure 3:**
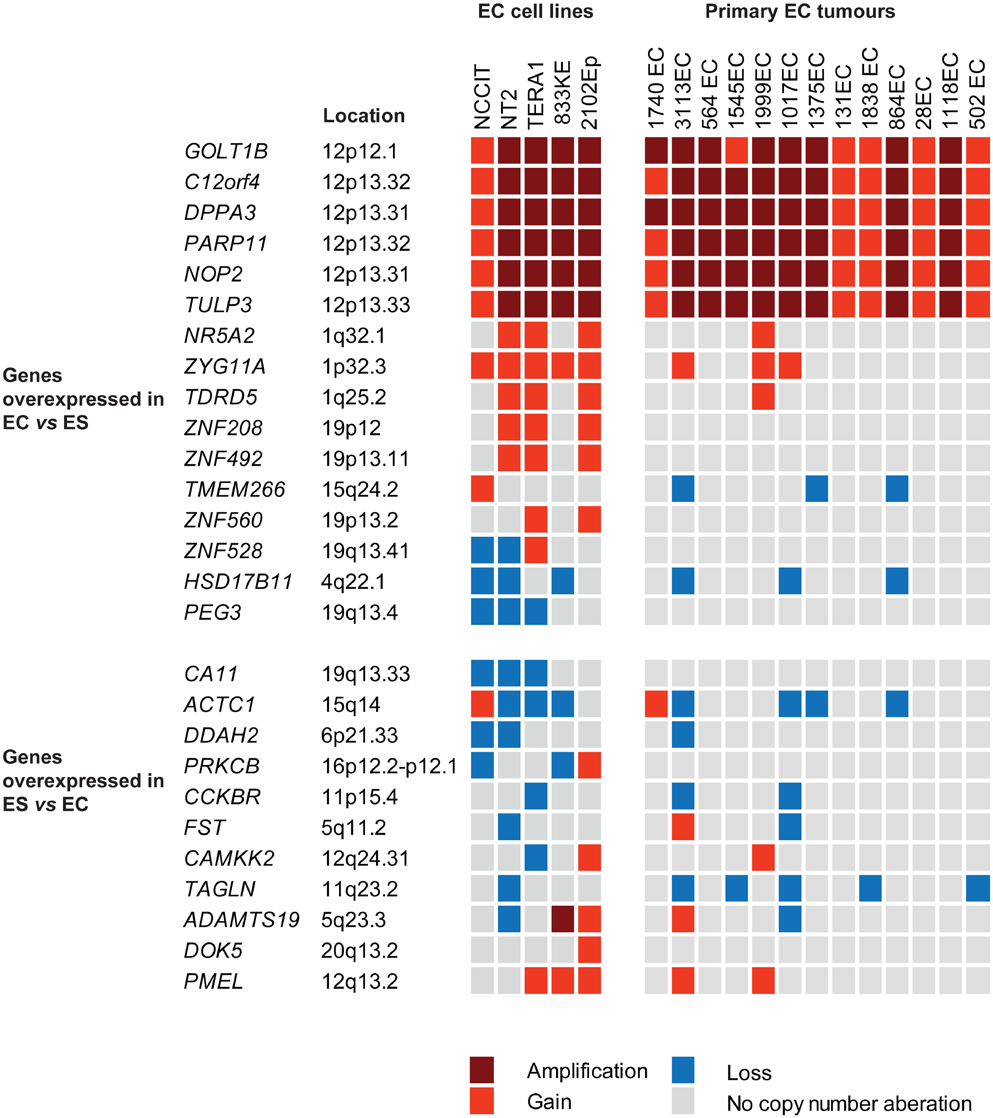
DNA copy number alteration and gene expression in EC. DNA copy number status for differentially expressed genes (*n* = 27) in EC cell lines versus ES cell lines, as identified in a previous study (Alagaratnam *et al*. 2013). The copy number status was determined by PCF for five EC cell lines and 13 primary ECs. Thresholds applied: loss <-0.15; gain>0.15; amplification >0.45.

In a previous study, we identified 28 differentially expressed genes between EC and ES cell lines (Alagaratnam* et al.* 2013). The relative gene expression and the corresponding copy number changes from PCF for 27 genes (one was located on chromosome X) are shown for EC cell lines and tumours in Fig. 3. Six of the 16 genes with higher expression in EC compared to ES cell lines are localized on chromosome arm 12p (*C12orf4*, *DPPA3*, *GOLT1B*, *NOP2*, *PARP11*, and* TULP3*) and showed gain in all and amplification in most EC cell lines (4/5) and primary ECs (9/13). However, the 10 remaining genes, and the 11 genes with lower expression in EC compared to ES cell lines, were in regions with few CNAs.

### Identification of target genes affected by CNAs

Within the identified regions of CNA or LOH in the EC subtype, there are several protein-coding genes of potential interest to EC development. There are 16 genes located in the GISTIC-defined focal loss or gain regions, five genes within the ASCAT-defined LOH regions and in the two regions showing the most frequent aberrations (apart from 12p), as identified by the PCF segmented data, there are three genes known to be cancer critical genes according to COSMIC. In addition, six genes previously identified as differentially expressed between EC and ES cell lines were also found to be gained or amplified in EC tumours. Taken together, we nominate 30 protein-coding genes affected by CNAs and/or LOH to be of interest to the EC subtype (Supplementary Table 2).

### DNA copy number and mRNA expression among TGCTs in TCGA data

For further investigation of the genes affected by CNAs and/or LOH in ECs, we analysed copy number levels for 27 of the 150 TGCT tumours from TCGA classified as EC according to the ICD-O morphological codes (18 pure EC and 9 mixed). The genes identified at 12p, including *SLC2A3* and *SLC2A14*, were gained in all 27 samples and were highly amplified in 8 of the samples (30%; Supplementary Fig. 5). *ETV1*, located at 7p21 was gained in 25 of the 27 samples, while *CCDC6* and *NCOA4* located at 10q11-q21 had copy number loss in 21 and 20 samples, respectively. Surprisingly, many of the genes located in focal regions identified as statistically significant loss in our cohort by GISTIC, for example, 1q21.3, 1p36.11 and 3q22.1, were infrequently lost in the EC cohort from TCGA (Supplementary Fig. 5).

A significant correlation (q < 0.05) between DNA copy number and mRNA expression data was seen for 15 of the 30 genes. These were *ETV1* and *CCDC6* (from PCF-identified gain/loss); *LRP5L* and* SLC2A3* (from GISTIC-identified focal gain); *TMEM50A* and *TRH* (from GISTIC-identified focal loss); *ANTXR2*, *BRD3*, *BRD3OS*, and *VAV2* (from ASCAT-identified LOH); *C12orf4*, *DPPA3*, *GOLT1B*, *NOP2* and* PARP11* (previously identified as differentially expressed between EC and ES cell lines; Supplementary Fig. 5). Correlation between copy number and gene expression remained significant for four of the genes when only considering the EC subset (*n* = 27). This included a strong correlation for *ETV1* (R = 0.8, q < 0.0001).

### Somatic mutations among TGCTs in TCGA

TCGA whole-exome sequencing data were examined for somatic mutations in the 30 genes. We found that six of the 150 TGCT samples contained markedly higher numbers of mutations genome-wide (median 1091.5 mutations), than the remaining TGCTs (median 38.5 mutations), and omitted these from further analysis. Among the included 144 tumour samples, 20 (4/20 diagnosed as EC) were found to harbour somatic, non-synonymous mutations in 11 of the 30 genes (Supplementary Table 3). Non-synonymous mutations in two or more TGCTs were identified in* ANTXR2*, *LCE1F*, *SLC2A3*, *SLC2A14*, and* TULP3.*

### Fusion transcript breakpoints including target genes/regions among TGCTs in TCGA

Next, we evaluated whether CNAs were associated with generation of fusion genes. After analysis of RNA-sequencing data from TCGA’s TGCT samples, the intersection of the outputs from two fusion finder software, FusionCatcher and deFuse, resulted in 1956 nominated fusion transcript breakpoints (range 2 to 49 per sample, median = 10). None of these transcript breakpoints involved the 30 genes affected by CNAs. However, when considering breakpoints of fusion transcripts within 1 Mbp of the identified CNA segments, we detected the previously described *CLEC6A-CLEC4D* read-through fusion transcripts (Hoff* et al.* 2016) in 12 of 150 TGCTs. Additionally, two fusion transcripts, *LIN28A-CD52* and *LRP6-LRRC23* were each detected in individual samples. Both these fusion transcripts were nominated with breakpoints joining the canonical exon-boundaries of the partner genes and are predicted to maintain reading frames (Table 4). These two fusion transcripts were however found to be predominantly expressed in the seminoma subtype of the TCGA samples (Table 4).

**Table 4:** Fusion transcripts detected in TGCTs.

Gene A	Gene B	Sample	ICD-O (histology)	CytobandA (gene A)	CytobandB (gene B)	Start–End (gene A)	Start–End (gene B)	Strand	Split reads	Spanning reads	Score^a^
*CLEC6A*	*CLEC4D*	TCGA-2G-AAEX-01	9061/3 (seminoma)	12p13.31	12p13.31	8608522–8630926	8662071–8674962	++	2	4	0.92
		TCGA-2G-AALN-01	NA	12p13.31	12p13.31	8608522–8630926	8662071–8674962	++	4	5	0.89
		TCGA-S6-A8JX-01	9061/3 (seminoma)	12p13.31	12p13.31	8608522–8630926	8662071–8674962	++	2	4	0.98
		TCGA-WZ-A7V3-01	9061/3 (seminoma)	12p13.31	12p13.31	8608522–8630926	8662071–8674962	++	8	5	0.96
		TCGA-XE-AAOF-01	9061/3 (seminoma)	12p13.31	12p13.31	8608522–8630926	8662071–8674962	++	2	4	0.98
		TCGA-YU-A912-01	9061/3 (seminoma)	12p13.31	12p13.31	8608522–8630926	8662071–8674962	++	5	12	0.94
		TCGA-ZM-AA0H-01	9061/3 (seminoma)	12p13.31	12p13.31	8608522–8630926	8662071–8674962	++	3	3	0.98
		TCGA-2G-AAFE-01	9061/3 (seminoma)	12p13.31	12p13.31	8608522–8630926	8662071–8674962	++	10	6	0.95
		TCGA-2G-AAFV-01	9071/3 (Yolk sac)	12p13.31	12p13.31	8608522–8630926	8662071–8674962	++	3	3	0.89
		TCGA-2G-AAHC-01	9061/3 (seminoma)	12p13.31	12p13.31	8608522–8630926	8662071–8674962	++	1	3	0.78
*LIN28A*	*CD52*	TCGA-2G-AAFV-01	9071/3 (Yolk sac)	1p36.11	1p36.11	26737269–26756213	26644448–26647014	-+	17	19	0.99
*LRP6*	*LRRC23*	TCGA-S6-A8JY-01	9061/3 (seminoma)	12p13.2	12p13.31	12268959–12419946	6982733–7023407	++	46	35	0.96

From analysis of TCGA’s TGCTs RNA sequencing data, we identified fusion transcripts with breakpoints mapping to within a 1 Mbp buffered regions of the CNA segments identified.

^a^Defuse probability score.

Interestingly, FusionCatcher and deFuse individually nominated a vast number of breakpoints involving *SLC2A3*; 137 and 364, respectively. These breakpoints did not include the same partner genes in the individual samples and were therefore not considered in the intersected analysis. However, we observed that the number of breakpoints nominated per sample correlated between FusionCatcher and deFuse and that the nominated breakpoints were mostly in ECs and mixed germ cell tumours (16 and 15 out of in total 43, respectively; Supplementary Fig. 6). Overall in 150 TGCTs, the correlation between gene expression and DNA copy numbers of *SLC2A3* was significant (Spearman: R = 0.55, q = 9 × 10^−12^), whereas when only considering cases that had at least one nominated fusion breakpoint with *SLC2A3* (*n* = 43), the correlation was not significant (Spearman: *R* = 0.26, *P* = 0.09).

## Discussion

We have here performed high-resolution DNA copy number analysis of the EC subtype of TGCT, and identified broad and focal CNAs as well as allele-specific CNAs, including LOH. We have nominated altogether 30 genes which may be related to EC within the regions affected by CNAs, including *SLC2A3* from chromosome arm 12p and *ETV1* on 7p21.

The CNA profiles varied in complexity among primary ECs. Both individual EC copy number profiles and the summarized overall CNA frequency plots, are in agreement with TGCT and EC profiles in particular (Kraggerud* et al.* 2002, Skotheim* et al.* 2006, Korkola* et al.* 2008), however, in this study with higher resolution than previously reported. Previous studies of the copy number landscape of EC include two aCGH studies (*n* = 25 (Korkola* et al.* 2008) and *n* = 32 (Gilbert* et al.* 2011)). Korkola *et al.* analysed several non-seminoma histological subtypes and did not find a prominent separation of subtypes on the basis of DNA copy number profiling, as opposed to the gene expression-based classification identified earlier (Korkola* et al.* 2005). However, Korkola *et al.* reported EC-specific genomic alterations at 1p33-31.2, 2p25.3, and 17p11.2-q21.32 (Korkola* et al.* 2008). Gilbert *et al.* profiled stage I ECs and identified novel minimal regions of overlap of gain at 6p21.33, 10q11.21, and 22q13.32, and of loss at 22q12.2. Our results are in agreement with alterations reported in these studies. However, apart from the common 12p gain and frequent 7p gain, none of the significant, focal CNA identified here were reported by Korkola* et al.* (2008) or Gilbert* et al.* (2011). To our knowledge, only one SNP microarray study has been published, including 18 pure ECs and 9 mixed TGCTs with a dominant EC proportion of in total 137 TGCTs (Shen* et al.* 2018). EC subtype-specific CNAs are not reported in this TCGA study; however, they report that ECs’ CNA profiles cluster into three of five identified CNA groups. Among the focal, GISTIC-identified alterations in the TGCT cohort of TCGA, gain at 12p12 is in agreement with our results.

### ES and EC cell lines

ES and EC cells have many common characteristics, and culture-adaptation of ES cells have been acknowledged as a model system for EC carcinogenesis (Andrews *et al.* 2005). All seven ES cell lines included are previously analysed for CNAs on SNP microarrays (Närvä* et al.* 2010, Amps* et al.* 2011). Aberrations, identified in individual cell lines at early passage were also found in our dataset, including gain at 2p11.2 and 3q26.1. A recurrent gain on 20q11.21 in ES cell lines is suggested to confer a growth advantage (Amps* et al.* 2011). However, this gain is not found in the ES cell lines applied in our study. Also, none of the primary ECs showed gain of the 20q11.21 region. Still, among EC cell lines, two showed gain and one a borderline gain, supporting that this CNA may be induced by cell culturing rather than relevant for EC tumourigenesis.

### Ploidy estimates of ECs

Ploidy estimates by ASCAT showed that 9/13 (69%) of primary ECs were hyperdiploid to triploid, while 4/13 (31%) were tetraploid to pentaploid. However, the algorithm gives an estimate of the on average ploidy and does not account for sub-clonality. This result is largely in agreement with previous studies, where ECs are often categorized as aneuploid or hypotriploid, and with flow cytometry often several aneuploid cell population are observed (Fosså* et al.* 1991, Burger* et al.* 1994). The near triploidy among ECs has also been shown in cytogenetic studies (Sandberg* et al.* 1996).

### High level amplification of the glucose transporters *SLC2A3* and *SLC2A14* in ECs

Gain of 12p was detected in all primary EC samples and EC cell lines, supporting its role as an early driver event in EC development. High-level amplification of 12p segments has been reported in TGCT (Kraggerud* et al.* 2002, Skotheim* et al.* 2006), mostly focusing on a 12p11.2-p12.1 amplicon (Bourdon* et al.* 2002, Zafarana* et al.* 2003). Interestingly, we identified two novel segments with focal amplification; a 3.5 Mbp segment on 12p11.1 with no annotated genes, and a 100 kbp segment on 12p13.31. The latter segment corresponds to minimal amplicons present at estimated 15 and 31 additional copies in two individual ECs. This segment overlaps with both a larger region of amplification at 12p13 identified in a CGH study of TGCT cell lines (Henegariu* et al.* 2004) and a 200 kbp region/gene cluster at 12p13.31 that exhibits coordinated over-expression in both ECs and seminomas (Korkola* et al.* 2006). The small, 100 kbp amplified region contains two glucose transporter genes, *SLC2A3* and parts of *SLC2A14*.

Increased *SLC2A3* expression is reported in TGCTs compared to normal testis (Rodriguez* et al.* 2003), and validated as a sensitive and specific marker for the EC and yolk sac tumour histological subtypes (Howitt* et al.* 2013). *In vitro* differentiation of EC cells, with subsequent loss of tumourigenic potential, is reported to repress several pluripotency genes at this locus, including *NANOG*, *GDF3*, and *DPPA3*, but also *SLC2A3* (Giuliano* et al.* 2005). *SLC2A14* is a paralog of *SLC2A3* and with major expression in testis. We showed in data from TCGA, that the expression significantly correlates with copy number gains for *SLC2A3*, but not for *SLC2A14*. These results imply that amplification and over-expression of *SLC2A3* may be a common mechanism for activation. *SLC2A3* and *SLC2A14* were among the most frequently mutated of the investigated target genes (each observed with somatic mutation in three TCGA TGCTs, where one had an EC component). A large number of fusion transcript breakpoints were nominated for *SLC2A3.* Interestingly, expression of *SLC2A3* and DNA copy number did not correlate significantly for the samples that had nominated *SLC2A3* fusion breakpoints, which indicates that overexpression of *SLC2A3* in these cases is regulated by other mechanisms than the number of gene copies alone.

The roles of *SLC2A14* and *SLC2A3* in cancer have more recently gained attention. *SLC2A14* (or *GLUT14*) expression is deregulated in several cancer types and is suggested to be a prognostic factor for a number of cancers, for example, in thyroid carcinoma (Chai* et al.* 2017). *SLC2A3* (alias *GLUT3*) encodes a glucose transporter with a five-fold higher affinity for glucose than its ubiquitous family member GLUT1 (Simpson* et al.* 2008), making its expression an advantage in glucose-poor microenvironments with high glucose demands, such as in certain tumour environments. Indeed, *SLC2A3* expression correlates with poor survival in several cancers, including brain and gastric cancers (Flavahan* et al.* 2013, Schlößer* et al.* 2017). While broad level gain of 12p in TGCTs appears likely to confer the pluripotent phenotype for initiation of tumourigenesis, the focal amplification of the region containing *SLC2A3* may grant a proliferative advantage in progression and development of the tumour.

### CNAs at 7p and 10q affect the cancer critical genes *ETV1* and *CCDC6*

The second most frequently gained (after 12p) and the most frequently lost regions in ECs were located at 7p and at 10q, respectively. Among the genes located in these regions, *ETV1*, *CCDC6*, and *NCOA4* are known cancer critical genes. Several previous studies indicate that the functions of these genes are relevant in respect to TGCT development. Activated KIT is reported to prolong ETV1 protein stability and cooperate with ETV1 to promote tumorigenesis in gastrointestinal tumours (Chi* et al.* 2010). Disruption of the KIT–KITLG/MAPK signalling pathway is implicated in TGCT formation both as a predisposing germline risk factor and somatic driver event (Litchfield* et al.* 2015, 2017). ETV1 has been shown to upregulate the expression of androgen receptor target genes and promote autonomous testosterone production (Baena* et al.* 2013).

CCDC6 is a tumour-suppressor and a pro-apoptotic protein involved in DNA damage response and repair (Merolla* et al.* 2012). Loss of CCDC6 has been suggested to contribute to testicular neoplastic growth (Staibano* et al.* 2013) and could enhance tumour progression by impairing apoptosis following DNA damage (Cerrato* et al.* 2018). In effect, loss of CCDC6 has also been implicated as a biomarker to sensitizing cancer cells to treatment with PARP inhibitors (Cerrato* et al.* 2018).

### Fusion genes located on chromosome arm 12p

We have previously identified novel fusion transcripts in TGCT (Hoff* et al.* 2016). In this study we analysed RNA sequencing data of TGCTs from the TCGA for the expression of fusion transcripts in proximity (1 Mbp) of identified regions of gain, loss, and LOH. We reasoned that CNAs may reflect structural rearrangements that form fusion genes. We repeatedly identified the fusion event *CLEC6A-CLEC4D* (*n* = 12 patients) and also two private fusion events, *LIN28A-CD52* and *LRP6-LRRC23.* These fusions were, however, found expressed in non-EC histological subtypes (Table 4). Both genes involved in the *CLEC6A*-*CLEC4D* and the *LRP6-LRRC23* fusion genes are located on chromosome arm 12p. Previously, we described several other private fusion genes on 12p (Hoff* et al.* 2016). The recurrent structural alterations of 12p may be a common mechanism for the generation and expression of fusion genes in TGCT. However, the biological impact of these mostly private fusion gene events is uncertain.

In conclusion, by use of high-resolution SNP microarrays and advanced analyses, we present allele-specific copy number profiles for primary ECs and several novel focal CNAs. Within the regions affected by CNAs, we report 30 target genes that may be of interest to further our understanding of the EC subtype. High amplification of a 100 kbp segment at 12p13.31 containing *SLC2A3* was identified and the second most common CNA identified as gain at 7p21 encompassed the cancer critical gene *ETV1*. Increasing DNA copy numbers were found to be correlated with increased gene expression of *SLC2A3* and *ETV1.*

### Supplementary Material

Supplementary Materials and methods. Description of the analysis of DNA and RNA level data from TCGA with corresponding references.

Supplementary Figure 1. DNA copy number aberrations in 13 primary ECs. Regions with gain (log R ratio >0.15, red) and loss (log R ratio <-0.15, blue) after segmentation by PCF are plotted along the genome (horizontal axis: chromosome numbers). The ECs are grouped by tumour stage. *as determined by ASCAT algorithm

Supplementary Figure 2. Amplified and deleted regions as called by GISTIC. (A) Focal regions of gain and (B) Focal regions of loss as identified in 13 primary ECs plotted by FDR q-value along the genome. Genes located in the significant regions (FDR-adjusted q < 0.25) are shown; * genes identified from Ensembl.

Supplementary Figure 3. Ploidy and aberrant cell fraction. Estimated ploidy versus aberrant cell fraction, determined by ASCAT analysis, plotted for the 13 primary ECs.

Supplementary Figure 4. Individual allele-specific profiles of 13 primary ECs in order of increasing ploidy. Copy number aberrations (CNA) illustrated as regions of gain and loss along the genome (horizontal-axis) and absolute copy number (vertical-axis). The red and blue colours represent the two specific alleles. Absolute copy number is shown up to ten (10) copies. In five samples there were segments with more than ten copies, and these are illustrated by cumulatively plotted segments above 10.

: Supplementary Figure 5. CNAs of identified target genes in TCGA EC samples. CNA levels as determined by TCGA GISTIC analysis are shown for the identified target genes (n = 30) in 27 EC samples in the cohort from TCGA. The genes are clustered by the type of CNA affecting their respective genomic regions as identified in the in-house cohort.

Supplementary Figure 6. TCGA gene expression vs. linear copy number. For 30 genes in regions of recurrent CNAs, the log2 transformed RSEM gene expression values are plotted against the DNA copy number for each sample in the TCGA cohort (N = 150). Significant correlations (i.e. corrected q values <0.05) between gene expression and CNA are presented in red. The samples are coloured according to ICD-O morphological codes, as provided by TCGA.

Supplementary Figure 7. Fusion transcript breakpoints in SLC2A3 vs. copy number aberration (CNA) and gene expression among TGCTs. The log2 transformed SLC2A3 mRNA expression values are plotted against CNA per TGCT sample. The size and colour of each data point reflect the average number of fusion transcript breakpoints involving SLC2A3 as nominated by FusionCatcher and deFuse. The shape of each point corresponds to ICD-O morphological codes, as provided by TCGA.

Supplementary Table 1. Statistically significant broad aberrations identified in primary ECs

Supplementary Table 2. Genes identified in regions of recurrent CNAs in EC

Supplementary Table 3. Somatic mutations identified in target genes

Supplementary Data and materials. PCF segmented copy number data from the in-house analysed EC cell lines (n = 5), primary EC samples (n = 13) and ES cell lines (n = 7).

## Declaration of interest

The authors declare that there is no conflict of interest that could be perceived as prejudicing the impartiality of the research reported.

## Funding

The study was supported by grants from the Norwegian Cancer Society (PR-2006-0442 to R A L and PR-2007-0166 to R I S), the Research Council of Norway, and the South-Eastern Norway Regional Health Authority. The authors also acknowledge NorStore, Notur, and Services for Sensitive Data at the University of Oslo for secure storage and processing of computer files and high-performance computation (projects NS9013S and NS9013K).

## Ethics approval and consent to participate

The biobank is registered according to Norwegian legislation (no. 953; Biobank Registry of Norway) and the project has been approved by the Norwegian Committee for Medical and Health Research Ethics (S-05368 and S-07453b).

## Availability of data and materials

The PCF segmented copy number data for the in-house analysed samples can be found in Supplementary Data and materials.

## Author contribution statement

Study design: S A, S M K, A M H, O C L, P W A, R I S, R A L. Acquisition of data: S A, S M K, A M H, B J, K C G B, M H. Analyses and/or interpretation of data: all authors. Writing of the manuscript: A M H, S M K and S A drafted the manuscript and all authors were involved in revision and have read and approved the final version. Study supervision: R I S and R A L.
