# Supplementary material for: Frequent copy number gains of SLC2A3 and ETV1 in testicular embryonal carcinomas
Source: Endocr Relat Cancer. 2020 Jun 10;27(9):457–68. doi: 10.1530/ERC-20-0064 (PMC7424350; doi:10.1530/ERC-20-0064)
Supplement: Supplementary Materials and methods. Description of the analysis of DNA and RNA level data from TCGA with corresponding references. [file supplementary_material.pdf]

Additional file 1.docx: Description of the analysis of DNA and RNA level data from TCGA with corresponding references

Analyses of DNA and RNA level data from TCGA

Somatic nucleotide-level mutation analysis was performed using whole-exome sequencing data from TCGA (The Cancer Genome Atlas; http://cancergenome.nih.gov/). BAM files (aligned to hg19 reference) for 150 TGCTs, including 18 pure ECs and 9 mixed with a dominant EC proportion, and their corresponding normal samples were downloaded from CGHub 2nd of February 2015 and analyzed for somatic mutations. In addition, clinical data was acquired including the International Classification of Diseases for Oncology (ICD-O) morphological codes (the latter being available for 134 of the 150 samples). Matched tumour and normal samples were compared in order to identify somatic SNVs using MuTect, v.1.1.7 (Cibulskis *et al.* 2013) and indels using Strelka v.1.0.14 (Saunders *et al.* 2012). All germline single nucleotide variants (SNVs) and indels were filtered out by this procedure. Candidate somatic mutations were annotated using ANNOVAR, version 2013-05-09 (Wang *et al.* 2010). A minimum of four variant reads in the tumour combined with zero variant reads in normal tissue were required to call a mutation. The resulting list of identified mutations was further filtered to retain only exonic events of the following types: non-synonymous SNVs, frameshift insertions and deletions, and gain or loss of stop codons.

DNA CNA data as output from GISTIC 2.0 analyses of the TGCT cohort from TCGA, downloaded from Firebrowse (http://www.firebrowse.org/) and the all_thresholded.by_genes.txt file was used to investigate CNAs of the 30 genes identified in regions with recurrent CNAs in the in-house cohort. For the CNA analysis, a subset of the 27 EC samples from TCGA was investigated. As analyzed by TCGA, the GISTIC cut off for gain

was 0.1 and for loss -0.1. High-level amplification and deep deletion cut off is calculated individually for each sample and listed in the GISTIC TCGA output file sample_cutoffs.txt. Correlations between DNA CNA and gene expression were analysed from gene expression data of the 150 TGCTs in the form of gene-level normalized RSEM and linear DNA CNA data from GISTIC 2.0 analysis. Spearman correlation analyses was performed between CNA and log2 transformed (RSEM + 1) mRNA expression values. P-values were corrected for multiple testing using the Benjamini-Hochberg method. Correlations with a corrected q-value < 0.05 were considered significant.

The presence of fusion genes was evaluated after download of RNA-sequencing data in form of FASTQ files, from the same 150 primary TGCTs from CGHub (dbGaP accession: phs000178.v9.p8). The FASTQ files were submitted to the fusion gene finder tools deFuse and FusionCatcher (McPherson *et al.* 2011, Nicorici *et al.* 2014). Hg19 sequence reference from UCSC and Ensembl release 69 was used for annotation of fusion breakpoints. For robustness, we retained only the fusions that were reported with breakpoints in the same two partner genes by both tools. Breakpoints within 1Mbp of the segments with focal regions of gain, loss, or LOH were identified. Further, we filtered the breakpoints according to a set of quality filters as previously described (Hoff *et al.* 2016).

References

Cibulskis, K, Lawrence, MS, Carter, SL, Sivachenko, A, Jaffe, D, Sougnez, C, Gabriel, S, Meyerson, M, Lander, ES & Getz, G 2013 Sensitive detection of somatic point mutations in impure and heterogeneous cancer samples. *Nat Biotech* 31 213-219.

Hoff, AM, Alagaratnam, S, Zhao, S, Bruun, J, Andrews, PW, Lothe, RA & Skotheim, RI 2016 Identification of Novel Fusion Genes in Testicular Germ Cell Tumors. *Cancer Research* 76 108-116.

McPherson, A, Hormozdiari, F, Zayed, A, Giuliany, R, Ha, G, Sun, MGF, Griffith, M, Moussavi, AH, Senz, J, Melnyk, N, *et al.* 2011 deFuse: An Algorithm for Gene Fusion Discovery in Tumor RNA-Seq Data. *Plos Computational Biology* 7 e1001138.

Nicorici, D, Satalan, M, Edgren, H, Kangaspeska, S, Murumagi, A, Kallioniemi, O, Virtanen, S & Kilkku, O 2014 FusionCatcher - a tool for finding somatic fusion genes in paired-end RNA-sequencing data. *bioRxiv* doi: https://doi.org/10.1101/011650.

Saunders, CT, Wong, WSW, Swamy, S, Becq, J, Murray, LJ & Cheetham, RK 2012 Strelka: accurate somatic small-variant calling from sequenced tumor–normal sample pairs. *Bioinformatics* 28 1811-1817.

Wang, K, Li, M & Hakonarson, H 2010 ANNOVAR: functional annotation of genetic variants from high-throughput sequencing data. *Nucleic Acids Research* 38 e164.
