# Supplementary figures and images for: Frequent copy number gains of SLC2A3 and ETV1 in testicular embryonal carcinomas
Source: Endocr Relat Cancer. 2020 Jun 10;27(9):457–68. doi: 10.1530/ERC-20-0064 (PMC7424350; doi:10.1530/ERC-20-0064)

Supplementary Figure 1

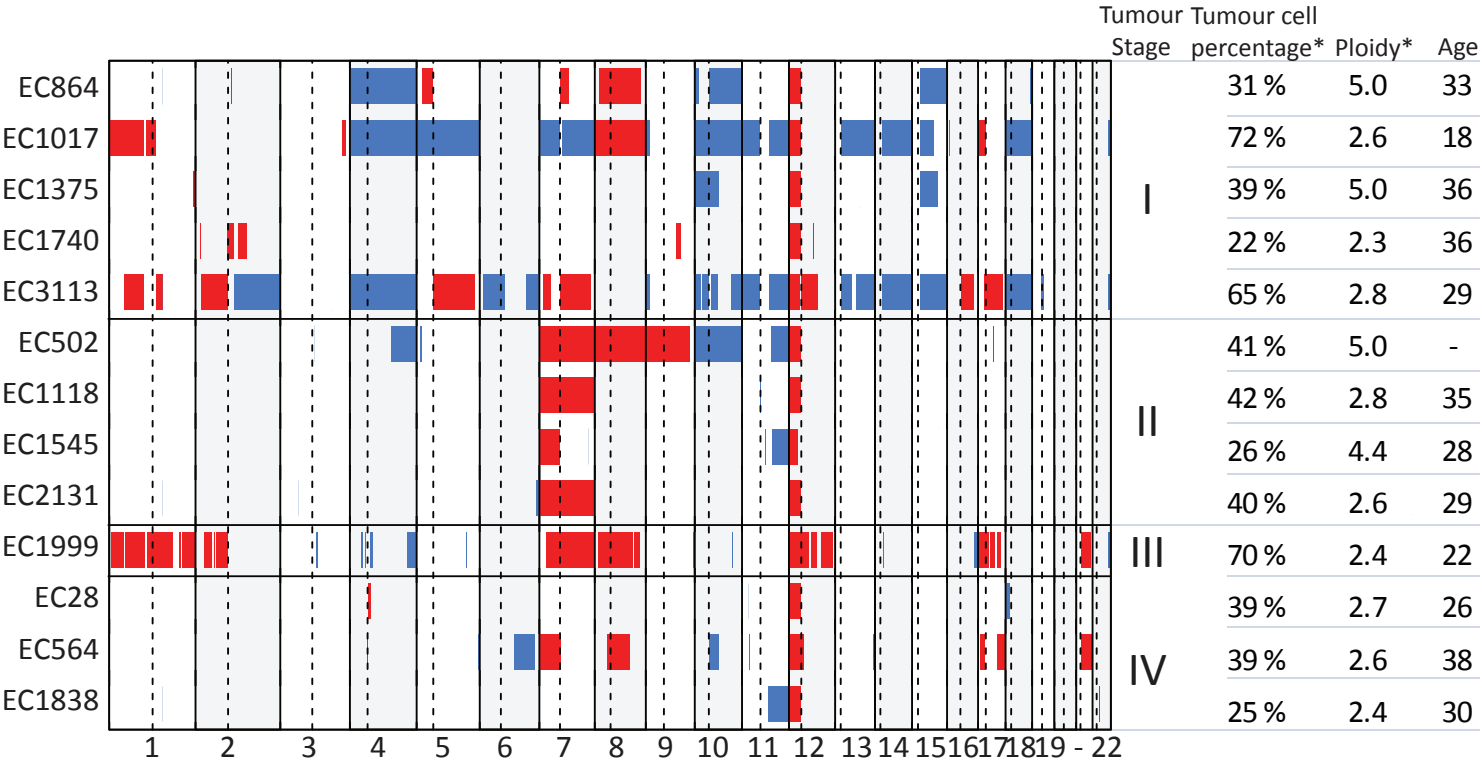

Supplement: Supplementary Figure 1. DNA copy number aberrations in 13 primary ECs. Regions with gain (log R ratio >0.15, red) and loss (log R ratio <-0.15, blue) after segmentation by PCF are plotted along the genome (horizontal axis: chromosome numbers). The ECs are grouped by tumour stage. *as determined by A [file supplementary_figure_1.pdf]

Supplementary Figure 2

A

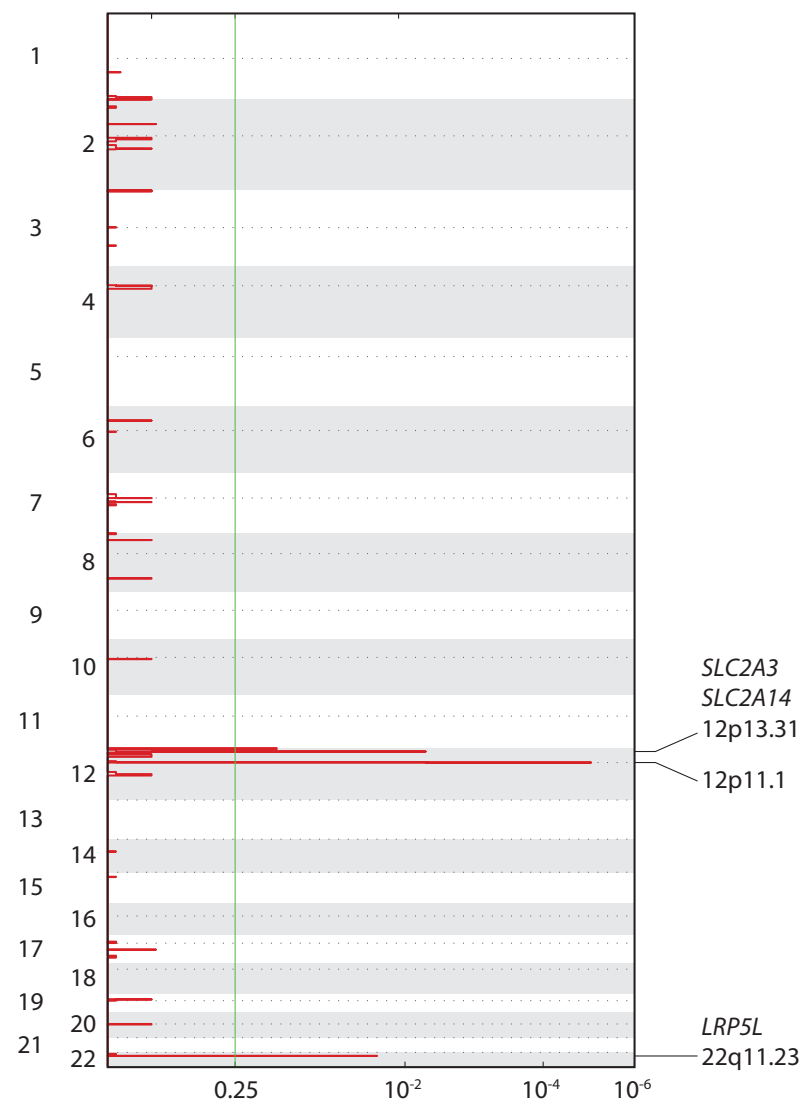

B

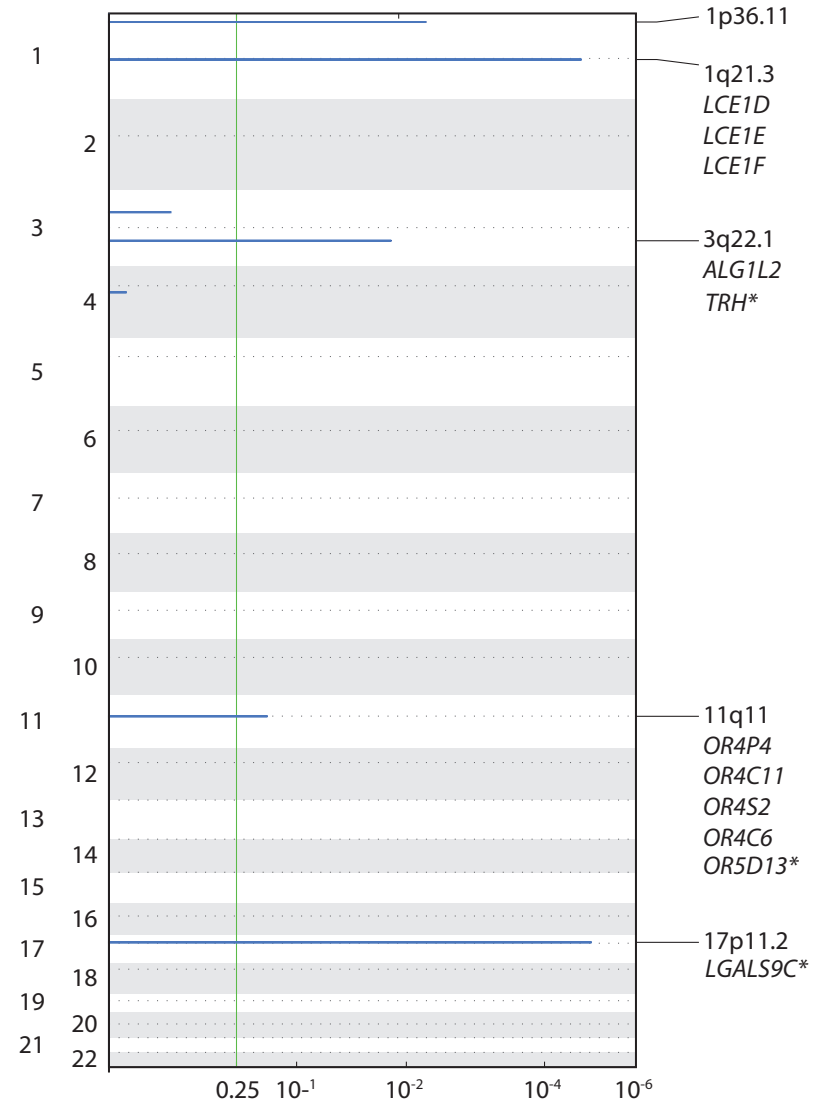

Supplement: Supplementary Figure 2. Amplified and deleted regions as called by GISTIC. (A) Focal regions of gain and (B) Focal regions of loss as identified in 13 primary ECs plotted by FDR q-value along the genome. Genes located in the significant regions (FDR-adjusted q < 0.25) are shown; * genes identified f [file supplementary_figure_2.pdf]

Supplementary Figure 3

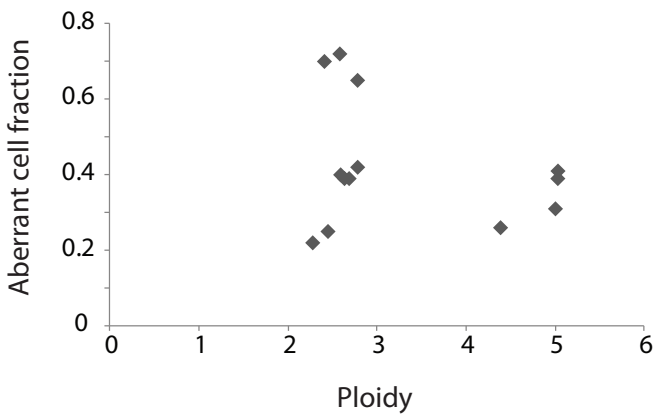

Supplement: Supplementary Figure 3. Ploidy and aberrant cell fraction. Estimated ploidy versus aberrant cell fraction, determined by ASCAT analysis, plotted for the 13 primary ECs. [file supplementary_figure_3.pdf]

Supplementary Figure 4

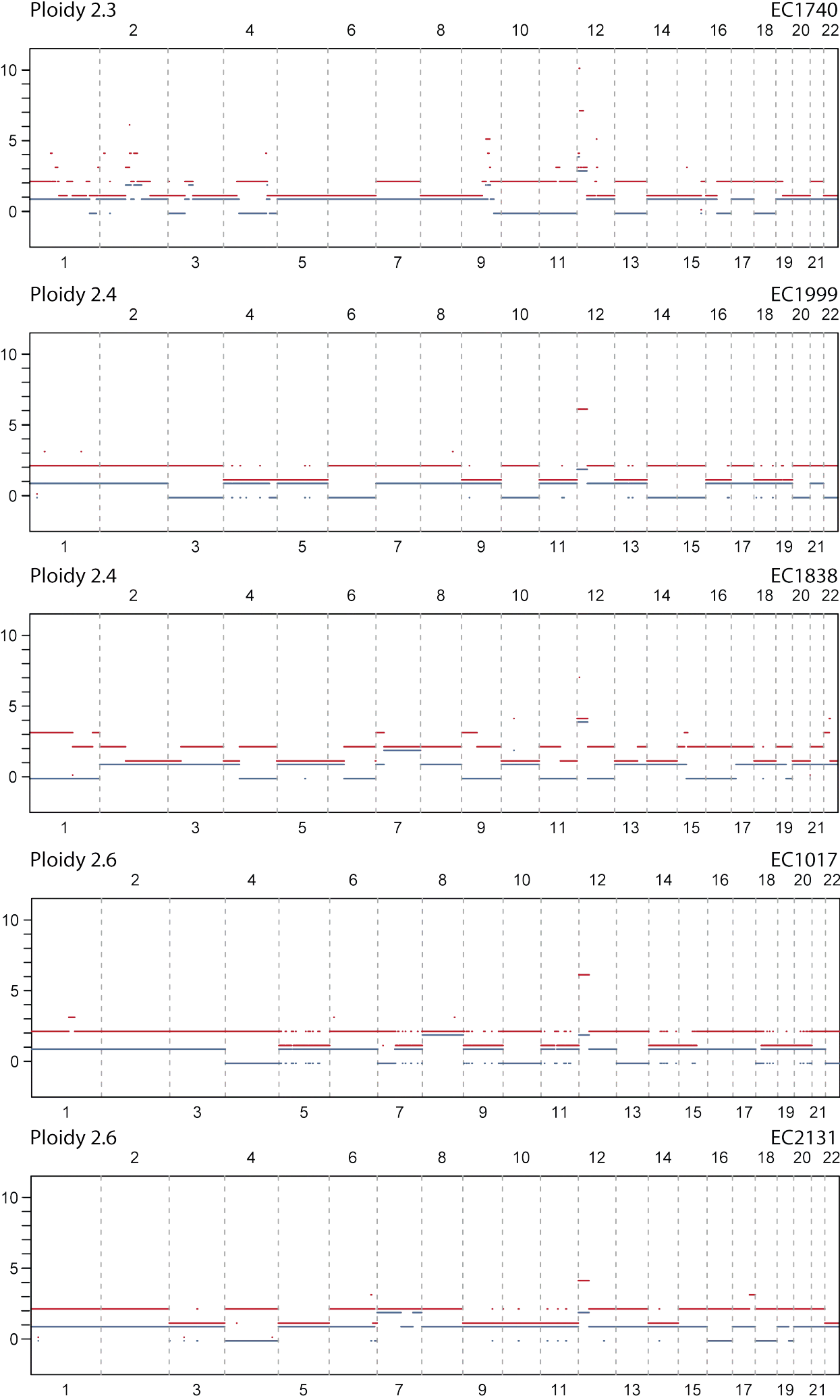

Ploidy 2.6

EC564

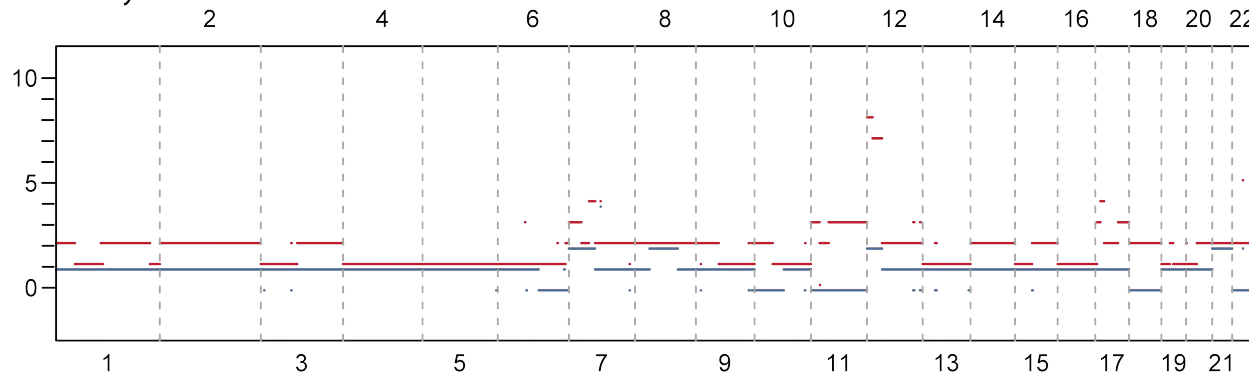

Ploidy 2.7

EC28

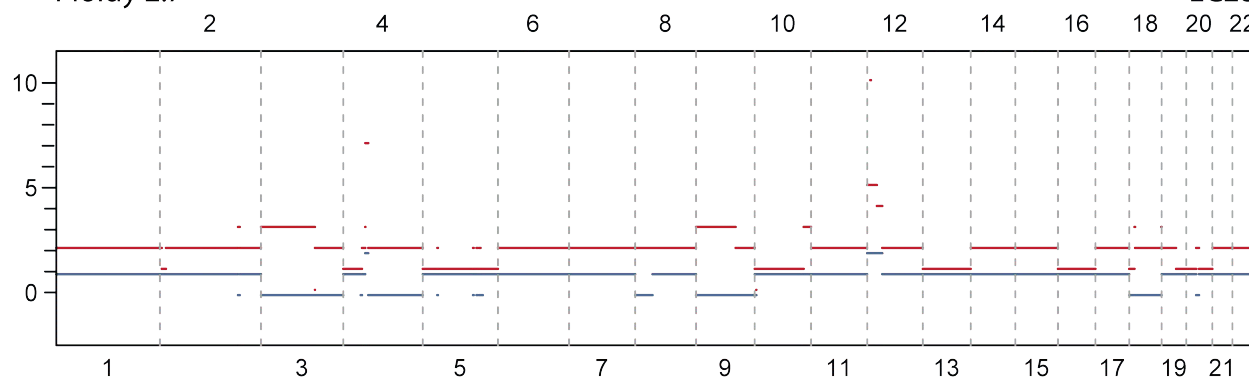

Ploidy 2.8

EC3113

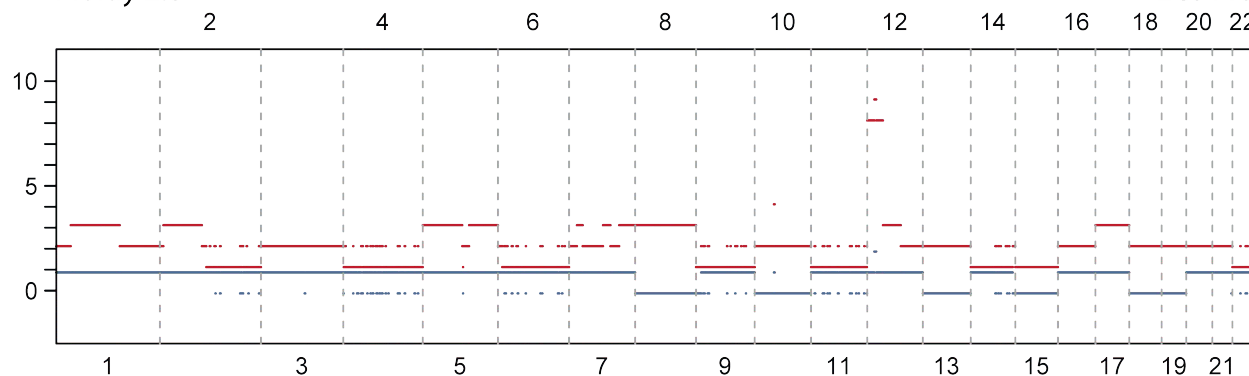

Ploidy 2.8

EC1118

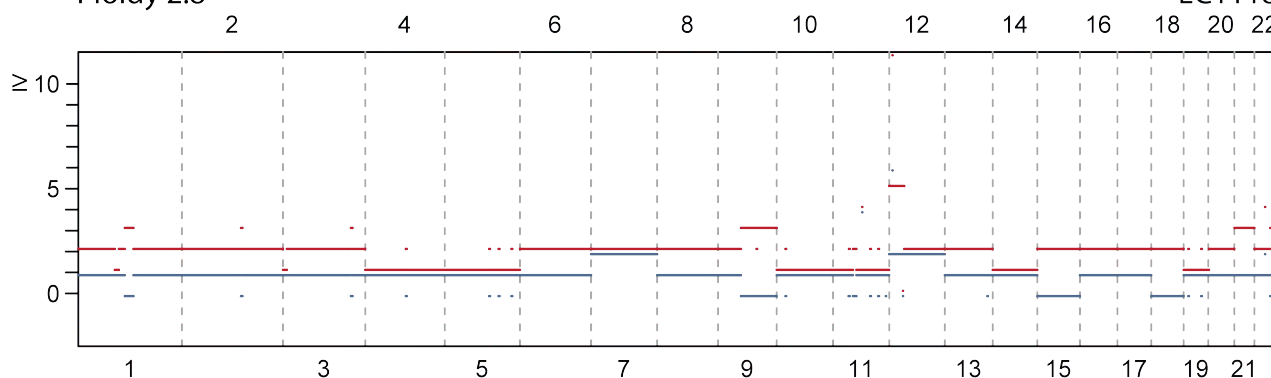

Ploidy 4.4

EC1545

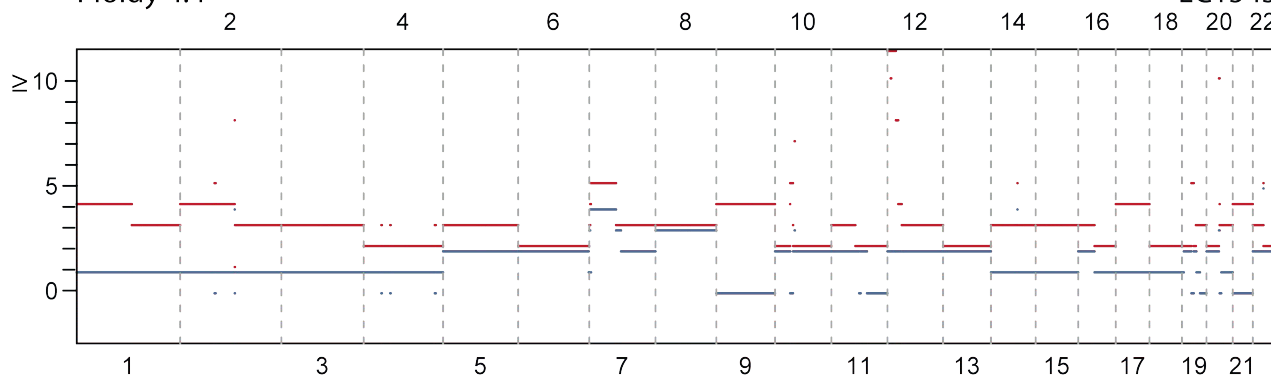

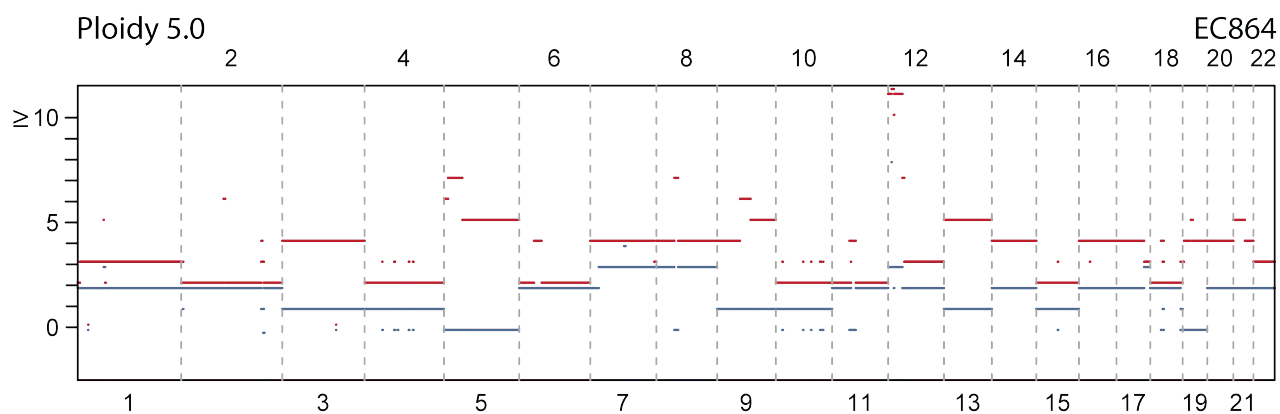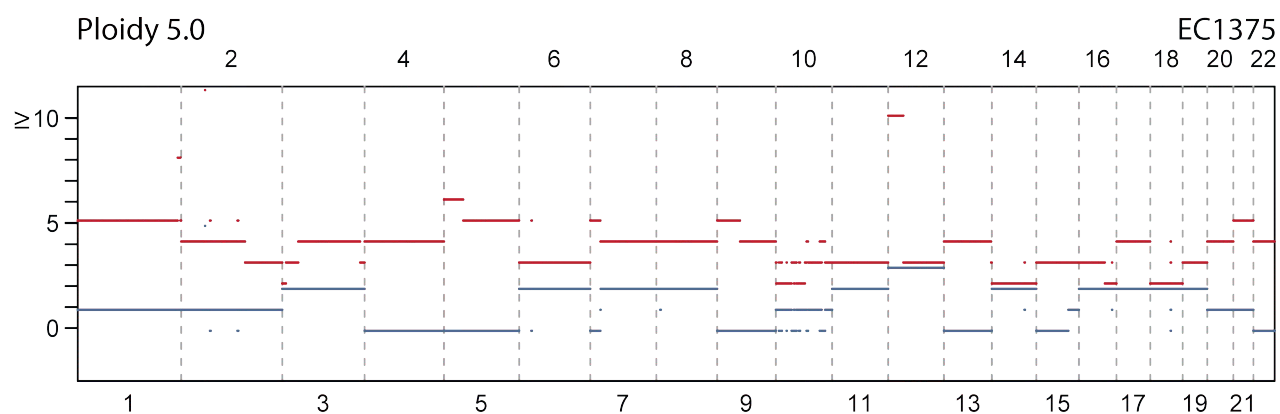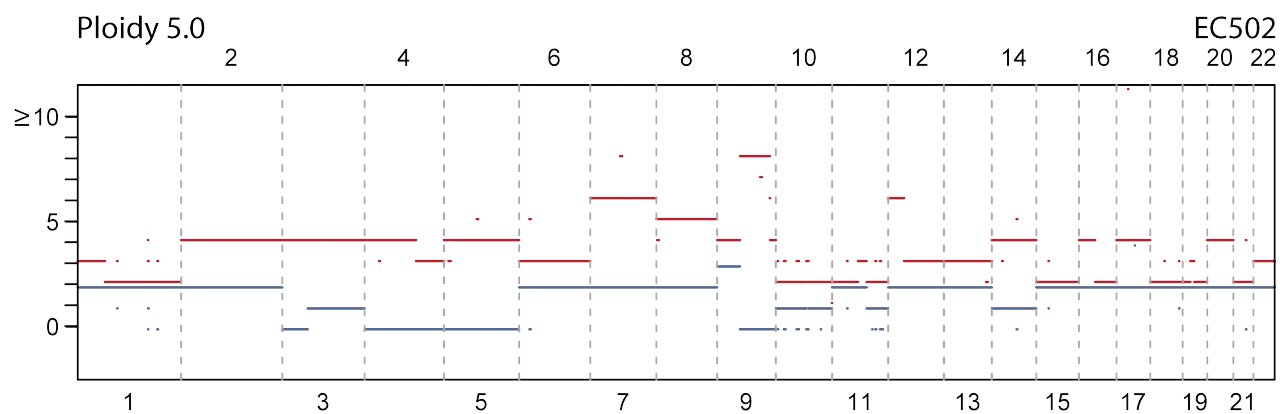

Supplement: Supplementary Figure 4. Individual allele-specific profiles of 13 primary ECs in order of increasing ploidy. Copy number aberrations (CNA) illustrated as regions of gain and loss along the genome (horizontal-axis) and absolute copy number (vertical-axis). The red and blue colours represent the two s [file supplementary_figure_4.pdf]

Supplementary Figure 5

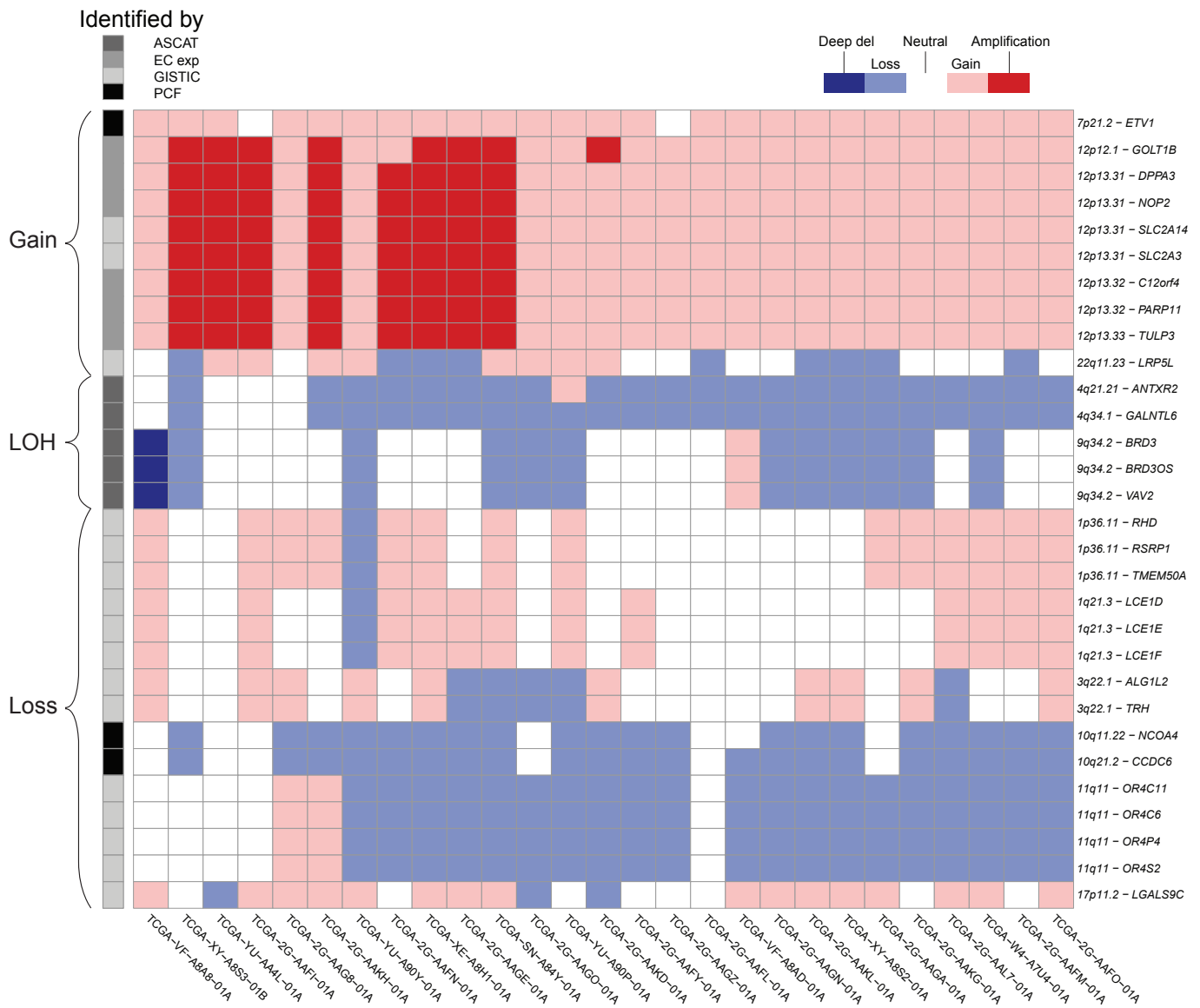

Supplement: : Supplementary Figure 5. CNAs of identified target genes in TCGA EC samples. CNA levels as determined by TCGA GISTIC analysis are shown for the identified target genes (n = 30) in 27 EC samples in the cohort from TCGA. The genes are clustered by the type of CNA affecting their respective genomic re [file supplementary_figure_5.pdf]

Supplementary Figure 7

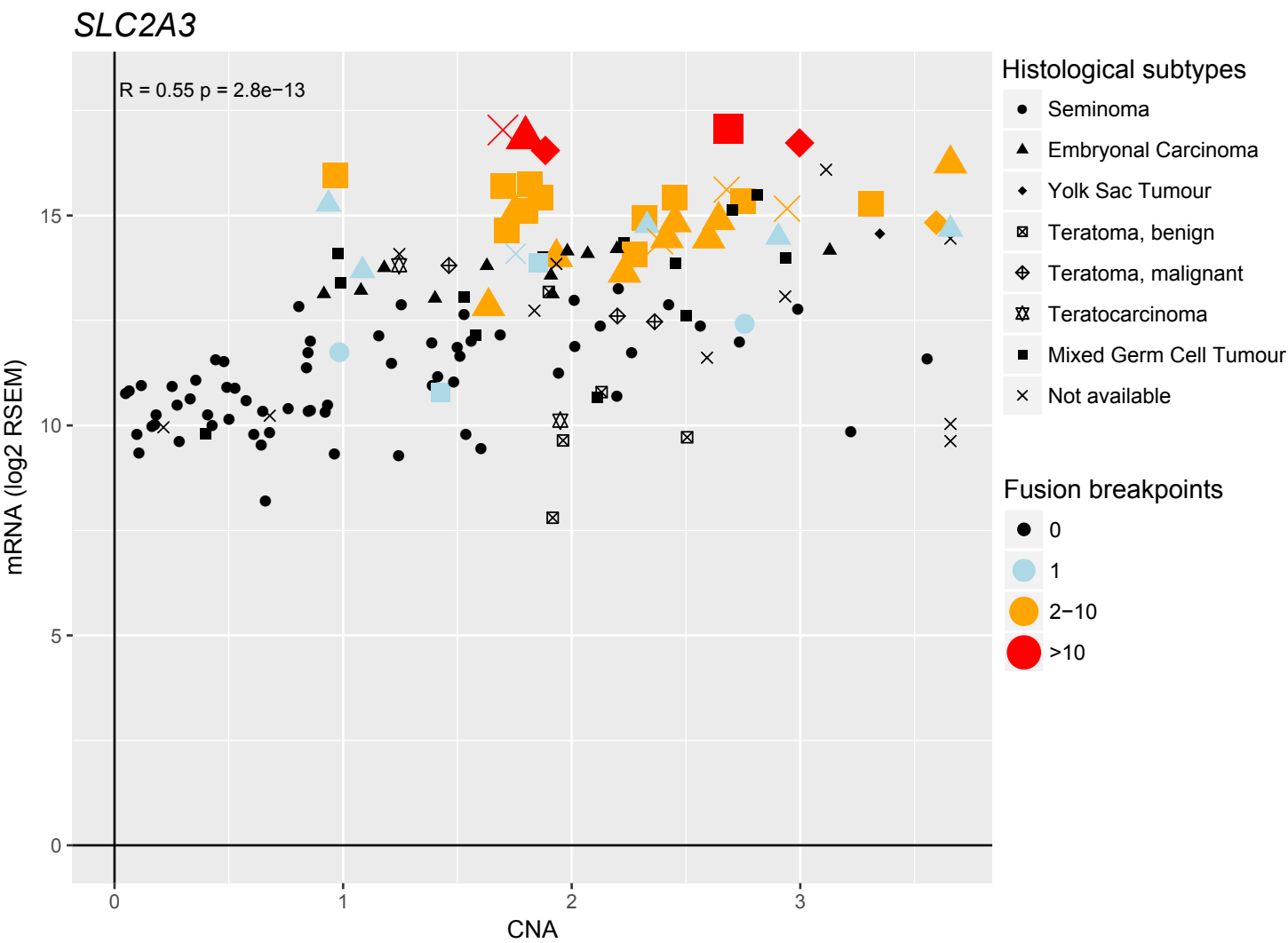

Supplement: Supplementary Figure 7. Fusion transcript breakpoints in SLC2A3 vs. copy number aberration (CNA) and gene expression among TGCTs. The log2 transformed SLC2A3 mRNA expression values are plotted against CNA per TGCT sample. The size and colour of each data point reflect the average number of fusion tr [file supplementary_figure_7.pdf]
