# Supplementary material for: Frequent copy number gains of SLC2A3 and ETV1 in testicular embryonal carcinomas
Source: Endocr Relat Cancer. 2020 Jun 10;27(9):457–68. doi: 10.1530/ERC-20-0064 (PMC7424350; doi:10.1530/ERC-20-0064)
Supplement: Supplementary Figure 6. TCGA gene expression vs. linear copy number. For 30 genes in regions of recurrent CNAs, the log2 transformed RSEM gene expression values are plotted against the DNA copy number for each sample in the TCGA cohort (N = 150). Significant correlations (i.e. corrected q values <0. [file supplementary_figure_6.pdf]

Supplementary Figure 6

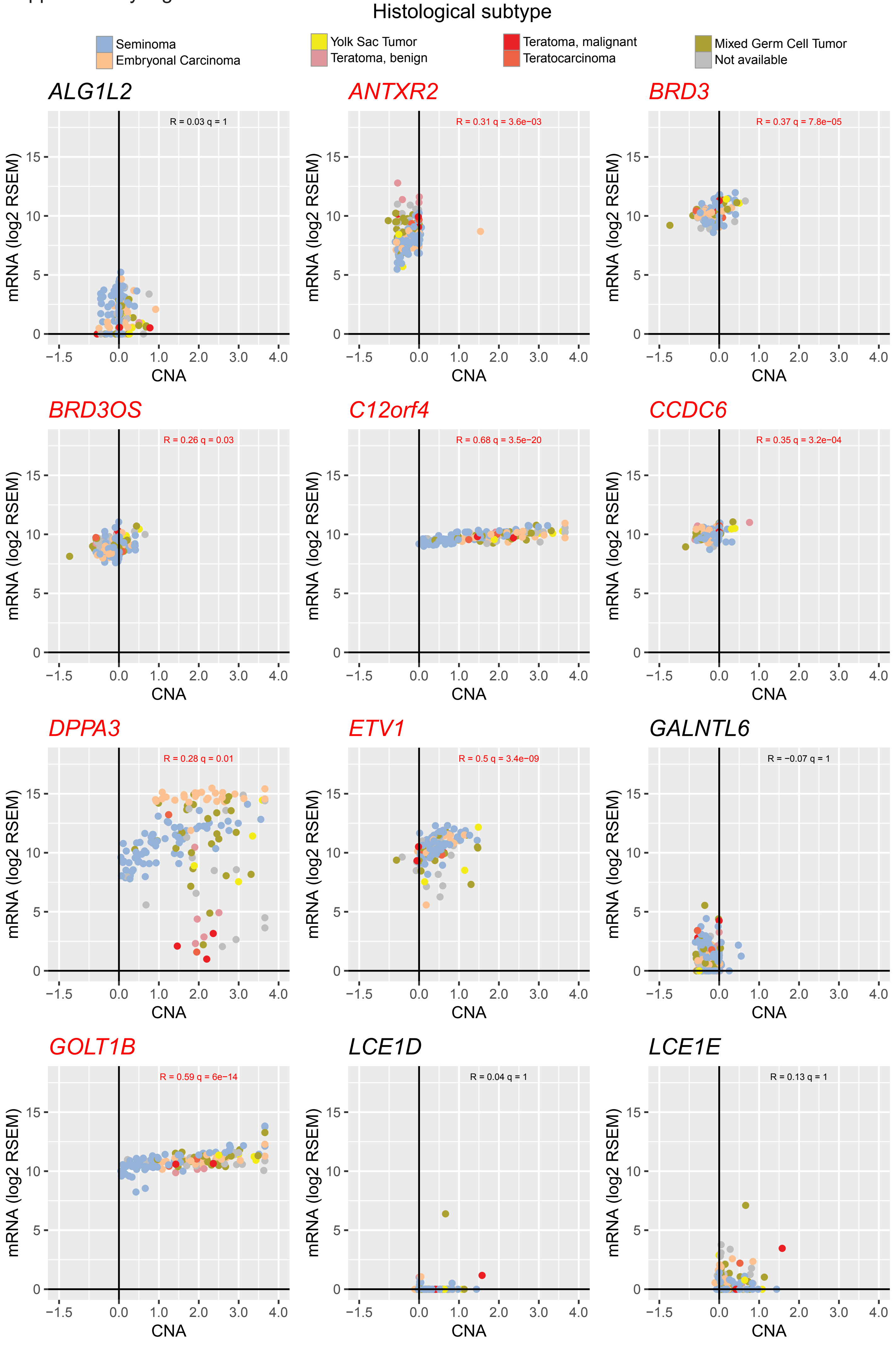

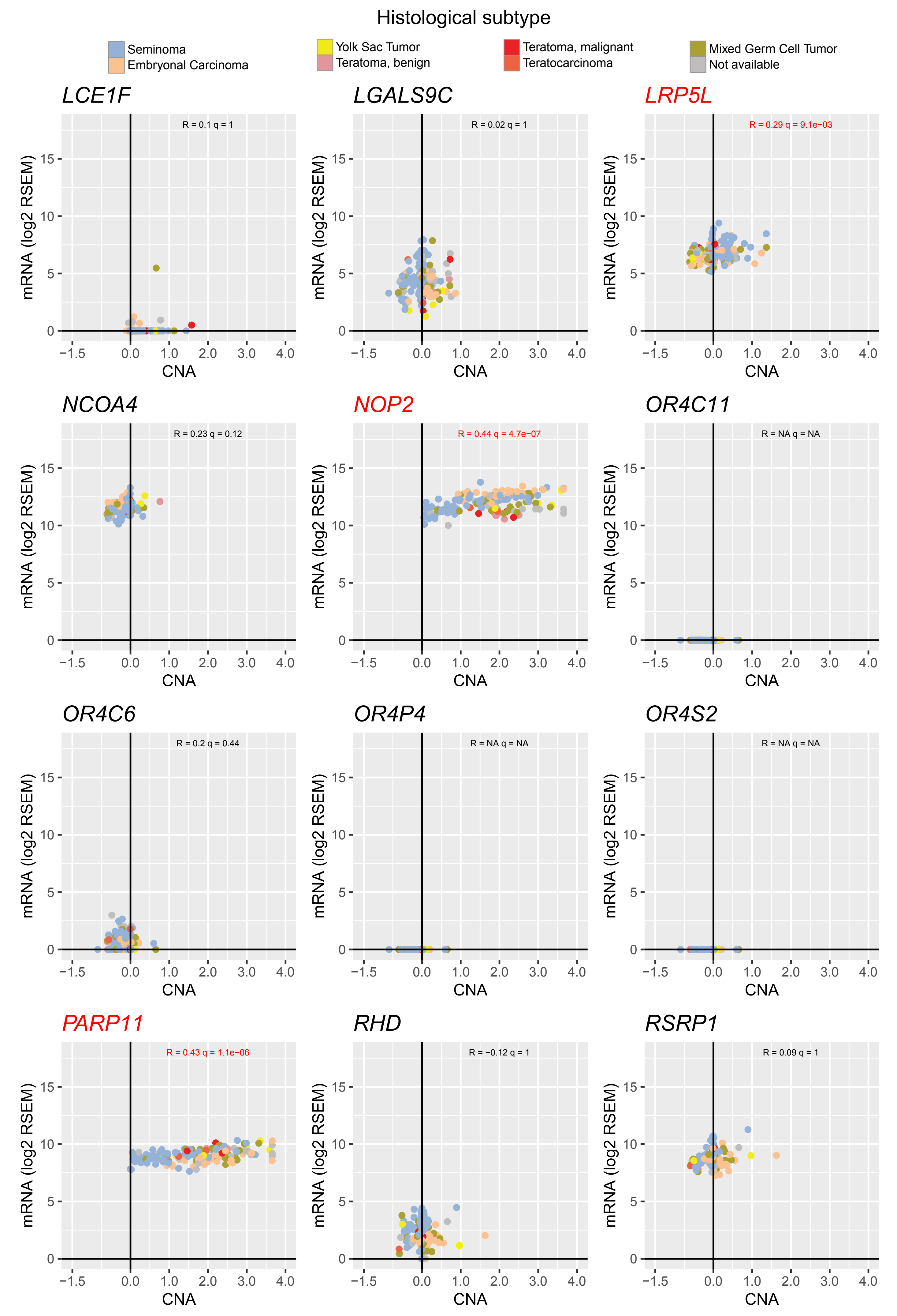
Histological subtype
Seminoma
Embryonal Carcinoma
Yolk Sac Tumor
Teratoma, benign
Teratoma, malignant
Teratocarcinoma
Mixed Germ Cell Tumor
Not available
LCE1F
R = 0.1 q = 1
LGALS9C
R = 0.02 q = 1
LRP5L
R = 0.29 q = 9.1e−03
NCOA4
R = 0.23 q = 0.12
NOP2
R = 0.44 q = 4.7e−07
OR4C11
R = NA q = NA
OR4C6
R = 0.2 q = 0.44
OR4P4
R = NA q = NA
OR4S2
R = NA q = NA
PARP11
R = 0.43 q = 1.1e−06
RHD
R = −0.12 q = 1
RSRP1
R = 0.09 q = 1
mRNA (log2 RSEM)
CNA
0
5
10
15
−1.5
0.0
1.0
2.0
3.0
4.0

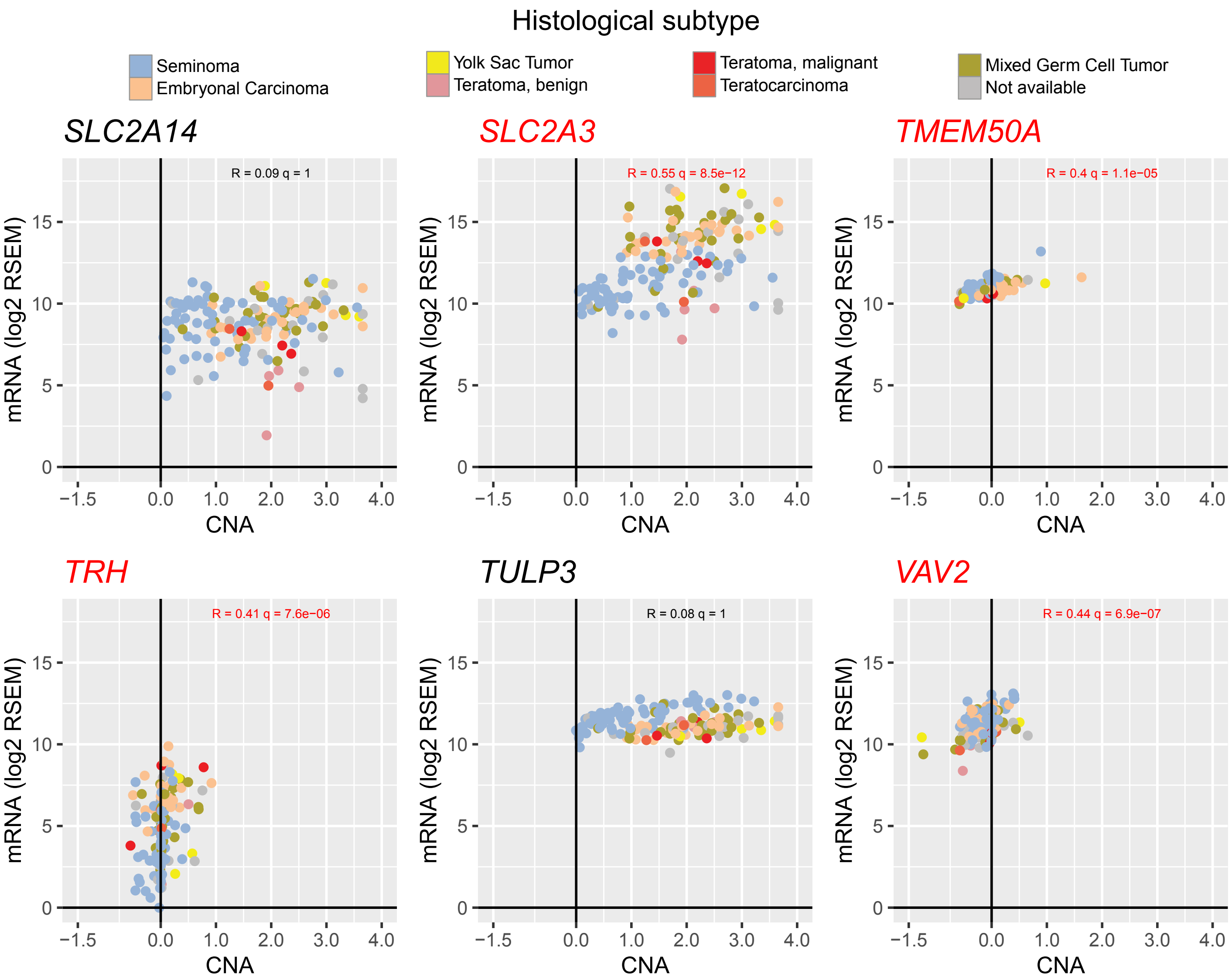

Histological subtype
Seminoma
Embryonal Carcinoma
Yolk Sac Tumor
Teratoma, benign
Teratoma, malignant
Teratocarcinoma
Mixed Germ Cell Tumor
Not available
SLC2A14
R = 0.09 q = 1
SLC2A3
R = 0.55 q = 8.5e−12
TMEM50A
R = 0.4 q = 1.1e−05
TRH
R = 0.41 q = 7.6e−06
TULP3
R = 0.08 q = 1
VAV2
R = 0.44 q = 6.9e−07
mRNA (log2 RSEM)
CNA
−1.5
0.0
1.0
2.0
3.0
4.0
0
5
10
15
