# Supplementary material for: Frequent copy number gains of SLC2A3 and ETV1 in testicular embryonal carcinomas
Source: Endocr Relat Cancer. 2020 Jun 10;27(9):457–68. doi: 10.1530/ERC-20-0064 (PMC7424350; doi:10.1530/ERC-20-0064)
Supplement: Supplementary Table 1. Statistically significant broad aberrations identified in primary ECs [file supplementary_table_1.pdf]

Supplementary Table 1. Statistically significant broad aberrations identified in primary ECs.

Gain		Loss	
Chromosome arm	q-value	Chromosome arm	q-value
7p	$3x10^{-6}$	4p	0.04
7q	$3x10^{-6}$	4q	0.007
8p	0.01	10p	0.04
8q	0.02	10q	0.02
12p	$2x10^{-7}$	11q	0.04
12q	0.03	18p	0.05
17q	0.02	18q	0.04
21q	0.002		
