# Supplementary material for: Frequent copy number gains of SLC2A3 and ETV1 in testicular embryonal carcinomas
Source: Endocr Relat Cancer. 2020 Jun 10;27(9):457–68. doi: 10.1530/ERC-20-0064 (PMC7424350; doi:10.1530/ERC-20-0064)
Supplement: Supplementary Table 2. Genes identified in regions of recurrent CNAs in EC [file supplementary_table_2.pdf]

Supplementary Table 2. Genes identified in regions of recurrent CNAs in EC

Gene	Approved name	Location	Identified by	CNA type
ALG1L2	ALG1, chitobiosyldiphosphodolichol beta-mannosyltransferase like 2	3q22.1	GISTIC	Loss
ANTXR2	ANTXR cell adhesion molecule 2	4q21.21	ASCAT	LOH
BRD3	Bromodomain containing 3	9q34.2	ASCAT	LOH
BRD3OS	BRD3 opposite strand	9q34.2	ASCAT	LOH
C12orf4	Chromosome 12 open reading frame 4	12p13.32	EC exp	Gain
CCDC6	Coiled-coil domain containing 6	10q21.2	PCF	Loss
DPPA3	Developmental pluripotency associated 3	12p13.31	EC exp	Gain
ETV1	ETS variant 1	7p21.2	PCF	Gain
GALNTL6	Polypeptide N-acetylgalactosaminyltransferase like 6	4q34.1	ASCAT	LOH
GOLT1B	Golgi transport 1B	12p12.1	EC exp	Gain
LCE1D	Late cornified envelope 1D	1q21.3	GISTIC	Loss
LCE1E	Late cornified envelope 1E	1q21.3	GISTIC	Loss
LCE1F	Late cornified envelope 1F	1q21.3	GISTIC	Loss
LGALS9C	Galectin 9C	17p11.2	GISTIC	Loss
LRP5L	LDL receptor related protein 5 like	22q11.23	GISTIC	Gain
NCOA4	Nuclear receptor coactivator 4	10q11.22	PCF	Loss
NOP2	NOP2 nucleolar protein	12p13.31	EC exp	Gain
OR4C11	Olfactory receptor family 4 subfamily C member 11	11q11	GISTIC	Loss
OR4C6	Olfactory receptor family 4 subfamily C member 6	11q11	GISTIC	Loss
OR4P4	Olfactory receptor family 4 subfamily P member 4	11q11	GISTIC	Loss
OR4S2	Olfactory receptor family 4 subfamily S member 2	11q11	GISTIC	Loss
PARP11	Poly(ADP-ribose) polymerase family member 11	12p13.32	EC exp	Gain
RHD	Rh blood group D antigen	1p36.11	GISTIC	Loss
RSRP1	Arginine and serine rich protein 1	1p36.11	GISTIC	Loss
SLC2A14	Solute carrier family 2 member 14	12p13.31	GISTIC	Gain
SLC2A3	Solute carrier family 2 member 3	12p13.31	GISTIC	Gain
TMEM50A	Transmembrane protein 50A	1p36.11	GISTIC	Loss
TRH	Thyrotropin releasing hormone	3q22.1	GISTIC	Loss
TULP3	Tubby like protein 3	12p13.33	EC exp	Gain
VAV2	Vav guanine nucleotide exchange factor 2	9q34.2	ASCAT	LOH

EC exp, previously identified with increased expression in EC vs EC cell line
