# Supplementary material for: Frequent copy number gains of SLC2A3 and ETV1 in testicular embryonal carcinomas
Source: Endocr Relat Cancer. 2020 Jun 10;27(9):457–68. doi: 10.1530/ERC-20-0064 (PMC7424350; doi:10.1530/ERC-20-0064)
Supplement: Supplementary Table 3. Somatic mutations identified in target genes [file supplementary_table_3.pdf]

Supplementary Table 3. Somatic mutations identified in target genes. Somatic mutations identified within the 30 candidate genes among 144 TGCTs from the TCGA whole-exome sequencing data set. International Classification of Diseases for Oncology (ICD-O) morphological codes are listed and corresponds to: 9061/3 = Seminoma (Sem), 9070/3 = Embryonal Carcinoma (EC), 9071/3 = Yolk Sac Tumour (YST), 9080/0 = Teratoma benign (TerB), 9080/3 = Teratoma malignant (Ter), 9081/3 = Teratocarcinoma (TerC), 9085/3 = Mixed Germ Cell Tumour (Mixed GCT), NA = Not Available from TCGA.

Gene	CNA type	Sample	ICD-O (histology)	Non-synonymous SNV
ALG1L2	Loss	TCGA-YU-AA61	9085/3 (Mixed GCT)	NM_001136152:c.T470G:p.L157R
ANTXR2	LOH	TCGA-2G-AAG5	9080/0 (TerB)	NM_001145794:c.C881T:p.S294L
		TCGA-2G-AAL7	9070/3 (EC)	NM_001145794:c.C860A:p.A287D
		TCGA-VF-A8AC	9061/3 (Sem)	NM_001145794:c.C677A:p.P226H
		TCGA-VF-A8AE	9061/3 (Sem)	NM_001145794:c.C820A:p.L274I
BRD3	LOH	TCGA-2G-AAGF	9085/3 (Mixed GCT)	NM_007371:c.71delC:p.P24fs
C12orf4	Gain	TCGA-X3-A8G4	9085/3 (Mixed GCT)	NM_020374:c.C119A:p.A40D
LCE1F	Loss	TCGA-2G-AAHP	9061/3 (Sem)	NM_178354:c.G193T:p.G65C
		TCGA-2G-AALZ	NA	NM_178354:c.G193T:p.G65C
		TCGA-XE-AAOB	9081/3 (TerC)	NM_178354:c.G193T:p.G65C
		TCGA-ZM-AA0H	9061/3 (Sem)	NM_178354:c.G194C:p.G65A
LGALS9C	Loss	TCGA-2G-AAHC	9061/3 (Sem)	NM_001040078:c.C596T:p.T199M
NOP2	Gain	TCGA-2G-AAFV	9071/3 (YST)	NM_001033714:c.G2268T:p.L756F
RHD	Loss	TCGA-2G-AAKH	9070/3 (EC)	NM_001127691:c.C173T:p.A58V
SLC2A14	Gain	TCGA-2G-AALF	NA	NM_001286236:c.G116A:p.R39H
		TCGA-2G-AALR	NA	NM_001286236:c.A235G:p.I79V
		TCGA-XE-AAOL	9061/3 (Sem)	NM_001286236:c.G772A:p.A258T
SLC2A3	Gain	TCGA-2G-AAKH	9070/3 (EC)	NM_006931:c.C740A:p.A247E
		TCGA-2G-AALR	NA	NM_006931:c.T305C:p.V102A
		TCGA-YU-A912	9061/3 (Sem)	NM_006931:c.T732G:p.D244E
TULP3	Gain	TCGA-2G-AAFL	9070/3 (EC)	NM_001160408:c.A609C:p.R203S
		TCGA-2G-AAGE	9070/3 (EC)	NM_001160408:c.G910T:p.A304S
