# Supplementary material for: Frequent copy number gains of SLC2A3 and ETV1 in testicular embryonal carcinomas
Source: Endocr Relat Cancer. 2020 Jun 10;27(9):457–68. doi: 10.1530/ERC-20-0064 (PMC7424350; doi:10.1530/ERC-20-0064)
Supplement: Supplementary Data and materials. PCF segmented copy number data from the in-house analysed EC cell lines (n = 5), primary EC samples (n = 13) and ES cell lines (n = 7). [file supplementary_table_4.pdf]

sampleID	chrom	arm	start.pos	end.pos	n.probes	mean
S10 833KE	1	p	61735	25894853	14441	0.2705
S10 833KE	1	p	25896756	66271102	24755	0.3802
S10 833KE	1	p	66271786	101224461	22776	0.3311
S10 833KE	1	p	101226749	107496929	3734	0.2645
S10 833KE	1	p	107497039	121482979	8943	0.3553
S10 833KE	1	q	143982530	185883507	24536	0.1147
S10 833KE	1	q	185885255	199505667	9003	0.0406
S10 833KE	1	q	199506093	215861469	11162	0.1435
S10 833KE	1	q	215861608	223677953	5578	0.0629
S10 833KE	1	q	223686843	249224388	17904	0.1212
S10 833KE	2	p	12784	16102573	12221	-0.2417
S10 833KE	2	p	16104325	22605038	4605	0.1006
S10 833KE	2	p	22605972	26606005	2279	0.379
S10 833KE	2	p	26612839	34691069	5235	0.1363
S10 833KE	2	p	34737163	36376131	1206	-0.2399
S10 833KE	2	p	36376427	49510455	9653	0.1355
S10 833KE	2	p	49512454	51837079	1730	0.0147
S10 833KE	2	p	51837753	89131067	23502	0.1169
S10 833KE	2	q	95327887	118131773	12752	0.0998
S10 833KE	2	q	118132313	123219486	3284	-0.195
S10 833KE	2	q	123219880	126794728	2388	-0.2768
S10 833KE	2	q	126797090	128002559	822	0.1104
S10 833KE	2	q	128002572	128569200	279	0.3733
S10 833KE	2	q	128569248	132571593	2169	0.1129
S10 833KE	2	q	132585113	152465513	12415	-0.2585

S10 833KE	2	q	152466325	155616284	2045	0.1633
S10 833KE	2	q	155616448	210838631	33504	0.0824
S10 833KE	2	q	210838759	215056348	2729	-0.0353
S10 833KE	2	q	215056495	243089456	18095	0.0573
S10 833KE	3	p	60345	4034368	3652	0.0246
S10 833KE	3	p	4034821	66752561	43281	0.1241
S10 833KE	3	p	66753918	90502862	14647	0.0664
S10 833KE	3	q	93519478	107945193	8394	0.061
S10 833KE	3	q	107946503	150614600	26490	0.1125
S10 833KE	3	q	150615096	180607504	18567	0.0494
S10 833KE	3	q	180613072	189045853	5399	0.1497
S10 833KE	3	q	189048167	192093975	2245	0.0131
S10 833KE	3	q	192094006	197896118	3285	0.1023
S10 833KE	4	p	68821	10274202	5898	-0.1555
S10 833KE	4	p	10274626	27362689	12427	-0.2344
S10 833KE	4	p	27363777	36512868	5787	-0.3147
S10 833KE	4	p	36519732	43015166	4363	-0.2197
S10 833KE	4	p	43018884	47253818	2408	-0.3217
S10 833KE	4	p	47256986	49658612	1183	-0.1897
S10 833KE	4	q	52685699	58944570	4315	-0.1824
S10 833KE	4	q	58946358	76183259	10255	-0.2943
S10 833KE	4	q	76184807	105030424	17811	-0.247
S10 833KE	4	q	105036776	108051500	1706	-0.3275
S10 833KE	4	q	108065751	108075558	33	-1.0363
S10 833KE	4	q	108076023	156565910	29309	-0.245
S10 833KE	4	q	156572107	191027923	22914	0.0601

S10 833KE	5	p	15532	46389273	31096	0.0808
S10 833KE	5	q	49432831	58647328	6152	0.0802
S10 833KE	5	q	58647909	58922981	233	-0.3172
S10 833KE	5	q	58923601	72871063	7466	0.0791
S10 833KE	5	q	72871904	83082663	6668	0.1429
S10 833KE	5	q	83086898	112992985	17832	0.0663
S10 833KE	5	q	112993214	115157715	1530	0.3124
S10 833KE	5	q	115158120	117155597	1607	0.4395
S10 833KE	5	q	117157841	124915593	5166	0.3138
S10 833KE	5	q	124918634	145406181	12296	0.5219
S10 833KE	5	q	145409070	152955075	5228	0.6705
S10 833KE	5	q	152955611	169778096	11823	-0.2602
S10 833KE	5	q	169779216	180790320	6882	-0.2015
S10 833KE	6	p	149661	14828654	11845	0.0673
S10 833KE	6	p	14828995	48199363	21563	0.1171
S10 833KE	6	p	48200110	58774716	6402	0.0548
S10 833KE	6	q	61886440	73566541	7388	0.0221
S10 833KE	6	q	73568874	107172439	21311	0.0712
S10 833KE	6	q	107174515	112727954	3486	0.1473
S10 833KE	6	q	112730624	171051005	38957	0.0655
S10 833KE	7	p	43259	14647419	9924	-0.2067
S10 833KE	7	p	14648873	19039875	3539	-0.302
S10 833KE	7	p	19041337	58019983	27586	-0.2075
S10 833KE	7	q	61063974	69739760	3782	-0.1974
S10 833KE	7	q	69742450	70244304	365	-0.7613
S10 833KE	7	q	70246912	78980026	4274	-0.176

S10 833KE	7	q	78980368	97239443	11722	-0.2688
S10 833KE	7	q	97240000	113008610	9146	-0.206
S10 833KE	7	q	113008823	126496513	8113	-0.2772
S10 833KE	7	q	126502405	133785177	4431	-0.1854
S10 833KE	7	q	133785195	133795634	26	-0.986
S10 833KE	7	q	133795665	159127004	16422	-0.2151
S10 833KE	8	p	31254	2446768	1890	-0.1672
S10 833KE	8	p	2447280	8138297	6171	-0.2833
S10 833KE	8	p	8140390	34875614	20679	-0.2255
S10 833KE	8	p	34878334	43824048	4956	0.1162
S10 833KE	8	q	46847534	67603100	12617	0.0817
S10 833KE	8	q	67603950	76476839	5689	0.1421
S10 833KE	8	q	76476935	93929810	10041	0.0632
S10 833KE	8	q	93933541	105394466	7159	0.1275
S10 833KE	8	q	105395583	113430419	4819	0.0462
S10 833KE	8	q	113430447	138191918	17671	0.1041
S10 833KE	8	q	138194708	146298155	4827	-0.2105
S10 833KE	9	p	46587	33233494	28065	-0.2236
S10 833KE	9	p	33234650	39110886	3474	-0.1668
S10 833KE	9	q	71006575	138958483	47797	-0.1916
S10 833KE	9	q	138958552	140642373	481	-0.6227
S10 833KE	9	q	140644310	141091394	280	-0.1989
S10 833KE	10	p	72759	3614912	3008	-0.2301
S10 833KE	10	p	3614997	3913834	305	0.0536
S10 833KE	10	p	3917602	4426887	513	-0.3026
S10 833KE	10	p	4427759	11829174	6348	0.0804

S10 833KE	10	p	11830185	27587531	11516	-0.2016
S10 833KE	10	p	27593427	27702509	91	-0.687
S10 833KE	10	p	27706601	39076221	7721	-0.243
S10 833KE	10	q	42433540	68754907	16880	-0.2472
S10 833KE	10	q	68762083	81793094	8348	-0.1693
S10 833KE	10	q	81799186	93915187	8567	-0.2389
S10 833KE	10	q	93921332	106249477	7318	-0.1544
S10 833KE	10	q	106252200	135506704	21439	-0.2275
S10 833KE	11	p	198510 5787405	3320	0.1415	
S10 833KE	11	p	5788080	5809139	32	-0.6685
S10 833KE	11	p	5809338	19911017	10473	0.1246
S10 833KE	11	p	19911733	43284854	16825	0.0632
S10 833KE	11	p	43287399	51564427	4093	0.1195
S10 833KE	11	q	54701645	61627606	4017	0.106
S10 833KE	11	q	61629122	78760833	8756	0.1668
S10 833KE	11	q	78761451	111743654	23227	0.0699
S10 833KE	11	q	111749349	134944770	17268	0.108
S10 833KE	12	p	150442 11503975	7476	0.9287	
S10 833KE	12	p	11511577	11543406	32	-0.0449
S10 833KE	12	p	11552192	18574897	4907	0.8893
S10 833KE	12	p	18574987	20795813	1487	1.0197
S10 833KE	12	p	20795898	31265057	7776	0.8921
S10 833KE	12	p	31269298	31394424	86	1.4027
S10 833KE	12	p	31395472	34854498	2153	0.9468
S10 833KE	12	q	37857751	41808814	2252	0.2705
S10 833KE	12	q	41808863	47127866	3295	0.5058

S10 833KE	12	q	47132564	58775715	6700	0.1648
S10 833KE	12	q	58776060	83102036	15820	0.106
S10 833KE	12	q	83102325	93828205	6551	0.0301
S10 833KE	12	q	93830540	133778189	27358	0.1231
S10 833KE	13	q	19026949	49836183	22034	-0.2287
S10 833KE	13	q	49836644	56131816	3635	-0.1543
S10 833KE	13	q	56136301	93762345	23859	-0.2403
S10 833KE	13	q	93762828	115108397	15597	-0.1712
S10 833KE	14	q	20425911	100106362	52022	-0.2156
S10 833KE	14	q	100114256	106008645	2780	-0.153
S10 833KE	15	q	22752520	46020694	14216	-0.2452
S10 833KE	15	q	46020873	80855842	22548	0.0816
S10 833KE	15	q	80856281	102469040	15360	0.3099
S10 833KE	16	p	60777	31960151	20324	-0.1821
S10 833KE	16	q	46463782	66294812	13636	-0.2227
S10 833KE	16	q	66295179	71386890	2426	-0.1019
S10 833KE	16	q	71391170	89869393	15811	-0.2015
S10 833KE	16	q	89875218	90287535	170	0.1061
S10 833KE	17	p	526	22235650	13237	-0.1669
S10 833KE	17	q	25270411	43385349	10169	-0.172
S10 833KE	17	q	43387385	50184174	3661	0.1671
S10 833KE	17	q	50185153	54155864	2797	0.045
S10 833KE	17	q	54156181	66916744	7395	0.1488
S10 833KE	17	q	66918829	70140719	2399	0.06
S10 833KE	17	q	70142118	81048659	6181	0.1608
S10 833KE	18	p	11543	2809880	2005	-0.7131

S10 833KE	18	p	2811400	15402408	8579	-0.2205
S10 833KE	18	q	18529353	56873528	25245	-0.2225
S10 833KE	18	q	56874453	78015057	15547	-0.266
S10 833KE	19	p	90910	24596750	11441	-0.1224
S10 833KE	19	q	27747993	33454662	4033	-0.2241
S10 833KE	19	q	33456260	59097854	14321	-0.1275
S10 833KE	20	p	61305	26305579	20211	0.1164
S10 833KE	20	q	29420352	62956153	22679	0.1276
S10 833KE	21	q	14345669	14838857	194	-0.174
S10 833KE	21	q	14843717	29939436	11132	0.0693
S10 833KE	21	q	29940627	34055388	2934	0.3968
S10 833KE	21	q	34058867	34598385	370	0.1044
S10 833KE	21	q	34599892	36004539	969	0.3738
S10 833KE	21	q	36008128	39200997	2318	0.1157
S10 833KE	21	q	39203118	48096957	6666	-0.1917
S10 833KE	22	q	16052528	47295437	20819	-0.1628
S10 833KE	22	q	47295877	51234455	3129	-0.2322
S10 833KE	X	p	168477	25940695	16203	-0.1867
S10 833KE	X	p	25940771	45717720	12189	-0.2449
S10 833KE	X	p	45719108	58561930	5642	-0.1686
S10 833KE	X	q	61728829	80755536	8507	-0.1963
S10 833KE	X	q	80758901	95716912	8260	-0.255
S10 833KE	X	q	95719755	140388078	25202	-0.1898
S10 833KE	X	q	140391529	146609291	4383	-0.2619
S10 833KE	X	q	146609390	155233846	4785	-0.1725
S10 833KE	Y	p	179542	6101425	834	-1.2041

S10 833KE	Y	p	6107901	10076242	1911	-1.5451
S10 833KE	Y	q	13134531	59018259	6034	-1.5881
S11 H7	1	p	61735	98038346	60001	0.0051
S11 H7	1	p	98039971	111375566	8268	-0.0372
S11 H7	1	p	111376081	111388226	42	-0.7119
S11 H7	1	p	111392067	121482979	6338	0.0025
S11 H7	1	q	143982530	152757690	1924	0.0335
S11 H7	1	q	152759678	152768700	38	-1.4561
S11 H7	1	q	152773905	249224388	66221	-0.0139
S11 H7	2	p	12784	87361172	59753	-0.0121
S11 H7	2	p	87373893	88181244	210	0.4202
S11 H7	2	p	88191907	89131067	468	0.0434
S11 H7	2	q	95327887	242783396	90340	-0.0146
S11 H7	2	q	242915466	243034686	113	-0.6115
S11 H7	2	q	243037253	243089456	29	0.0375
S11 H7	3	p	60345	61222987	42453	-0.0057
S11 H7	3	p	61232197	66740869	4466	0.0689
S11 H7	3	p	66743062	90502862	14661	-0.0103
S11 H7	3	q	93519478	166056741	44299	-0.0135
S11 H7	3	q	166057166	166378015	169	0.3424
S11 H7	3	q	166382177	197896118	19912	-0.0022
S11 H7	4	p	68821	32052675	21381	-0.0082
S11 H7	4	p	32054591	49658612	10685	-0.0444
S11 H7	4	q	52685699	191027923	86343	-0.0144
S11 H7	5	p	15532	46389273	31096	-0.0185
S11 H7	5	q	49432831	180790320	82883	-0.003

S11	H7	6	p	149661	58774716	39810	-0.0075
S11	H7	6	q	61886440	89241977	17295	-0.0305
S11	H7	6	q	89242641	95442761	4178	0.0295
S11	H7	6	q	95452264	95533338	61	-0.7536
S11	H7	6	q	95540519	171051005	49608	-0.0055
S11	H7	7	p	43259	58019983	41049	-0.0063
S11	H7	7	q	61063974	159127004	58281	0.0016
S11	H7	8	p	31254	24974443	21954	-0.0085
S11	H7	8	p	24974476	24984333	26	-1.1191
S11	H7	8	p	24991104	43824048	11716	0.0107
S11	H7	8	q	46847534	131745564	52788	-0.0148
S11	H7	8	q	131745859	146298155	10035	-0.0587
S11	H7	9	p	46587	39110886	31539	-0.0141
S11	H7	9	q	71006575	141091394	48558	-0.0057
S11	H7	10	p	72759	39076221	29502	-0.0199
S11	H7	10	q	42433540	135506704	62552	-0.0109
S11	H7	11	p	198510	51564427	34743	-0.0159
S11	H7	11	q	54701645	134944770	53268	-0.01
S11	H7	12	p	150442	34854498	23917	0.002
S11	H7	12	q	37857751	133778189	61976	-0.0144
S11	H7	13	q	19026949	115108397	65125	-0.0117
S11	H7	14	q	20425911	106008645	54802	-0.0083
S11	H7	15	q	22752520	102469040	52124	-0.0016
S11	H7	16	p	60777	31960151	20324	-0.0125
S11	H7	16	q	46463782	90287535	32043	-0.0069
S11	H7	17	p	526	22235650	13237	0.0025

S11 H7	17	q	25270411	81048659	32602	0
S11 H7	18	p	11543	15402408	10584	-0.004
S11 H7	18	q	18529353	56874453	25246	0.0048
S11 H7	18	q	56874519	78015057	15546	-0.0413
S11 H7	19	p	90910	24596750	11441	0.0041
S11 H7	19	q	27747993	59097854	18354	-0.0042
S11 H7	20	p	61305	26305579	20211	-0.0019
S11 H7	20	q	29420352	62956153	22679	-0.0149
S11 H7	21	q	14345669	48096957	24583	-0.0177
S11 H7	22	q	16052528	47289798	20808	0.0172
S11 H7	22	q	47290259	51234455	3140	-0.0479
S11 H7	X	p	168477	58561930	34034	-0.0243
S11 H7	X	q	61728829	155233846	51137	-0.0117
S11 H7	Y	p	179542	6101425	834	-1.2161
S11 H7	Y	p	6107901	10076242	1911	-1.6883
S11 H7	Y	q	13134531	59018259	6034	-1.7061
S12 Shef4	1	p	61735	121482979	74649	0.0011
S12 Shef4	1	q	143982530	249224388	68183	-0.0118
S12 Shef4	2	p	12784	89131067	60431	-0.0118
S12 Shef4	2	q	95327887	243089456	90482	-0.0124
S12 Shef4	3	p	60345	61490149	42629	-0.0135
S12 Shef4	3	p	61494252	66752561	4304	0.0728
S12 Shef4	3	p	66753918	90502862	14647	-0.0144
S12 Shef4	3	q	93519478	197896118	64380	-0.0154
S12 Shef4	4	p	68821	31514574	21060	-0.0113
S12 Shef4	4	p	31515560	49658612	11006	-0.0508

S12 Shef4	4	q	52685699	191027923	86343	-0.0208
S12 Shef4	5	p	15532	46389273	31096	-0.0218
S12 Shef4	5	q	49432831	180790320	82883	-0.0119
S12 Shef4	6	p	149661	58774716	39810	-0.0137
S12 Shef4	6	q	61886440	89237770	17289	-0.0377
S12 Shef4	6	q	89238395	112727954	14896	0.0123
S12 Shef4	6	q	112730624	171051005	38957	-0.0215
S12 Shef4	7	p	43259	58019983	41049	-0.0132
S12 Shef4	7	q	61063974	159127004	58281	-0.0076
S12 Shef4	8	p	31254	43824048	33696	-0.0115
S12 Shef4	8	q	46847534	146298155	62823	-0.0276
S12 Shef4	9	p	46587	39110886	31539	-0.0149
S12 Shef4	9	q	71006575	141091394	48558	-0.004
S12 Shef4	10	p	72759	39076221	29502	-0.0205
S12 Shef4	10	q	42433540	68340012	16556	-0.0179
S12 Shef4	10	q	68340454	68564280	178	-0.6059
S12 Shef4	10	q	68564468	106249267	24378	0.0053
S12 Shef4	10	q	106249477	113872127	5579	-0.0577
S12 Shef4	10	q	113872457	135506704	15861	-0.0072
S12 Shef4	11	p	198510	51564427	34743	-0.0154
S12 Shef4	11	q	54701645	134944770	53268	-0.0091
S12 Shef4	12	p	150442	34854498	23917	-0.0022
S12 Shef4	12	q	37857751	133778189	61976	-0.0122
S12 Shef4	13	q	19026949	115108397	65125	-0.0065
S12 Shef4	14	q	20425911	106008645	54802	-0.0068
S12 Shef4	15	q	22752520	76891241	34220	0.0063

S12 Shef4	15	q	76891511	76895775	21	-0.8723
S12 Shef4	15	q	76902179	102469040	17883	-0.0161
S12 Shef4	16	p	60777	31960151	20324	-0.0079
S12 Shef4	16	q	46463782	90287535	32043	-0.0048
S12 Shef4	17	p	526	22235650	13237	0.0015
S12 Shef4	17	q	25270411	81048659	32602	0
S12 Shef4	18	p	11543	15402408	10584	-5.00E-04
S12 Shef4	18	q	18529353	56834300	25218	0.0037
S12 Shef4	18	q	56834655	78015057	15574	-0.0457
S12 Shef4	19	p	90910	24596750	11441	-0.0049
S12 Shef4	19	q	27747993	59097854	18354	-0.0054
S12 Shef4	20	p	61305	26305579	20211	-0.0023
S12 Shef4	20	q	29420352	62956153	22679	-0.0117
S12 Shef4	21	q	14345669	48096957	24583	-0.0195
S12 Shef4	22	q	16052528	47258435	20785	0.0123
S12 Shef4	22	q	47260154	51234455	3163	-0.0526
S12 Shef4	X	p	168477	2703391	428	-0.0018
S12 Shef4	X	p	2703633	58561930	33606	-0.58
S12 Shef4	X	q	61728829	88440167	13137	-0.5757
S12 Shef4	X	q	88441135	92423203	1668	-0.4482
S12 Shef4	X	q	92424029	139920048	26900	-0.5612
S12 Shef4	X	q	139920866	146769450	4769	-0.6304
S12 Shef4	X	q	146770081	154963439	4627	-0.5586
S12 Shef4	X	q	154964055	155233846	36	0.0427
S12 Shef4	Y	p	179542	10076242	2745	-8.00E-04
S12 Shef4	Y	q	13134531	59018259	6034	-0.0291

S13 Shef7	1	p	61735	62519088	36444	0.0166
S13 Shef7	1	p	62522561	65818709	2433	0.112
S13 Shef7	1	p	65821164	121482979	35772	0.0074
S13 Shef7	1	q	143982530	249224388	68183	0.0036
S13 Shef7	2	p	12784	89131067	60431	0.0049
S13 Shef7	2	q	95327887	212588820	70781	0.0094
S13 Shef7	2	q	212590966	243089456	19701	-0.0259
S13 Shef7	3	p	60345	61450007	42598	-0.0073
S13 Shef7	3	p	61455419	66799019	4380	0.0911
S13 Shef7	3	p	66799169	90502862	14602	-0.01
S13 Shef7	3	q	93519478	129762859	21803	-0.0128
S13 Shef7	3	q	129763698	129806236	55	-0.6964
S13 Shef7	3	q	129806924	197896118	42522	-0.0107
S13 Shef7	4	p	68821	31627384	21124	-0.0092
S13 Shef7	4	p	31629807	37555712	3755	-0.0908
S13 Shef7	4	p	37556482	49658612	7187	-0.0205
S13 Shef7	4	q	52685699	191027923	86343	-0.0125
S13 Shef7	5	p	15532	46389273	31096	-0.0147
S13 Shef7	5	q	49432831	95704305	27341	0.0023
S13 Shef7	5	q	95709170	104827539	5536	-0.0613
S13 Shef7	5	q	104832796	145209444	25923	0.0034
S13 Shef7	5	q	145210565	147509843	1608	0.1054
S13 Shef7	5	q	147510006	180790320	22475	-0.0164
S13 Shef7	6	p	149661	58774716	39810	-0.0038
S13 Shef7	6	q	61886440	77436532	9820	-0.0399
S13 Shef7	6	q	77439868	77452804	22	-1.0299

S13 Shef7	6	q	77461073	171051005	61300	-0.0079
S13 Shef7	7	p	43259	58019983	41049	-0.0013
S13 Shef7	7	q	61063974	159127004	58281	-0.005
S13 Shef7	8	p	31254	43824048	33696	-0.0094
S13 Shef7	8	q	46847534	133668391	54277	-0.0143
S13 Shef7	8	q	133668694	146298155	8546	-0.0694
S13 Shef7	9	p	46587	39110886	31539	0.0074
S13 Shef7	9	q	71006575	136119527	46173	0.018
S13 Shef7	9	q	136126177	141091394	2385	-0.0508
S13 Shef7	10	p	72759	39076221	29502	-0.0042
S13 Shef7	10	q	42433540	135506704	62552	0.0026
S13 Shef7	11	p	198510	51564427	34743	-8.00E-04
S13 Shef7	11	q	54701645	129832270	49282	-2.00E-04
S13 Shef7	11	q	129832825	134944770	3986	0.0512
S13 Shef7	12	p	150442	34854498	23917	0.0166
S13 Shef7	12	q	37857751	133778189	61976	0.004
S13 Shef7	13	q	19026949	115108397	65125	0.0093
S13 Shef7	14	q	20425911	106008645	54802	0.0042
S13 Shef7	15	q	22752520	102469040	52124	0.0111
S13 Shef7	16	p	60777	31960151	20324	0.004
S13 Shef7	16	q	46463782	90287535	32043	-1.00E-04
S13 Shef7	17	p	526	22235650	13237	0.0055
S13 Shef7	17	q	25270411	81048659	32602	0.0071
S13 Shef7	18	p	11543	15402408	10584	0.0081
S13 Shef7	18	q	18529353	66903336	32645	0.0134
S13 Shef7	18	q	66905522	78015057	8147	-0.0372

S13 Shef7	19	p	90910	24596750	11441	0.0064
S13 Shef7	19	q	27747993	59097854	18354	0.0036
S13 Shef7	20	p	61305	26305579	20211	0.0137
S13 Shef7	20	q	29420352	62956153	22679	-0.0086
S13 Shef7	21	q	14345669	48096957	24583	-0.0075
S13 Shef7	22	q	16052528	47198055	20744	0.0048
S13 Shef7	22	q	47200140	51234455	3204	-0.0697
S13 Shef7	X	p	168477	25698948	16035	0.028
S13 Shef7	X	p	25699213	58561930	17999	-0.016
S13 Shef7	X	q	61728829	86713893	12002	-0.0256
S13 Shef7	X	q	86716634	139920048	29703	0.0296
S13 Shef7	X	q	139920866	155233846	9432	-0.0383
S13 Shef7	Y	p	179542	6101425	834	-1.0689
S13 Shef7	Y	p	6107901	10076242	1911	-1.4365
S13 Shef7	Y	q	13134531	59018259	6034	-1.4698
S14 shef3 ss3	1	p	61735	62633483	36513	0.0077
S14 shef3 ss3	1	p	62633581	65821164	2365	0.0929
S14 shef3 ss3	1	p	65822382	121482979	35771	-0.0097
S14 shef3 ss3	1	q	143982530	176969316	18396	-0.0141
S14 shef3 ss3	1	q	176969318	177195050	179	-0.6023
S14 shef3 ss3	1	q	177195555	249224388	49608	-0.0153
S14 shef3 ss3	2	p	12784	89131067	60431	-0.0157
S14 shef3 ss3	2	q	95327887	212546612	70751	-0.0075
S14 shef3 ss3	2	q	212546762	243089456	19731	-0.0404
S14 shef3 ss3	3	p	60345	8639568	7704	-0.0788
S14 shef3 ss3	3	p	8640049	58993627	32513	-0.0107

S14 shef3 ss3	3	p	58998899	61438944	2365	-0.0824
S14 shef3 ss3	3	p	61439273	66834221	4430	0.0692
S14 shef3 ss3	3	p	66834402	90502862	14568	-0.0112
S14 shef3 ss3	3	q	93519478	197896118	64380	-0.0192
S14 shef3 ss3	4	p	68821	49658612	32066	-0.033
S14 shef3 ss3	4	q	52685699	157437060	64117	-0.0136
S14 shef3 ss3	4	q	157441782	191027923	22226	-0.0378
S14 shef3 ss3	5	p	15532	46389273	31096	-0.0321
S14 shef3 ss3	5	q	49432831	180790320	82883	-0.0168
S14 shef3 ss3	6	p	149661	58774716	39810	-0.0234
S14 shef3 ss3	6	q	61886440	93348991	19998	-0.0343
S14 shef3 ss3	6	q	93349763	115285383	13815	0.0131
S14 shef3 ss3	6	q	115290340	171051005	37329	-0.0279
S14 shef3 ss3	7	p	43259	58019983	41049	-0.0213
S14 shef3 ss3	7	q	61063974	133785177	41833	-0.0011
S14 shef3 ss3	7	q	133785195	133790443	25	-0.8468
S14 shef3 ss3	7	q	133795634	159127004	16423	-0.0197
S14 shef3 ss3	8	p	31254	43824048	33696	-0.0263
S14 shef3 ss3	8	q	46847534	134555877	55102	-0.026
S14 shef3 ss3	8	q	134556145	137677896	2489	-0.1079
S14 shef3 ss3	8	q	137681619	137863880	178	-0.5969
S14 shef3 ss3	8	q	137863926	146298155	5054	-0.0689
S14 shef3 ss3	9	p	46587	39110886	31539	-0.0137
S14 shef3 ss3	9	q	71006575	141091394	48558	-0.0038
S14 shef3 ss3	10	p	72759	39076221	29502	-0.0291
S14 shef3 ss3	10	q	42433540	135506704	62552	-0.0133

S14 shef3 ss3	11	p	198510	51564427	34743	-0.0182
S14 shef3 ss3	11	q	54701645	134944770	53268	-0.0088
S14 shef3 ss3	12	p	150442	34854498	23917	-0.0026
S14 shef3 ss3	12	q	37857751	133778189	61976	-0.0113
S14 shef3 ss3	13	q	19026949	115108397	65125	-0.0082
S14 shef3 ss3	14	q	20425911	106008645	54802	-0.0119
S14 shef3 ss3	15	q	22752520	102469040	52124	-0.0089
S14 shef3 ss3	16	p	60777	31960151	20324	-0.015
S14 shef3 ss3	16	q	46463782	79735653	22953	6.00E-04
S14 shef3 ss3	16	q	79738467	90287535	9090	-0.0462
S14 shef3 ss3	17	p	526	18355392	11623	-0.0204
S14 shef3 ss3	17	p	18358946	18469197	30	-0.933
S14 shef3 ss3	17	p	18474646	22235650	1584	0.0325
S14 shef3 ss3	17	q	25270411	81048659	32602	-0.0018
S14 shef3 ss3	18	p	11543	15402408	10584	-0.0097
S14 shef3 ss3	18	q	18529353	66956043	32667	-0.0075
S14 shef3 ss3	18	q	66956704	78015057	8125	-0.0661
S14 shef3 ss3	19	p	90910	24596750	11441	-0.0057
S14 shef3 ss3	19	q	27747993	59097854	18354	-0.0074
S14 shef3 ss3	20	p	61305	26305579	20211	-0.0081
S14 shef3 ss3	20	q	29420352	62956153	22679	-0.0236
S14 shef3 ss3	21	q	14345669	48096957	24583	-0.0237
S14 shef3 ss3	22	q	16052528	47190853	20735	0.003
S14 shef3 ss3	22	q	47195178	51234455	3213	-0.0647
S14 shef3 ss3	X	p	168477	2694240	427	-0.0035
S14 shef3 ss3	X	p	2703391	58561930	33607	-0.5735

S14 shef3 ss3	X	q	61728829	88438261	13134	-0.568
S14 shef3 ss3	X	q	88438577	92463547	1700	-0.4259
S14 shef3 ss3	X	q	92463817	155233846	36303	-0.5615
S14 shef3 ss3	Y	p	179542	10076242	2745	0.0449
S14 shef3 ss3	Y	q	13134531	59018259	6034	0.0141
S15 h9 ss3	1	p	61735	8268178	4171	-0.0532
S15 h9 ss3	1	p	8268715	62439280	32183	0.0145
S15 h9 ss3	1	p	62439298	65821164	2524	0.1137
S15 h9 ss3	1	p	65822382	98041720	21126	-6.00E-04
S15 h9 ss3	1	p	98042396	107462842	5672	-0.0656
S15 h9 ss3	1	p	107466730	121482979	8973	0.0057
S15 h9 ss3	1	q	143982530	145414869	321	0.1918
S15 h9 ss3	1	q	145416056	186216663	24439	-0.0039
S15 h9 ss3	1	q	186216752	195814883	6396	-0.0771
S15 h9 ss3	1	q	195815418	249224388	37027	-0.0054
S15 h9 ss3	2	p	12784	23496096	17460	-0.0491
S15 h9 ss3	2	p	23500116	89131067	42971	-2.00E-04
S15 h9 ss3	2	q	95327887	165838420	42118	-0.0182
S15 h9 ss3	2	q	165843619	176453185	6814	0.0496
S15 h9 ss3	2	q	176461268	188266070	7337	-0.0202
S15 h9 ss3	2	q	188267714	189645193	761	0.1452
S15 h9 ss3	2	q	189645263	201567968	6986	-0.0285
S15 h9 ss3	2	q	201568369	211116383	5760	0.0375
S15 h9 ss3	2	q	211117705	243089456	20706	-0.0469
S15 h9 ss3	3	p	60345	8430919	7519	-0.0716
S15 h9 ss3	3	p	8438577	61461798	35087	-0.001

S15 h9 ss3	3	p	61463002	66752561	4327	0.0946
S15 h9 ss3	3	p	66753918	90502862	14647	-0.0209
S15 h9 ss3	3	q	93519478	108692050	8840	-0.0454
S15 h9 ss3	3	q	108692989	150909068	26251	0.0044
S15 h9 ss3	3	q	150909795	197896118	29289	-0.026
S15 h9 ss3	4	p	68821	31664561	21155	-0.0257
S15 h9 ss3	4	p	31665279	37537922	3701	-0.1074
S15 h9 ss3	4	p	37538743	49658612	7210	-0.0275
S15 h9 ss3	4	q	52685699	154648152	62158	-0.0121
S15 h9 ss3	4	q	154648419	191027923	24185	-0.0397
S15 h9 ss3	5	p	15532	31236979	21818	-0.0335
S15 h9 ss3	5	p	31237336	37555233	4244	0.0268
S15 h9 ss3	5	p	37555317	46389273	5034	-0.0625
S15 h9 ss3	5	q	49432831	68321745	12251	-0.0365
S15 h9 ss3	5	q	68326128	95380339	14922	0.0207
S15 h9 ss3	5	q	95383161	105911408	6232	-0.0823
S15 h9 ss3	5	q	105914094	145301189	25466	-0.0044
S15 h9 ss3	5	q	145301292	147481385	1506	0.1156
S15 h9 ss3	5	q	147483886	180790320	22506	-0.0281
S15 h9 ss3	6	p	149661	32452929	23247	-0.0182
S15 h9 ss3	6	p	32454275	32570365	33	0.808
S15 h9 ss3	6	p	32573771	46142552	8738	0.0171
S15 h9 ss3	6	p	46143710	58774716	7792	-0.038
S15 h9 ss3	6	q	61886440	93242592	19941	-0.0348
S15 h9 ss3	6	q	93243328	115191582	13840	0.0194
S15 h9 ss3	6	q	115195456	171051005	37361	-0.029

S15 h9 ss3	7	p	43259	58019983	41049	-0.0079
S15 h9 ss3	7	q	61063974	62716091	322	0.2887
S15 h9 ss3	7	q	62720216	69146789	3135	-0.0417
S15 h9 ss3	7	q	69147138	84532205	8750	0.0486
S15 h9 ss3	7	q	84532870	121217566	21917	-0.0241
S15 h9 ss3	7	q	121217742	122513683	746	0.1402
S15 h9 ss3	7	q	122515657	144339619	13798	0.0101
S15 h9 ss3	7	q	144345460	159127004	9613	-0.0339
S15 h9 ss3	8	p	31254	12247486	11342	-0.0311
S15 h9 ss3	8	p	12260927	12552851	96	0.4955
S15 h9 ss3	8	p	12554743	43824048	22258	-0.0182
S15 h9 ss3	8	q	46847534	67868944	12720	-0.0407
S15 h9 ss3	8	q	67871165	75048599	4691	0.0546
S15 h9 ss3	8	q	75048636	134555877	37691	-0.0292
S15 h9 ss3	8	q	134556145	146298155	7721	-0.0906
S15 h9 ss3	9	p	46587	39110886	31539	-0.0056
S15 h9 ss3	9	q	71006575	141091394	48558	-0.0032
S15 h9 ss3	10	p	72759	11767337	10131	-0.0597
S15 h9 ss3	10	p	11767378	39076221	19371	-0.0128
S15 h9 ss3	10	q	42433540	106416916	41235	-0.0011
S15 h9 ss3	10	q	106417796	135506704	21317	-0.034
S15 h9 ss3	11	p	198510	51564427	34743	-0.0113
S15 h9 ss3	11	q	54701645	134944770	53268	-0.0103
S15 h9 ss3	12	p	150442	34854498	23917	0.0039
S15 h9 ss3	12	q	37857751	90022376	32142	0.0038
S15 h9 ss3	12	q	90023225	93693393	2415	-0.1105

S15 h9 ss3	12	q	93693461	133778189	27419	-0.0214
S15 h9 ss3	13	q	19026949	50174203	22225	-0.0017
S15 h9 ss3	13	q	50179207	61458197	6558	0.0588
S15 h9 ss3	13	q	61458472	93319943	20461	-0.043
S15 h9 ss3	13	q	93322734	101732563	5727	0.0337
S15 h9 ss3	13	q	101733417	115108397	10154	-0.0376
S15 h9 ss3	14	q	20425911	63555491	27241	-0.004
S15 h9 ss3	14	q	63556035	63793983	176	0.3408
S15 h9 ss3	14	q	63796190	65984383	1436	0.0251
S15 h9 ss3	14	q	65988935	66407140	245	0.3586
S15 h9 ss3	14	q	66407339	106008645	25704	-0.0191
S15 h9 ss3	15	q	22752520	85819321	39340	0.0114
S15 h9 ss3	15	q	85823405	102469040	12784	-0.0412
S15 h9 ss3	16	p	60777	19944363	13716	-0.0055
S15 h9 ss3	16	p	19945650	19967362	30	-1.318
S15 h9 ss3	16	p	19970254	31960151	6578	-0.0202
S15 h9 ss3	16	q	46463782	90287535	32043	-0.0184
S15 h9 ss3	17	p	526	6863347	4005	-0.0172
S15 h9 ss3	17	p	6866025	7583290	315	-0.5163
S15 h9 ss3	17	p	7583705	22235650	8917	-0.0065
S15 h9 ss3	17	q	25270411	49738192	13448	0.0156
S15 h9 ss3	17	q	49738719	54159044	3189	-0.0587
S15 h9 ss3	17	q	54160914	54172591	27	-1.0755
S15 h9 ss3	17	q	54172823	66885372	7336	0.0188
S15 h9 ss3	17	q	66885468	81048659	8602	-0.0359
S15 h9 ss3	18	p	11543	15402408	10584	0.0078

S15 h9 ss3	18	q	18529353	31731071	8252	-0.0055
S15 h9 ss3	18	q	31731862	35310968	2328	0.0796
S15 h9 ss3	18	q	35313501	66742417	21898	-0.0209
S15 h9 ss3	18	q	66742755	66753326	41	-0.6038
S15 h9 ss3	18	q	66757696	78015057	8273	-0.0724
S15 h9 ss3	19	p	90910	24596750	11441	4.00E-04
S15 h9 ss3	19	q	27747993	59097854	18354	-0.0132
S15 h9 ss3	20	p	61305	26305579	20211	0.0582
S15 h9 ss3	20	q	29420352	53267111	15660	0.0716
S15 h9 ss3	20	q	53268004	62956153	7019	0.0239
S15 h9 ss3	21	q	14345669	48096957	24583	-0.0331
S15 h9 ss3	22	q	16052528	47289785	20807	0.0094
S15 h9 ss3	22	q	47289798	51234455	3141	-0.0774
S15 h9 ss3	X	p	168477	23367895	14606	-0.0224
S15 h9 ss3	X	p	23368052	25689542	1422	0.0657
S15 h9 ss3	X	p	25689928	58561930	18006	-0.0519
S15 h9 ss3	X	q	61728829	90727403	14086	-0.0352
S15 h9 ss3	X	q	90729580	112846794	11935	0.0134
S15 h9 ss3	X	q	112847007	115362234	1384	0.1283
S15 h9 ss3	X	q	115362823	155233846	23732	-0.0506
S15 h9 ss3	Y	p	179542	6117910	839	-1.0418
S15 h9 ss3	Y	p	6120432	10076242	1906	-1.4341
S15 h9 ss3	Y	q	13134531	59018259	6034	-1.4811
S16 502 EC	1	p	61735	65848034	38897	-0.0415
S16 502 EC	1	p	65848036	121482979	35752	-0.0946
S16 502 EC	1	q	143982530	249224388	68183	-0.0998

S16 502 EC	2	p	12784	89131067	60431	0.0915
S16 502 EC	2	q	95327887	243089456	90482	0.0844
S16 502 EC	3	p	60345	61449577	42597	-0.0925
S16 502 EC	3	p	61450007	90502862	18983	-0.0252
S16 502 EC	3	q	93519478	98937784	2876	-0.039
S16 502 EC	3	q	98944458	98949056	27	-1.3055
S16 502 EC	3	q	98949069	197896118	61477	-0.0041
S16 502 EC	4	p	68821	10417792	6052	-0.0741
S16 502 EC	4	p	10418078	49658612	26014	-0.139
S16 502 EC	4	q	52685699	58512554	4000	-0.0831
S16 502 EC	4	q	58515319	118113290	36404	-0.14
S16 502 EC	4	q	118117532	191027923	45939	-0.2595
S16 502 EC	5	p	15532	10866460	8939	-0.1218
S16 502 EC	5	p	10870591	16103315	3635	-0.2705
S16 502 EC	5	p	16108127	46389273	18522	-0.1214
S16 502 EC	5	q	49432831	180790320	82883	-0.1172
S16 502 EC	6	p	149661	58774716	39810	0.0011
S16 502 EC	6	q	61886440	171051005	71142	-0.0126
S16 502 EC	7	p	43259	58019983	41049	0.2422
S16 502 EC	7	q	61063974	65241440	1327	0.3156
S16 502 EC	7	q	65243596	72234742	4081	0.2152
S16 502 EC	7	q	72235882	77009056	1526	0.3949
S16 502 EC	7	q	77009894	109430050	20017	0.2468
S16 502 EC	7	q	109433747	109453910	39	-0.5532
S16 502 EC	7	q	109453982	159127004	31291	0.2483
S16 502 EC	8	p	31254	2294789	1749	0.2333

S16 502 EC	8	p	2294906	24974476	20206	0.1503
S16 502 EC	8	p	24974522	24984333	25	-0.8566
S16 502 EC	8	p	24991104	43824048	11716	0.1668
S16 502 EC	8	q	46847534	144530651	62248	0.1539
S16 502 EC	8	q	144532005	146298155	575	-0.0024
S16 502 EC	9	p	46587	39110886	31539	0.1973
S16 502 EC	9	q	71006575	127126278	40855	0.2789
S16 502 EC	9	q	127126951	141091394	7703	-0.0844
S16 502 EC	10	p	72759	39076221	29502	-0.2311
S16 502 EC	10	q	42433540	134945927	62305	-0.2267
S16 502 EC	10	q	134956549	135506704	247	-0.543
S16 502 EC	11	p	198510	51564427	34743	-0.1073
S16 502 EC	11	q	54701645	62255196	4447	-0.1114
S16 502 EC	11	q	62255294	82925395	11590	0.0212
S16 502 EC	11	q	82926091	134944770	37231	-0.2373
S16 502 EC	12	p	150442	34854498	23917	0.2929
S16 502 EC	12	q	37857751	133778189	61976	0.0035
S16 502 EC	13	q	19026949	115108397	65125	-5.00E-04
S16 502 EC	14	q	20425911	52980341	20225	0.0403
S16 502 EC	14	q	52980625	53702651	362	0.2791
S16 502 EC	14	q	53703744	106008645	34215	0.0527
S16 502 EC	15	q	22752520	102469040	52124	-0.1025
S16 502 EC	16	p	60777	31960151	20324	0.0851
S16 502 EC	16	q	46463782	90287535	32043	-0.1064
S16 502 EC	17	p	526	18319862	11622	0.1164
S16 502 EC	17	p	18355392	18469197	31	-0.7554

S16 502 EC	17	p	18474646	22235650	1584	0.1278
S16 502 EC	17	q	25270411	44163098	10595	0.1173
S16 502 EC	17	q	44165803	44394414	135	0.7645
S16 502 EC	17	q	44394420	81048659	21872	0.1096
S16 502 EC	18	p	11543	15402408	10584	-0.0958
S16 502 EC	18	q	18529353	78015057	40792	-0.1183
S16 502 EC	19	p	90910	20138859	8898	-0.0842
S16 502 EC	19	p	20139389	24596750	2543	0.0178
S16 502 EC	19	q	27747993	59097854	18354	-0.0731
S16 502 EC	20	p	61305	14771472	11208	0.1219
S16 502 EC	20	p	14781684	14823905	69	-0.5736
S16 502 EC	20	p	14830476	26305579	8934	0.1128
S16 502 EC	20	q	29420352	62956153	22679	0.0989
S16 502 EC	21	q	14345669	48096957	24583	-0.1167
S16 502 EC	22	q	16052528	51234455	23948	0.0046
S16 502 EC	X	p	168477	2739032	460	0.18
S16 502 EC	X	p	2739612	58561930	33574	-0.0604
S16 502 EC	X	q	61728829	155233846	51137	-0.3452
S16 502 EC	Y	p	179542	10076242	2745	-0.3173
S16 502 EC	Y	q	13134531	59018259	6034	-0.3646
S17 564 EC	1	p	61735	45569347	25419	0.0539
S17 564 EC	1	p	45572966	107372400	40176	-0.0919
S17 564 EC	1	p	107374308	111377368	2678	0.0605
S17 564 EC	1	p	111377565	111388226	38	-0.6263
S17 564 EC	1	p	111392067	121482979	6338	0.046
S17 564 EC	1	q	143982530	225670344	51477	0.0404

S17 564 EC	1	q	225673293	249224388	16706	-0.0875
S17 564 EC	2	p	12784	89131067	60431	0.0391
S17 564 EC	2	q	95327887	243089456	90482	0.0376
S17 564 EC	3	p	60345	4561649	4134	0.0187
S17 564 EC	3	p	4562667	87820622	56052	-0.0944
S17 564 EC	3	p	87821109	90502862	1394	0.063
S17 564 EC	3	q	93519478	197896118	64380	0.0371
S17 564 EC	4	p	68821	49089374	32023	-0.1024
S17 564 EC	4	p	49093786	49658612	43	0.3924
S17 564 EC	4	q	52685699	191027923	86343	-0.0985
S17 564 EC	5	p	15532	46389273	31096	-0.0963
S17 564 EC	5	q	49432831	177198449	80849	-0.1013
S17 564 EC	5	q	177227551	180790320	2034	-0.2353
S17 564 EC	6	p	149661	58774716	39810	-0.0981
S17 564 EC	6	q	61886440	65690306	2021	-0.098
S17 564 EC	6	q	65690847	66271030	397	0.2254
S17 564 EC	6	q	66271154	98403999	20719	-0.1014
S17 564 EC	6	q	98404412	143724518	28667	-0.2641
S17 564 EC	6	q	143724552	144659091	700	-0.0915
S17 564 EC	6	q	144659946	159592777	10186	-0.2652
S17 564 EC	6	q	159594164	161128632	1157	-0.0793
S17 564 EC	6	q	161128947	162800237	1386	-0.2479
S17 564 EC	6	q	162800915	171051005	5909	-0.1013
S17 564 EC	7	p	43259	30271449	22094	0.2654
S17 564 EC	7	p	30271698	48493397	12478	0.1652
S17 564 EC	7	p	48495720	58019983	6477	0.3611

S17 564 EC	7	q	61063974	62077305	78	0.4738
S17 564 EC	7	q	62077637	76430850	6516	0.0862
S17 564 EC	7	q	76432322	76607295	146	0.5108
S17 564 EC	7	q	76709612	159127004	51541	0.0421
S17 564 EC	8	p	31254	12239835	11311	0.0385
S17 564 EC	8	p	12240162	12558929	130	0.4145
S17 564 EC	8	p	12559835	34980347	17368	0.0481
S17 564 EC	8	p	34980598	43824048	4887	0.1695
S17 564 EC	8	q	46847534	103112417	34180	0.1556
S17 564 EC	8	q	103113668	146298155	28643	0.0308
S17 564 EC	9	p	46587	11941913	11340	0.0304
S17 564 EC	9	p	11945913	12249026	265	-0.2737
S17 564 EC	9	p	12249277	39110886	19934	0.0474
S17 564 EC	9	q	71006575	141091394	48558	-0.0731
S17 564 EC	10	p	72759	39076221	29502	-0.0789
S17 564 EC	10	q	42433540	70424973	17765	-0.2422
S17 564 EC	10	q	70426333	135506704	44787	-0.0903
S17 564 EC	11	p	198510	5789741	3325	0.0456
S17 564 EC	11	p	5789869	5809139	27	-0.9575
S17 564 EC	11	p	5809338	21956669	12309	0.0501
S17 564 EC	11	p	21968275	21989330	22	-0.9165
S17 564 EC	11	p	21992527	51564427	19060	0.0379
S17 564 EC	11	q	54701645	134944770	53268	0.0409
S17 564 EC	12	p	150442	13616901	9252	0.6697
S17 564 EC	12	p	13617712	31099949	12303	0.6164
S17 564 EC	12	p	31102896	34854498	2362	0.6876

S17 564 EC	12	q	37857751	44662601	4141	0.151
S17 564 EC	12	q	44669483	133778189	57835	0.0438
S17 564 EC	13	q	19026949	110810769	62529	-0.0817
S17 564 EC	13	q	110811451	115108397	2596	-0.2437
S17 564 EC	14	q	20425911	106008645	54802	0.0433
S17 564 EC	15	q	22752520	43395847	13027	-0.0771
S17 564 EC	15	q	43397963	102469040	39097	0.043
S17 564 EC	16	p	60777	31960151	20324	-0.083
S17 564 EC	16	q	46463782	90287535	32043	-0.0851
S17 564 EC	17	p	526	4839104	2606	-0.0873
S17 564 EC	17	p	4839149	12129766	4885	0.1744
S17 564 EC	17	p	12129863	18355392	4132	0.2771
S17 564 EC	17	p	18358946	18465221	29	-0.5479
S17 564 EC	17	p	18469197	21369629	1267	0.3015
S17 564 EC	17	p	21371532	22235650	318	0.1041
S17 564 EC	17	q	25270411	54932066	17286	0.0458
S17 564 EC	17	q	54938001	81048659	15316	0.167
S17 564 EC	18	p	11543	15402408	10584	-0.0838
S17 564 EC	18	q	18529353	78015057	40792	-0.0976
S17 564 EC	19	p	90910	20256366	8971	-0.0603
S17 564 EC	19	p	20256839	24596750	2470	0.0162
S17 564 EC	19	q	27747993	59097854	18354	-0.0626
S17 564 EC	20	p	61305	1627994	1251	0.07
S17 564 EC	20	p	1630499	26305579	18960	-0.0786
S17 564 EC	20	q	29420352	62956153	22679	0.0379
S17 564 EC	21	q	14345669	48096957	24583	0.1559

S17 564 EC	22	q	16052528	25664832	5592	-0.0325
S17 564 EC	22	q	25666235	25922342	296	0.4306
S17 564 EC	22	q	25922347	51234455	18060	-0.0869
S17 564 EC	X	p	168477	2739032	460	0.0745
S17 564 EC	X	p	2739612	32116483	19777	-0.2571
S17 564 EC	X	p	32117083	32848166	603	-0.4893
S17 564 EC	X	p	32849295	58561930	13194	-0.2371
S17 564 EC	X	q	61728829	155233846	51137	-0.252
S17 564 EC	Y	p	179542	10076242	2745	-0.3288
S17 564 EC	Y	q	13134531	26150538	5289	-0.3883
S17 564 EC	Y	q	26152733	59018259	745	-0.2237
S18 1740 EC	1	p	61735	65821164	38878	0.0576
S18 1740 EC	1	p	65822382	74382510	5570	0.01
S18 1740 EC	1	p	74385848	99291831	16208	0.1152
S18 1740 EC	1	p	99293779	121482979	13993	0.0073
S18 1740 EC	1	q	143982530	152757690	1924	0.027
S18 1740 EC	1	q	152759678	152768259	33	-0.6611
S18 1740 EC	1	q	152768281	192379022	26787	-0.0653
S18 1740 EC	1	q	192384797	200853111	5696	-0.0099
S18 1740 EC	1	q	200853433	213155566	8396	0.0868
S18 1740 EC	1	q	213155948	236187118	15895	-0.144
S18 1740 EC	1	q	236187308	243006007	5523	-0.0506
S18 1740 EC	1	q	243007084	249224388	3929	0.1069
S18 1740 EC	2	p	12784	15038607	11407	-0.0325
S18 1740 EC	2	p	15041886	18391396	2514	0.1882
S18 1740 EC	2	p	18391550	30053342	7301	0.0123

S18 1740 EC	2	p	30056441	64476261	23808	-0.0509
S18 1740 EC	2	p	64478287	89131067	15401	0.019
S18 1740 EC	2	q	95327887	105008404	5131	0.1796
S18 1740 EC	2	q	105008715	111597991	3766	0.2602
S18 1740 EC	2	q	111598255	121936827	6384	0.0425
S18 1740 EC	2	q	121939973	133529769	6934	0.2409
S18 1740 EC	2	q	133530104	148555609	9475	0.1557
S18 1740 EC	2	q	148561845	179553867	19271	0.0205
S18 1740 EC	2	q	179554305	243089456	39521	-0.0589
S18 1740 EC	3	p	60345	4658341	4225	0.0292
S18 1740 EC	3	p	4658835	60626006	37644	-0.0514
S18 1740 EC	3	p	60627290	74743456	11048	0.0545
S18 1740 EC	3	p	74743990	90502862	8663	0.145
S18 1740 EC	3	q	93519478	197896118	64380	0.0094
S18 1740 EC	4	p	68821	49658612	32066	-0.0621
S18 1740 EC	4	q	52685699	151868941	60458	-0.0099
S18 1740 EC	4	q	151876309	156319821	2810	0.1241
S18 1740 EC	4	q	156320512	164480801	5341	-0.0228
S18 1740 EC	4	q	164487375	188791456	16261	-0.0928
S18 1740 EC	4	q	188793575	191027923	1473	-0.0134
S18 1740 EC	5	p	15532	46389273	31096	-0.0573
S18 1740 EC	5	q	49432831	180790320	82883	-0.0716
S18 1740 EC	6	p	149661	58774716	39810	0.0168
S18 1740 EC	6	q	61886440	69653759	4824	-0.0121
S18 1740 EC	6	q	69656950	171051005	66318	-0.0651
S18 1740 EC	7	p	43259	58019983	41049	0.0948

S18 1740 EC	7	q	61063974	159127004	58281	0.0605
S18 1740 EC	8	p	31254	12247486	11342	-0.0214
S18 1740 EC	8	p	12260927	12558929	99	0.4012
S18 1740 EC	8	p	12559835	24974476	10514	-0.0248
S18 1740 EC	8	p	24974522	24984333	25	-0.9722
S18 1740 EC	8	p	24991104	43824048	11716	-0.0208
S18 1740 EC	8	q	46847534	59928264	8000	0.0075
S18 1740 EC	8	q	59929562	146298155	54823	-0.0362
S18 1740 EC	9	p	46587	39110886	31539	-0.0398
S18 1740 EC	9	q	71006575	74138579	2495	-0.0482
S18 1740 EC	9	q	74138754	76720154	1683	0.0675
S18 1740 EC	9	q	76721039	79331065	2012	-0.0503
S18 1740 EC	9	q	79331368	87303958	5769	0.0732
S18 1740 EC	9	q	87311385	101004955	9265	0.2673
S18 1740 EC	9	q	101005776	108873997	5674	0.1334
S18 1740 EC	9	q	108875720	114982703	4895	0.0386
S18 1740 EC	9	q	114988001	141091394	16765	-0.0623
S18 1740 EC	10	p	72759	39076221	29502	-0.051
S18 1740 EC	10	q	42433540	135506704	62552	-0.0505
S18 1740 EC	11	p	198510	51564427	34743	-0.0079
S18 1740 EC	11	q	54701645	77936166	12179	-0.0089
S18 1740 EC	11	q	77936188	134944770	41089	-0.0631
S18 1740 EC	12	p	150442	4016201	2621	0.234
S18 1740 EC	12	p	4016642	7734978	2683	0.3536
S18 1740 EC	12	p	7735225	9149303	714	0.6269
S18 1740 EC	12	p	9153146	10894663	1106	0.4666

S18 1740 EC	12	p	10895716	15611518	3464	0.2502
S18 1740 EC	12	p	15611862	21689370	3884	0.4604
S18 1740 EC	12	p	21691046	34854498	9445	0.2728
S18 1740 EC	12	q	37857751	63918671	15638	-0.0268
S18 1740 EC	12	q	63919488	69065175	3386	0.0727
S18 1740 EC	12	q	69069778	72388272	2248	0.1955
S18 1740 EC	12	q	72388839	114686840	27429	-0.0376
S18 1740 EC	12	q	114687347	133778189	13275	0.0136
S18 1740 EC	13	q	19026949	115108397	65125	-0.0526
S18 1740 EC	14	q	20425911	106008645	54802	0.012
S18 1740 EC	15	q	22752520	34619314	7433	-0.0105
S18 1740 EC	15	q	34619956	35389267	458	0.1645
S18 1740 EC	15	q	35391526	87280117	32655	-0.0367
S18 1740 EC	15	q	87283179	102469040	11578	0.0206
S18 1740 EC	16	p	60777	31960151	20324	-0.0363
S18 1740 EC	16	q	46463782	90287535	32043	-0.0579
S18 1740 EC	17	p	526	22235650	13237	0.0263
S18 1740 EC	17	q	25270411	81048659	32602	0.0184
S18 1740 EC	18	p	11543	15402408	10584	-0.047
S18 1740 EC	18	q	18529353	78015057	40792	-0.0572
S18 1740 EC	19	p	90910	20256366	8971	0.0284
S18 1740 EC	19	p	20256839	24596750	2470	0.0945
S18 1740 EC	19	q	27747993	59097854	18354	-0.0454
S18 1740 EC	20	p	61305	26305579	20211	0.0228
S18 1740 EC	20	q	29420352	62956153	22679	-2.00E-04
S18 1740 EC	21	q	14345669	48096957	24583	0.0903

S18 1740 EC	22	q	16052528	51234455	23948	0.0021
S18 1740 EC	X	p	168477	2694240	427	-0.0547
S18 1740 EC	X	p	2703391	47879024	29090	-0.5159
S18 1740 EC	X	p	47880157	48001295	81	0.1771
S18 1740 EC	X	p	48003925	58561930	4436	-0.4907
S18 1740 EC	X	q	61728829	88407111	13118	-0.5026
S18 1740 EC	X	q	88417808	92466625	1724	-0.3563
S18 1740 EC	X	q	92468672	152698755	35306	-0.5141
S18 1740 EC	X	q	152699682	155233846	989	-0.4105
S18 1740 EC	Y	p	179542	10076242	2745	-0.0653
S18 1740 EC	Y	q	13134531	23649664	4706	-0.0891
S18 1740 EC	Y	q	23652318	59018259	1328	0.0095
S19 1838 EC	1	p	61735	111377565	68274	0.0198
S19 1838 EC	1	p	111377568	111388226	37	-0.9194
S19 1838 EC	1	p	111392067	121482979	6338	0.008
S19 1838 EC	1	q	143982530	152759678	1925	0.0508
S19 1838 EC	1	q	152761923	152768259	32	-1.4588
S19 1838 EC	1	q	152768281	249224388	66226	0.0048
S19 1838 EC	2	p	12784	89131067	60431	0.0144
S19 1838 EC	2	q	95327887	243089456	90482	0.0087
S19 1838 EC	3	p	60345	48487353	34294	-0.0624
S19 1838 EC	3	p	48487386	90502862	27286	0.0219
S19 1838 EC	3	q	93519478	197896118	64380	0.0095
S19 1838 EC	4	p	68821	49658612	32066	-0.0767
S19 1838 EC	4	q	52685699	191027923	86343	-0.0761
S19 1838 EC	5	p	15532	46389273	31096	-0.0666

S19 1838 EC	5	q	49432831	180790320	82883	-0.0761
S19 1838 EC	6	p	149661	58774716	39810	0.0017
S19 1838 EC	6	q	61886440	171051005	71142	-0.0668
S19 1838 EC	7	p	43259	58019983	41049	0.0968
S19 1838 EC	7	q	61063974	133785177	41833	0.1125
S19 1838 EC	7	q	133785195	133795634	26	-0.7564
S19 1838 EC	7	q	133795665	159127004	16422	0.1116
S19 1838 EC	8	p	31254	24974476	21955	0.0161
S19 1838 EC	8	p	24974522	24984333	25	-0.9624
S19 1838 EC	8	p	24991104	43824048	11716	0.0213
S19 1838 EC	8	q	46847534	146298155	62823	0.0161
S19 1838 EC	9	p	46587	39110886	31539	0.0219
S19 1838 EC	9	q	71006575	141091394	48558	-0.0522
S19 1838 EC	10	p	72759	39076221	29502	-0.0682
S19 1838 EC	10	q	42433540	135506704	62552	-0.0634
S19 1838 EC	11	p	198510	51564427	34743	-0.058
S19 1838 EC	11	q	54701645	75451343	10856	-0.0415
S19 1838 EC	11	q	75451411	134944770	42412	-0.1586
S19 1838 EC	12	p	150442	34854498	23917	0.3869
S19 1838 EC	12	q	37857751	133778189	61976	0.0075
S19 1838 EC	13	q	19026949	81241718	41808	-0.0097
S19 1838 EC	13	q	81243383	115108397	23317	0.0693
S19 1838 EC	14	q	20425911	106008645	54802	-0.0722
S19 1838 EC	15	q	22752520	33012814	6062	0.0953
S19 1838 EC	15	q	33015169	38054187	3691	0.0216
S19 1838 EC	15	q	38055027	102469040	42371	-0.0668

S19 1838 EC	16	p	60777	31960151	20324	-0.0609
S19 1838 EC	16	q	46463782	90287535	32043	-0.0553
S19 1838 EC	17	p	526	16576239	10659	-0.0258
S19 1838 EC	17	p	16577056	22235650	2578	0.1303
S19 1838 EC	17	q	25270411	81048659	32602	0.0339
S19 1838 EC	18	p	11543	15402408	10584	-0.0688
S19 1838 EC	18	q	18529353	78015057	40792	-0.0709
S19 1838 EC	19	p	90910	24596750	11441	0.0575
S19 1838 EC	19	q	27747993	36452818	5947	0.0276
S19 1838 EC	19	q	36455960	59097854	12407	-0.0392
S19 1838 EC	20	p	61305	26305579	20211	-0.0447
S19 1838 EC	20	q	29420352	62916068	22674	-0.052
S19 1838 EC	20	q	62954695	62956153	5	-1.4734
S19 1838 EC	21	q	14345669	48096957	24583	0.0685
S19 1838 EC	22	q	16052528	20122920	2130	0.1044
S19 1838 EC	22	q	20123475	23263274	1879	0.222
S19 1838 EC	22	q	23266118	51234455	19939	-0.0497
S19 1838 EC	X	p	168477	2693624	426	0.0435
S19 1838 EC	X	p	2694240	58561930	33608	-0.4808
S19 1838 EC	X	q	61728829	155233846	51137	-0.4682
S19 1838 EC	Y	p	179542	10076242	2745	-0.0237
S19 1838 EC	Y	q	13134531	59018259	6034	-0.0345
S20 864EC	1	p	61735	7974075	4011	-0.0489
S20 864EC	1	p	7975789	25583291	10348	0.0062
S20 864EC	1	p	25583341	25646986	30	-0.9148
S20 864EC	1	p	25661501	121482979	60260	0.0126

S20 864EC	1	q	143982530	152757690	1924	0.0566
S20 864EC	1	q	152759678	152778861	46	-0.6115
S20 864EC	1	q	152780139	249224388	66213	5.00E-04
S20 864EC	2	p	12784	23777734	17704	-0.1151
S20 864EC	2	p	23778829	89131067	42727	-0.0724
S20 864EC	2	q	95327887	101994236	3222	-0.068
S20 864EC	2	q	101994731	105962117	2507	0.2048
S20 864EC	2	q	105964684	237932661	81753	-0.0812
S20 864EC	2	q	237932954	243089456	3000	-0.1476
S20 864EC	3	p	60345	61234123	42459	-0.015
S20 864EC	3	p	61234832	90502862	19121	0.0144
S20 864EC	3	q	93519478	129762859	21803	-0.0028
S20 864EC	3	q	129763698	129806236	55	-0.8213
S20 864EC	3	q	129806924	197896118	42522	0.0036
S20 864EC	4	p	68821	49658612	32066	-0.1872
S20 864EC	4	q	52685699	191027923	86343	-0.1796
S20 864EC	5	p	15532	15329150	12095	0.1123
S20 864EC	5	p	15329178	46389273	19001	0.1653
S20 864EC	5	q	49432831	180790320	82883	-6.00E-04
S20 864EC	6	p	149661	1381035	1170	-0.1714
S20 864EC	6	p	1385038	35658894	23875	-0.0669
S20 864EC	6	p	35661743	54071413	12258	0.0814
S20 864EC	6	p	54071470	58774716	2507	-0.0624
S20 864EC	6	q	61886440	163942208	66069	-0.072
S20 864EC	6	q	163943266	171051005	5073	-0.1201
S20 864EC	7	p	43259	12291093	8010	0.0629

S20 864EC	7	p	12300822	14425270	1775	0.1588
S20 864EC	7	p	14425492	20444081	4923	0.0815
S20 864EC	7	p	20446793	58019983	26341	0.1424
S20 864EC	7	q	61063974	81737960	10258	0.1705
S20 864EC	7	q	81738903	84529804	1946	0.2445
S20 864EC	7	q	84531007	159127004	46077	0.1488
S20 864EC	8	p	31254	13516633	12369	0.1498
S20 864EC	8	p	13516921	24974443	9585	0.1885
S20 864EC	8	p	24974476	24984333	26	-1.1279
S20 864EC	8	p	24991104	43824048	11716	0.1906
S20 864EC	8	q	46847534	133969484	54504	0.1796
S20 864EC	8	q	133972411	146298155	8319	0.1283
S20 864EC	9	p	46587	39110886	31539	-0.0291
S20 864EC	9	q	71006575	75888737	3682	0.1376
S20 864EC	9	q	75889144	76029826	60	-0.3997
S20 864EC	9	q	76032184	136553499	42681	0.1135
S20 864EC	9	q	136557606	141091394	2135	0.0425
S20 864EC	10	p	72759	14302149	12238	-0.1949
S20 864EC	10	p	14302239	39076221	17264	-0.1482
S20 864EC	10	q	42433540	135506704	62552	-0.1786
S20 864EC	11	p	198510	51564427	34743	-0.0874
S20 864EC	11	q	54701645	134944770	53268	-0.0901
S20 864EC	12	p	150442	7994787	5435	0.5436
S20 864EC	12	p	8004425	8134669	62	1.1569
S20 864EC	12	p	8134717	33294660	17584	0.5604
S20 864EC	12	p	33299791	33307374	28	-0.3448

S20 864EC	12	p	33308360	34854498	808	0.5365
S20 864EC	12	q	37857751	133778189	61976	0.0503
S20 864EC	13	q	19026949	115108397	65125	0.1129
S20 864EC	14	q	20425911	106008645	54802	0.077
S20 864EC	15	q	22752520	102469040	52124	-0.1762
S20 864EC	16	p	60777	31960151	20324	0.0653
S20 864EC	16	q	46463782	85428804	28851	0.0618
S20 864EC	16	q	85428915	90287535	3192	-0.0063
S20 864EC	17	p	526	22235650	13237	0.1065
S20 864EC	17	q	25270411	81048659	32602	0.0941
S20 864EC	18	p	11543	15402408	10584	-0.1108
S20 864EC	18	q	18529353	72256692	36750	-0.1127
S20 864EC	18	q	72260399	78015057	4042	-0.1612
S20 864EC	19	p	90910	19617134	8598	-0.0793
S20 864EC	19	p	19620857	24596750	2843	0.0336
S20 864EC	19	q	27747993	35849322	5630	-0.0891
S20 864EC	19	q	35849493	35861848	23	-1.0536
S20 864EC	19	q	35866744	59097854	12701	-0.0494
S20 864EC	20	p	61305	26305579	20211	0.0847
S20 864EC	20	q	29420352	59157134	20481	0.0762
S20 864EC	20	q	59158923	62956153	2198	0.0152
S20 864EC	21	q	14345669	39072984	17793	0.1274
S20 864EC	21	q	39075749	48096957	6790	0.0755
S20 864EC	22	q	16052528	46103356	20106	0.0465
S20 864EC	22	q	46105089	51234455	3842	-0.0243
S20 864EC	X	p	168477	2694240	427	9.00E-04

S20 864EC	X	p	2703391	46999829	28617	-0.5482
S20 864EC	X	p	47001791	53763852	2827	-0.2375
S20 864EC	X	p	53763875	58561930	2163	-0.1304
S20 864EC	X	q	61728829	67091901	2212	-0.2125
S20 864EC	X	q	67092455	77535996	4479	-0.1325
S20 864EC	X	q	77538042	88438577	6444	-0.2114
S20 864EC	X	q	88440097	115930727	14627	-0.1584
S20 864EC	X	q	115939213	116056435	76	0.4607
S20 864EC	X	q	116056465	155233846	23299	-0.1867
S20 864EC	Y	p	179542	10076242	2745	-0.1151
S20 864EC	Y	q	13134531	59018259	6034	-0.1268
S21 1545EC	1	p	61735	121482979	74649	0.0586
S21 1545EC	1	q	143982530	249224388	68183	-0.0304
S21 1545EC	2	p	12784	89131067	60431	0.0252
S21 1545EC	2	q	95327887	130781750	21071	0.038
S21 1545EC	2	q	130783696	131244208	126	0.3551
S21 1545EC	2	q	131284168	132219998	339	-0.3263
S21 1545EC	2	q	132221406	211458170	48449	0.0161
S21 1545EC	2	q	211462522	243089456	20497	-0.0137
S21 1545EC	3	p	60345	128189	59	0.3188
S21 1545EC	3	p	134991	1576168	1287	-0.0894
S21 1545EC	3	p	1576194	1720759	173	0.2814
S21 1545EC	3	p	1721148	90502862	60061	-0.0612
S21 1545EC	3	q	93519478	129766586	21806	-0.057
S21 1545EC	3	q	129775858	129806924	53	-0.7724
S21 1545EC	3	q	129806998	197896118	42521	-0.0416

S21 1545EC	4	p	68821	49658612	32066	-0.1221
S21 1545EC	4	q	52685699	191027923	86343	-0.1195
S21 1545EC	5	p	15532	46389273	31096	0.0376
S21 1545EC	5	q	49432831	180790320	82883	0.0277
S21 1545EC	6	p	149661	58774716	39810	0.0085
S21 1545EC	6	q	61886440	171051005	71142	-0.0161
S21 1545EC	7	p	43259	2419292	791	0.1267
S21 1545EC	7	p	2421453	58019983	40258	0.2836
S21 1545EC	7	q	61063974	76277909	6508	0.0945
S21 1545EC	7	q	76282709	141763124	40595	0.0188
S21 1545EC	7	q	141769643	141792107	42	-1.1137
S21 1545EC	7	q	141795281	159127004	11136	0.0262
S21 1545EC	8	p	31254	43824048	33696	0.1392
S21 1545EC	8	q	46847534	146298155	62823	0.1396
S21 1545EC	9	p	46587	39110886	31539	-0.0227
S21 1545EC	9	q	71006575	141091394	48558	-0.0134
S21 1545EC	10	p	72759	39076221	29502	-0.0234
S21 1545EC	10	q	42433540	47068227	2552	-0.009
S21 1545EC	10	q	47068243	47743501	269	0.3573
S21 1545EC	10	q	47743516	135506704	59731	-0.0293
S21 1545EC	11	p	198510	51564427	34743	0.0723
S21 1545EC	11	q	54701645	57350784	1563	0.0656
S21 1545EC	11	q	57352562	66916397	4835	-0.0273
S21 1545EC	11	q	66918629	70642820	1853	-0.1853
S21 1545EC	11	q	70644885	85754562	9613	-0.033
S21 1545EC	11	q	85754641	134944770	35404	-0.2149

S21 1545EC	12	p	150442	20201839	13526	0.6283
S21 1545EC	12	p	20204226	26013149	4171	0.341
S21 1545EC	12	p	26014375	34854498	6220	0.1168
S21 1545EC	12	q	37857751	133778189	61976	0.0532
S21 1545EC	13	q	19026949	115108397	65125	-0.0243
S21 1545EC	14	q	20425911	106008645	54802	-0.034
S21 1545EC	15	q	22752520	76891241	34220	-0.0398
S21 1545EC	15	q	76891511	76895775	21	-0.9812
S21 1545EC	15	q	76902179	102469040	17883	-0.0513
S21 1545EC	16	p	60777	31960151	20324	0.0347
S21 1545EC	16	q	46463782	90287535	32043	-0.1352
S21 1545EC	17	p	526	22235650	13237	0.07
S21 1545EC	17	q	25270411	81048659	32602	0.0602
S21 1545EC	18	p	11543	1902886	1326	-0.06
S21 1545EC	18	p	1905358	1980787	97	-0.5772
S21 1545EC	18	p	1986214	15402408	9161	-0.0652
S21 1545EC	18	q	18529353	78015057	40792	-0.0755
S21 1545EC	19	p	90910	20835890	9278	-0.0382
S21 1545EC	19	p	20836742	20985425	90	0.4655
S21 1545EC	19	p	20985936	24596750	2073	0.0453
S21 1545EC	19	q	27747993	44383800	10026	-0.0246
S21 1545EC	19	q	44394480	52506384	4136	-0.11
S21 1545EC	19	q	52507538	59097854	4192	-0.0346
S21 1545EC	20	p	61305	26305579	20211	0.0105
S21 1545EC	20	q	29420352	29881042	95	0.029
S21 1545EC	20	q	29882151	31459721	753	0.5078

S21 1545EC	20	q	31461082	62956153	21831	-0.0586
S21 1545EC	21	q	14345669	48096957	24583	-0.0284
S21 1545EC	22	q	16052528	25664832	5592	-0.0019
S21 1545EC	22	q	25666235	25910844	275	0.3872
S21 1545EC	22	q	25910894	51234455	18081	-0.031
S21 1545EC	X	p	168477	2694240	427	-0.0796
S21 1545EC	X	p	2703391	58561930	33607	-0.5
S21 1545EC	X	q	61728829	155233846	51137	-0.5057
S21 1545EC	Y	p	179542	10076242	2745	-0.3963
S21 1545EC	Y	q	13134531	25748291	5133	-0.4449
S21 1545EC	Y	q	25782121	28203848	642	-0.8314
S21 1545EC	Y	q	28204148	59018259	259	-0.3245
S22 1999EC	1	p	61735	5561184	2454	0.0615
S22 1999EC	1	p	5561511	25583341	11906	0.1558
S22 1999EC	1	p	25593128	25646986	29	-0.7978
S22 1999EC	1	p	25661501	44757199	10557	0.1799
S22 1999EC	1	p	44758178	45216284	295	-0.0702
S22 1999EC	1	p	45216645	53900967	4614	0.2163
S22 1999EC	1	p	53901001	62083652	6222	0.1555
S22 1999EC	1	p	62083960	66225517	3082	0.2667
S22 1999EC	1	p	66225999	101749974	23131	0.1683
S22 1999EC	1	p	101750248	107468819	3389	0.095
S22 1999EC	1	p	107470789	121482979	8970	0.1855
S22 1999EC	1	q	143982530	152606047	1785	0.2672
S22 1999EC	1	q	152606110	183193145	20954	0.1695
S22 1999EC	1	q	183193520	183683459	347	0.408

S22	1999EC	1	q	183683749	199770642	10592	0.1323
S22	1999EC	1	q	199772234	206945311	4603	0.2025
S22	1999EC	1	q	206946458	209639383	2069	0.0989
S22	1999EC	1	q	209639617	248737447	27652	0.1743
S22	1999EC	1	q	248749194	248795289	31	-0.5246
S22	1999EC	1	q	248795852	249224388	150	0.3109
S22	1999EC	2	p	12784	23615247	17553	0.0953
S22	1999EC	2	p	23615897	49093975	17312	0.1588
S22	1999EC	2	p	49094809	53586206	3333	0.0859
S22	1999EC	2	p	53590450	56598309	2147	0.1935
S22	1999EC	2	p	56598827	60494383	2671	0.0879
S22	1999EC	2	p	60494589	89131067	17415	0.156
S22	1999EC	2	q	95327887	137240069	24669	0.1198
S22	1999EC	2	q	137240304	148254622	6807	0.0671
S22	1999EC	2	q	148258988	211472763	38519	0.1298
S22	1999EC	2	q	211476389	215049390	2390	0.0387
S22	1999EC	2	q	215054758	234860165	12810	0.1153
S22	1999EC	2	q	234860612	243089456	5287	0.0569
S22	1999EC	3	p	60345	8450884	7531	-0.1457
S22	1999EC	3	p	8451805	90502862	54049	-0.1045
S22	1999EC	3	q	93519478	102037685	4660	-0.1099
S22	1999EC	3	q	102043743	108400650	3969	-0.1748
S22	1999EC	3	q	108401146	142564507	21071	-0.0937
S22	1999EC	3	q	142566337	143002916	376	0.1064
S22	1999EC	3	q	143004353	181381406	23770	-0.1168
S22	1999EC	3	q	181386463	187472812	3837	-0.0289

S22 1999EC	3	q	187473274	195997787	5747	-0.1254
S22 1999EC	3	q	195999993	197896118	950	-0.0218
S22 1999EC	4	p	68821	32031296	21369	-0.1288
S22 1999EC	4	p	32036925	37224082	3234	-0.1966
S22 1999EC	4	p	37224201	42762117	3665	-0.0731
S22 1999EC	4	p	42764949	47769025	2945	-0.1753
S22 1999EC	4	p	47772548	49658612	853	0.0117
S22 1999EC	4	q	52685699	55494179	1844	-0.1108
S22 1999EC	4	q	55495793	58702467	2304	-0.0195
S22 1999EC	4	q	58703619	68116485	5751	-0.1679
S22 1999EC	4	q	68116539	118450631	30698	-0.114
S22 1999EC	4	q	118452268	130344612	7012	-0.066
S22 1999EC	4	q	130346136	163574042	20459	-0.1217
S22 1999EC	4	q	163582000	188501025	16634	-0.5056
S22 1999EC	4	q	188502133	191027923	1641	-0.4037
S22 1999EC	5	p	15532	16123847	12587	-0.1453
S22 1999EC	5	p	16124245	46389273	18509	-0.0961
S22 1999EC	5	q	49432831	68240676	12191	-0.1031
S22 1999EC	5	q	68241637	90794973	12543	-0.0503
S22 1999EC	5	q	90800869	130261147	25102	-0.1142
S22 1999EC	5	q	130265110	142133585	6919	-0.0609
S22 1999EC	5	q	142133942	144924760	1889	-0.1716
S22 1999EC	5	q	144928580	151156794	4289	-0.0716
S22 1999EC	5	q	151164767	175266894	17105	-0.1315
S22 1999EC	5	q	175267342	180790320	2845	-0.0756
S22 1999EC	6	p	149661	19636433	15432	-0.0706

S22 1999EC	6	p	19636541	39302887	12127	-0.0257
S22 1999EC	6	p	39303966	58774716	12251	-0.0755
S22 1999EC	6	q	61886440	73524889	7346	-0.1271
S22 1999EC	6	q	73526074	76680208	1977	0.0044
S22 1999EC	6	q	76680290	107169467	19374	-0.0676
S22 1999EC	6	q	107169608	112738769	3495	-0.0023
S22 1999EC	6	q	112740967	164390919	34260	-0.0765
S22 1999EC	6	q	164392414	171051005	4690	-0.1192
S22 1999EC	7	p	43259	12326930	8035	0.1362
S22 1999EC	7	p	12328765	14412633	1736	0.2665
S22 1999EC	7	p	14412895	19094320	3775	0.1447
S22 1999EC	7	p	19094495	31147397	9082	0.2027
S22 1999EC	7	p	31147906	53833104	15909	0.1566
S22 1999EC	7	p	53833331	58019983	2512	0.2343
S22 1999EC	7	q	61063974	67048258	2058	0.2827
S22 1999EC	7	q	67048671	159127004	56223	0.1923
S22 1999EC	8	p	31254	2294789	1749	0.2097
S22 1999EC	8	p	2294906	8585184	6748	0.1124
S22 1999EC	8	p	8585835	43824048	25199	0.1814
S22 1999EC	8	q	46847534	110125762	38513	0.1873
S22 1999EC	8	q	110125878	114392517	2308	0.1207
S22 1999EC	8	q	114393054	115718780	805	0.316
S22 1999EC	8	q	115720979	131588565	11023	0.1823
S22 1999EC	8	q	131594918	146298155	10174	0.1058
S22 1999EC	9	p	46587	39110886	31539	-0.0981
S22 1999EC	9	q	71006575	136447810	46353	-0.067

S22 1999EC	9	q	136458404	141091394	2205	-0.1577
S22 1999EC	10	p	72759	39076221	29502	-0.1006
S22 1999EC	10	q	42433540	49287603	3149	-0.0621
S22 1999EC	10	q	49292463	59194778	6981	-0.1429
S22 1999EC	10	q	59195435	93283423	23335	-0.1036
S22 1999EC	10	q	93283640	106212334	7627	-0.0397
S22 1999EC	10	q	106214081	111383172	3880	-0.1668
S22 1999EC	10	q	111384666	135506704	17580	-0.1115
S22 1999EC	11	p	198510	51564427	34743	-0.1094
S22 1999EC	11	q	54701645	79489932	13408	-0.0883
S22 1999EC	11	q	79490072	134944770	39860	-0.1183
S22 1999EC	12	p	150442	18935189	12645	0.8072
S22 1999EC	12	p	18935494	20344297	957	0.9436
S22 1999EC	12	p	20345281	34854498	10315	0.8263
S22 1999EC	12	q	37857751	58773781	12246	0.1848
S22 1999EC	12	q	58775715	62220727	2214	0.086
S22 1999EC	12	q	62221143	81644757	12750	0.2036
S22 1999EC	12	q	81645182	93693393	7347	0.1131
S22 1999EC	12	q	93693461	126203752	21504	0.171
S22 1999EC	12	q	126203814	133778189	5915	0.1238
S22 1999EC	13	q	19026949	22061125	1755	-0.003
S22 1999EC	13	q	22064086	74339104	35216	-0.0985
S22 1999EC	13	q	74339344	115108397	28154	-0.1254
S22 1999EC	14	q	20425911	25101706	2589	-0.0557
S22 1999EC	14	q	25103946	26617436	1108	-0.1968
S22 1999EC	14	q	26621962	82199134	35655	-0.0869

S22 1999EC	14	q	82202541	106008645	15450	-0.1223
S22 1999EC	15	q	22752520	85539712	39191	-0.0523
S22 1999EC	15	q	85541520	102469040	12933	-0.0923
S22 1999EC	16	p	60777	31960151	20324	-0.0781
S22 1999EC	16	q	46463782	48662675	1011	-0.0065
S22 1999EC	16	q	48666500	66394390	12688	-0.125
S22 1999EC	16	q	66398525	77322757	6385	-0.0729
S22 1999EC	16	q	77323172	90287535	11959	-0.1604
S22 1999EC	17	p	526	22235650	13237	0.1886
S22 1999EC	17	q	25270411	31475497	3069	0.2098
S22 1999EC	17	q	31478459	32911191	1409	0.0819
S22 1999EC	17	q	32911831	49926090	9139	0.2029
S22 1999EC	17	q	49927790	54699046	3446	0.0921
S22 1999EC	17	q	54699096	66885372	6937	0.2079
S22 1999EC	17	q	66885468	81048659	8602	0.1253
S22 1999EC	18	p	11543	15402408	10584	-0.0879
S22 1999EC	18	q	18529353	21303692	1428	-0.0384
S22 1999EC	18	q	21304029	28926384	4971	-0.1374
S22 1999EC	18	q	28927184	29937076	719	0.0259
S22 1999EC	18	q	29940450	78015057	33674	-0.1283
S22 1999EC	19	p	90910	20049050	8829	-0.0837
S22 1999EC	19	p	20049586	24596750	2612	0.0906
S22 1999EC	19	q	27747993	33620897	4112	-0.1382
S22 1999EC	19	q	33624878	43286764	5326	-0.0468
S22 1999EC	19	q	43287456	43519362	112	-0.4812
S22 1999EC	19	q	43524295	59097854	8804	-0.0465

S22 1999EC	20	p	61305	10796644	8339	0.0092
S22 1999EC	20	p	10797035	24876583	11203	-0.0316
S22 1999EC	20	p	24876728	26305579	669	0.1074
S22 1999EC	20	q	29420352	37905178	4335	0.0187
S22 1999EC	20	q	37905674	41402981	2721	-0.0946
S22 1999EC	20	q	41403661	58900315	13219	-0.0242
S22 1999EC	20	q	58900580	62956153	2404	-0.1034
S22 1999EC	21	q	14345669	41496851	20007	0.1508
S22 1999EC	21	q	41498026	48096957	4576	0.1041
S22 1999EC	22	q	16052528	46043202	20073	-0.0863
S22 1999EC	22	q	46044787	51234455	3875	-0.1886
S22 1999EC	X	p	168477	2694240	427	0.181
S22 1999EC	X	p	2703391	16404476	10042	-0.2179
S22 1999EC	X	p	16404877	25692600	5565	-0.1612
S22 1999EC	X	p	25698948	46093582	12546	-0.2292
S22 1999EC	X	p	46093706	58561930	5454	-0.1399
S22 1999EC	X	q	61728829	67130358	2227	-0.2347
S22 1999EC	X	q	67130648	77635135	4498	-0.1315
S22 1999EC	X	q	77636214	95714580	10039	-0.2394
S22 1999EC	X	q	95715042	140805635	25402	-0.185
S22 1999EC	X	q	140807611	146177397	3816	-0.2914
S22 1999EC	X	q	146177413	154929498	5118	-0.1951
S22 1999EC	X	q	154963439	155233846	37	0.275
S22 1999EC	Y	p	179542	10076242	2745	-0.1566
S22 1999EC	Y	q	13134531	59018259	6034	-0.1841
S23 2131EC	1	p	61735	25583341	14360	0.0259

S23 2131EC	1	p	25593128	25646986	29	-0.8761
S23 2131EC	1	p	25661501	66225517	24770	0.0484
S23 2131EC	1	p	66225999	121482979	35490	0.0147
S23 2131EC	1	q	143982530	152757690	1924	0.0775
S23 2131EC	1	q	152759678	152778861	46	-0.5792
S23 2131EC	1	q	152780139	249224388	66213	0.0235
S23 2131EC	2	p	12784	89131067	60431	0.023
S23 2131EC	2	q	95327887	243089456	90482	0.0193
S23 2131EC	3	p	60345	2595938	2236	-0.2387
S23 2131EC	3	p	2596432	53023211	33897	-0.0899
S23 2131EC	3	p	53028375	53039263	39	-1.2751
S23 2131EC	3	p	53039279	90502862	25408	-0.0876
S23 2131EC	3	q	93519478	197896118	64380	-0.0733
S23 2131EC	4	p	68821	49658612	32066	-0.1104
S23 2131EC	4	q	52685699	161041304	66243	-0.1111
S23 2131EC	4	q	161052774	161070789	40	-0.9017
S23 2131EC	4	q	161083870	191027923	20060	-0.1025
S23 2131EC	5	p	15532	46389273	31096	-0.01
S23 2131EC	5	q	49432831	180790320	82883	-0.0159
S23 2131EC	6	p	149661	58774716	39810	0.0284
S23 2131EC	6	q	61886440	67008286	2914	0.0043
S23 2131EC	6	q	67008811	67048629	56	-0.5638
S23 2131EC	6	q	67049406	162513347	62032	0.0235
S23 2131EC	6	q	162513436	171051005	6140	-0.1672
S23 2131EC	7	p	43259	58019983	41049	0.1617
S23 2131EC	7	q	61063974	110956260	28005	0.1537

S23 2131EC	7	q	110963735	111122305	122	-0.5007
S23 2131EC	7	q	111132722	159127004	30154	0.1507
S23 2131EC	8	p	31254	43824048	33696	0.1089
S23 2131EC	8	q	46847534	146298155	62823	0.1053
S23 2131EC	9	p	46587	39110886	31539	-0.027
S23 2131EC	9	q	71006575	141091394	48558	-0.0839
S23 2131EC	10	p	72759	39076221	29502	-0.093
S23 2131EC	10	q	42433540	135506704	62552	-0.1012
S23 2131EC	11	p	198510	51564427	34743	-0.1103
S23 2131EC	11	q	54701645	79453054	13387	-0.0874
S23 2131EC	11	q	79453396	134944770	39881	-0.117
S23 2131EC	12	p	150442	30235581	20768	0.42
S23 2131EC	12	p	30239267	30243689	24	-0.3361
S23 2131EC	12	p	30243722	34854498	3125	0.447
S23 2131EC	12	q	37857751	133778189	61976	0.0274
S23 2131EC	13	q	19026949	115108397	65125	0.0165
S23 2131EC	14	q	20425911	106008645	54802	-0.0203
S23 2131EC	15	q	22752520	76869929	34218	0.037
S23 2131EC	15	q	76878362	76895775	23	-0.8908
S23 2131EC	15	q	76902179	102469040	17883	0.0197
S23 2131EC	16	p	60777	31960151	20324	-0.0966
S23 2131EC	16	q	46463782	90287535	32043	-0.0972
S23 2131EC	17	p	526	22235650	13237	0.053
S23 2131EC	17	q	25270411	61439265	20717	0.0416
S23 2131EC	17	q	61444885	81048659	11885	0.1382
S23 2131EC	18	p	11543	15402408	10584	-0.1034

S23	2131EC	18	q	18529353	78015057	40792	-0.1138
S23	2131EC	19	p	90910	19526732	8548	0.027
S23	2131EC	19	p	19527312	24596750	2893	0.1003
S23	2131EC	19	q	27747993	43290773	9442	0.0172
S23	2131EC	19	q	43294390	43542831	120	-0.6384
S23	2131EC	19	q	43549014	59097854	8792	-0.039
S23	2131EC	20	p	61305	26305579	20211	0.0273
S23	2131EC	20	q	29420352	62956153	22679	0.0279
S23	2131EC	21	q	14345669	48096957	24583	0.1178
S23	2131EC	22	q	16052528	51234455	23948	-0.0686
S23	2131EC	X	p	168477	2694240	427	0.061
S23	2131EC	X	p	2703391	45818033	28027	-0.4332
S23	2131EC	X	p	45818140	58561930	5580	-0.3862
S23	2131EC	X	q	61728829	154929498	51100	-0.4254
S23	2131EC	X	q	154963439	155233846	37	0.0591
S23	2131EC	Y	p	179542	10076242	2745	-0.1003
S23	2131EC	Y	q	13134531	59018259	6034	-0.1128
S24	28EC	1	p	61735	121482979	74649	0.0307
S24	28EC	1	q	143982530	249224388	68183	0.0213
S24	28EC	2	p	12784	89131067	60431	0.0208
S24	28EC	2	q	95327887	243089456	90482	0.025
S24	28EC	3	p	60345	90502862	61580	0.0223
S24	28EC	3	q	93519478	129762859	21803	0.0175
S24	28EC	3	q	129763698	129806236	55	-0.7044
S24	28EC	3	q	129806924	148962992	12013	0.0273
S24	28EC	3	q	148963395	148968121	25	-0.7279

S24 28EC	3	q	148971080	197896118	30484	0.0099
S24 28EC	4	p	68821	45017576	29692	-0.1201
S24 28EC	4	p	45020694	49658612	2374	0.1257
S24 28EC	4	q	52685699	53228771	315	0.2134
S24 28EC	4	q	53233385	60084935	4665	0.6092
S24 28EC	4	q	60094895	70148877	5958	-0.1206
S24 28EC	4	q	70149157	70236151	42	-0.7314
S24 28EC	4	q	70239096	191027923	75363	-0.1142
S24 28EC	5	p	15532	46389273	31096	-0.1108
S24 28EC	5	q	49432831	180790320	82883	-0.1144
S24 28EC	6	p	149661	32454523	23249	0.0104
S24 28EC	6	p	32456683	32570365	31	0.7202
S24 28EC	6	p	32573771	58774716	16530	0.0271
S24 28EC	6	q	61886440	171051005	71142	0.0131
S24 28EC	7	p	43259	58019983	41049	0.1342
S24 28EC	7	q	61063974	159127004	58281	0.1452
S24 28EC	8	p	31254	41924275	32871	-0.1192
S24 28EC	8	p	41927963	43824048	825	0.0314
S24 28EC	8	q	46847534	146298155	62823	0.0264
S24 28EC	9	p	46587	39110886	31539	0.1339
S24 28EC	9	q	71006575	95182624	17578	0.1485
S24 28EC	9	q	95193616	141091394	30980	-0.0912
S24 28EC	10	p	72759	39076221	29502	-0.1118
S24 28EC	10	q	42433540	117789456	49436	-0.0976
S24 28EC	10	q	117789795	135506704	13116	0.1415
S24 28EC	11	p	198510	18940870	12888	0.034

S24 28EC	11	p	18949072	18961402	48	-0.5861
S24 28EC	11	p	18961597	51564427	21807	0.0247
S24 28EC	11	q	54701645	78086945	12265	0.0309
S24 28EC	11	q	78088405	134944770	41003	-0.0053
S24 28EC	12	p	150442	33299791	23082	0.4131
S24 28EC	12	p	33301406	33307374	27	-0.6657
S24 28EC	12	p	33308360	34854498	808	0.441
S24 28EC	12	q	37857751	133778189	61976	0.0239
S24 28EC	13	q	19026949	115108397	65125	-0.1066
S24 28EC	14	q	20425911	106008645	54802	0.0186
S24 28EC	15	q	22752520	102469040	52124	0.0233
S24 28EC	16	p	60777	31960151	20324	-0.0982
S24 28EC	16	q	46463782	90287535	32043	-0.1008
S24 28EC	17	p	526	18355392	11623	0.04
S24 28EC	17	p	18358946	18465221	29	-0.8302
S24 28EC	17	p	18469197	22235650	1585	0.0385
S24 28EC	17	q	25270411	81048659	32602	0.0374
S24 28EC	18	p	11543	12740615	9256	-0.2688
S24 28EC	18	p	12740643	15402408	1328	0.0066
S24 28EC	18	q	18529353	77173979	40382	-0.1045
S24 28EC	18	q	77181989	78015057	410	0.1023
S24 28EC	19	p	90910	24596750	11441	0.0625
S24 28EC	19	q	27747993	34635750	4820	0.0452
S24 28EC	19	q	34637568	59097854	13534	-0.0772
S24 28EC	20	p	61305	26305579	20211	-0.0897
S24 28EC	20	q	29420352	62956153	22679	-0.0964

S24 28EC	21	q	14345669	48096957	24583	0.0281
S24 28EC	22	q	16052528	46043202	20073	0.0544
S24 28EC	22	q	46044787	51234455	3875	-0.0962
S24 28EC	X	p	168477	2694240	427	0.2225
S24 28EC	X	p	2703391	6449766	2933	-0.4216
S24 28EC	X	p	6455163	6461450	24	-1.2711
S24 28EC	X	p	6467914	8135657	1207	-2.1369
S24 28EC	X	p	8141089	58561930	29443	-0.4129
S24 28EC	X	q	61728829	154963439	51101	-0.4048
S24 28EC	X	q	154964055	155233846	36	0.1644
S24 28EC	Y	p	179542	10076242	2745	0.3218
S24 28EC	Y	q	13134531	25355963	5078	0.3159
S24 28EC	Y	q	25403867	28463158	785	0.6486
S24 28EC	Y	q	28463526	59018259	171	0.2575
S25 1017EC	1	p	61735	101203341	61952	0.1588
S25 1017EC	1	p	101203765	106310661	2967	0.0768
S25 1017EC	1	p	106313476	121482979	9730	0.1515
S25 1017EC	1	q	143982530	249224388	68183	0.1328
S25 1017EC	2	p	12784	17171039	12962	0.0748
S25 1017EC	2	p	17171290	89131067	47469	0.1175
S25 1017EC	2	q	95327887	211649947	70128	0.1058
S25 1017EC	2	q	211650309	215047196	2256	0.0198
S25 1017EC	2	q	215049390	243089456	18098	0.0936
S25 1017EC	3	p	60345	90502862	61580	0.1345
S25 1017EC	3	q	93519478	150683143	34950	0.1433
S25 1017EC	3	q	150683270	178576306	17265	0.1058

S25 1017EC	3	q	178576471	187534915	5516	0.1697
S25 1017EC	3	q	187537757	197896118	6649	0.1059
S25 1017EC	4	p	68821	32361203	21606	-0.1615
S25 1017EC	4	p	32362043	37223576	2996	-0.256
S25 1017EC	4	p	37224082	49658612	7464	-0.1819
S25 1017EC	4	q	52685699	191027923	86343	-0.1768
S25 1017EC	5	p	15532	46389273	31096	-0.1696
S25 1017EC	5	q	49432831	180790320	82883	-0.1557
S25 1017EC	6	p	149661	14792349	11822	0.0854
S25 1017EC	6	p	14792476	58774716	27988	0.1304
S25 1017EC	6	q	61886440	73805091	7547	0.078
S25 1017EC	6	q	73806100	132328949	36789	0.1305
S25 1017EC	6	q	132329218	171051005	26806	0.0962
S25 1017EC	7	p	43259	58019983	41049	-0.165
S25 1017EC	7	q	61063974	67077181	2078	-0.0867
S25 1017EC	7	q	67077406	159127004	56203	-0.1597
S25 1017EC	8	p	31254	15723221	14501	0.2937
S25 1017EC	8	p	15724921	43824048	19195	0.3448
S25 1017EC	8	q	46847534	114413411	40832	0.3409
S25 1017EC	8	q	114413678	115929717	942	0.473
S25 1017EC	8	q	115929843	134475910	13238	0.3353
S25 1017EC	8	q	134479388	146298155	7811	0.2959
S25 1017EC	9	p	46587	12165758	11533	-0.174
S25 1017EC	9	p	12165791	12243401	68	-0.6906
S25 1017EC	9	p	12248419	39110886	19938	-0.1449
S25 1017EC	9	q	71006575	141091394	48558	-0.1493

S25 1017EC	10	p	72759	39076221	29502	-0.1691
S25 1017EC	10	q	42433540	135506704	62552	-0.1592
S25 1017EC	11	p	198510	51564427	34743	-0.1586
S25 1017EC	11	q	54701645	79489932	13408	-0.1306
S25 1017EC	11	q	79490072	134944770	39860	-0.1708
S25 1017EC	12	p	150442	2235889	1287	0.7663
S25 1017EC	12	p	2245862	2257920	41	0.044
S25 1017EC	12	p	2258023	34854498	22589	0.7959
S25 1017EC	12	q	37857751	83859925	28580	0.1444
S25 1017EC	12	q	83860006	133778189	33396	0.1174
S25 1017EC	13	q	19026949	115108397	65125	-0.1709
S25 1017EC	14	q	20425911	40793161	12602	-0.1529
S25 1017EC	14	q	40801751	41359987	378	-0.6434
S25 1017EC	14	q	41365648	106008645	41822	-0.1594
S25 1017EC	15	q	22752520	63529126	26481	-0.1924
S25 1017EC	15	q	63532059	102469040	25643	-0.0342
S25 1017EC	16	p	60777	7603667	5168	0.1187
S25 1017EC	16	p	7604466	7702789	132	-0.2475
S25 1017EC	16	p	7702810	31960151	15024	0.1232
S25 1017EC	16	q	46463782	77355291	20128	0.1287
S25 1017EC	16	q	77356174	90287535	11915	0.0681
S25 1017EC	17	p	526	22235650	13237	0.1509
S25 1017EC	17	q	25270411	81048659	32602	0.1497
S25 1017EC	18	p	11543	15402408	10584	-0.1698
S25 1017EC	18	q	18529353	78015057	40792	-0.1726
S25 1017EC	19	p	90910	20256366	8971	-0.1105

S25 1017EC	19	p	20256839	24596750	2470	-0.0195
S25 1017EC	19	q	27747993	59097854	18354	-0.1133
S25 1017EC	20	p	61305	26305579	20211	-0.1005
S25 1017EC	20	q	29420352	62956153	22679	-0.1495
S25 1017EC	21	q	14345669	48096957	24583	0.1271
S25 1017EC	22	q	16052528	46050587	20077	-0.1258
S25 1017EC	22	q	46057366	51234455	3871	-0.2043
S25 1017EC	X	p	168477	58561930	34034	-0.1668
S25 1017EC	X	q	61728829	81485786	8908	-0.1386
S25 1017EC	X	q	81492309	95713395	7855	-0.1999
S25 1017EC	X	q	95714580	140895389	25466	-0.148
S25 1017EC	X	q	140895492	146711244	4200	-0.2208
S25 1017EC	X	q	146711649	155233846	4708	-0.1421
S25 1017EC	Y	p	179542	10076242	2745	-1.1088
S25 1017EC	Y	q	13134531	59018259	6034	-1.2327
S26 1118EC	1	p	61735	85955261	52158	0.0291
S26 1118EC	1	p	85955772	121482979	22491	-0.0057
S26 1118EC	1	q	143982530	249224388	68183	0.0093
S26 1118EC	2	p	12784	89131067	60431	0.0068
S26 1118EC	2	q	95327887	243089456	90482	0.0113
S26 1118EC	3	p	60345	9205904	8221	-0.0613
S26 1118EC	3	p	9208043	46679339	25290	-0.0039
S26 1118EC	3	p	46679675	53313291	2833	0.0914
S26 1118EC	3	p	53317230	89018866	24463	-0.0048
S26 1118EC	3	p	89021806	90502862	773	0.1341
S26 1118EC	3	q	93519478	197896118	64380	0.004

S26 1118EC	4	p	68821	49658612	32066	-0.129
S26 1118EC	4	q	52685699	191027923	86343	-0.1253
S26 1118EC	5	p	15532	46389273	31096	-0.1085
S26 1118EC	5	q	49432831	180790320	82883	-0.1145
S26 1118EC	6	p	149661	254267	123	0.0045
S26 1118EC	6	p	254283	382897	110	-0.5057
S26 1118EC	6	p	384109	58774716	39577	-0.0254
S26 1118EC	6	q	61886440	171051005	71142	-0.0203
S26 1118EC	7	p	43259	58019983	41049	0.2178
S26 1118EC	7	q	61063974	159127004	58281	0.2311
S26 1118EC	8	p	31254	43824048	33696	0.091
S26 1118EC	8	q	46847534	146298155	62823	0.1035
S26 1118EC	9	p	46587	39110886	31539	-0.0075
S26 1118EC	9	q	71006575	141091394	48558	0.0064
S26 1118EC	10	p	72759	39076221	29502	-0.1336
S26 1118EC	10	q	42433540	135506704	62552	-0.1177
S26 1118EC	11	p	198510	51564427	34743	-0.1294
S26 1118EC	11	q	54701645	55376280	251	-0.1695
S26 1118EC	11	q	55376498	55442318	42	-1.5875
S26 1118EC	11	q	55444211	134944770	52975	-0.129
S26 1118EC	12	p	150442	33294660	23081	0.4621
S26 1118EC	12	p	33299791	33307374	28	-1.0071
S26 1118EC	12	p	33308360	34854498	808	0.5081
S26 1118EC	12	q	37857751	133778189	61976	0.1168
S26 1118EC	13	q	19026949	115108397	65125	-6.00E-04
S26 1118EC	14	q	20425911	106008645	54802	-0.1265

S26 1118EC	15	q	22752520	102469040	52124	-0.1261
S26 1118EC	16	p	60777	31960151	20324	0.0043
S26 1118EC	16	q	46463782	77258592	20018	0.0186
S26 1118EC	16	q	77261152	90287535	12025	-0.032
S26 1118EC	17	p	526	18319862	11622	0.0273
S26 1118EC	17	p	18355392	18465221	30	-0.9577
S26 1118EC	17	p	18469197	22235650	1585	0.0533
S26 1118EC	17	q	25270411	81048659	32602	0.0346
S26 1118EC	18	p	11543	15402408	10584	-0.1424
S26 1118EC	18	q	18529353	78015057	40792	-0.1306
S26 1118EC	19	p	90910	24596750	11441	-0.0515
S26 1118EC	19	q	27747993	59097854	18354	-0.0778
S26 1118EC	20	p	61305	2771921	2231	-0.1176
S26 1118EC	20	p	2775332	26305579	17980	0.0048
S26 1118EC	20	q	29420352	62956153	22679	0.0027
S26 1118EC	21	q	14345669	48096957	24583	0.1267
S26 1118EC	22	q	16052528	25684408	5600	0.0679
S26 1118EC	22	q	25684831	25914085	281	0.4382
S26 1118EC	22	q	25914331	51234455	18067	0.0234
S26 1118EC	X	p	168477	2694240	427	0.1072
S26 1118EC	X	p	2703391	46409569	28330	-0.3783
S26 1118EC	X	p	46413528	58561930	5277	-0.318
S26 1118EC	X	q	61728829	155233846	51137	-0.3623
S26 1118EC	Y	p	179542	10076242	2745	0.2377
S26 1118EC	Y	q	13134531	59018259	6034	0.2355
S27 1375EC	1	p	61735	121482979	74649	0.1205

S27	1375EC	1	q	143982530	240803170	62521	0.1026
S27	1375EC	1	q	240803666	247348189	4624	0.3321
S27	1375EC	1	q	247355464	249224388	1038	0.1417
S27	1375EC	2	p	12784	57394902	40901	0.0149
S27	1375EC	2	p	57402044	57445471	69	0.7678
S27	1375EC	2	p	57447398	89131067	19461	0.0225
S27	1375EC	2	q	95327887	153796068	35030	0.0192
S27	1375EC	2	q	153796549	243089456	55452	-0.0873
S27	1375EC	3	p	60345	9072878	8094	-0.0726
S27	1375EC	3	p	9074865	38037877	19943	-0.0129
S27	1375EC	3	p	38039356	90502862	33543	0.0573
S27	1375EC	3	q	93519478	197896118	64380	0.0419
S27	1375EC	4	p	68821	32052675	21381	-0.0717
S27	1375EC	4	p	32054591	49658612	10685	-0.1147
S27	1375EC	4	q	52685699	191027923	86343	-0.0902
S27	1375EC	5	p	15532	46389273	31096	0.1211
S27	1375EC	5	q	49432831	180790320	82883	-0.0183
S27	1375EC	6	p	149661	58774716	39810	-0.0134
S27	1375EC	6	q	61886440	171051005	71142	-0.0183
S27	1375EC	7	p	43259	58019983	41049	0.0527
S27	1375EC	7	q	61063974	159127004	58281	0.0629
S27	1375EC	8	p	31254	43824048	33696	0.0638
S27	1375EC	8	q	46847534	146298155	62823	0.0544
S27	1375EC	9	p	46587	39110886	31539	-0.0259
S27	1375EC	9	q	71006575	141091394	48558	-0.1053
S27	1375EC	10	p	72759	39076221	29502	-0.2265

S27 1375EC	10	q	42433540	69981150	17536	-0.2285
S27 1375EC	10	q	69981436	135506704	45016	-0.1051
S27 1375EC	11	p	198510	51564427	34743	-0.0201
S27 1375EC	11	q	54701645	134944770	53268	-0.0196
S27 1375EC	12	p	150442	34854498	23917	0.57
S27 1375EC	12	q	37857751	133778189	61976	0.09
S27 1375EC	13	q	19026949	69251649	33479	-0.1075
S27 1375EC	13	q	69251980	69268242	25	-0.9811
S27 1375EC	13	q	69269119	115108397	31621	-0.1152
S27 1375EC	14	q	20425911	106008645	54802	-0.104
S27 1375EC	15	q	22752520	76878362	34219	-0.19
S27 1375EC	15	q	76891241	102469040	17905	-0.0976
S27 1375EC	16	p	60777	31960151	20324	0.001
S27 1375EC	16	q	46463782	62198716	10579	0.0076
S27 1375EC	16	q	62205178	90287535	21464	-0.1044
S27 1375EC	17	p	526	22235650	13237	0.1026
S27 1375EC	17	q	25270411	81048659	32602	0.0978
S27 1375EC	18	p	11543	15402408	10584	-0.1115
S27 1375EC	18	q	18529353	78015057	40792	-0.1033
S27 1375EC	19	p	90910	24596750	11441	-0.0291
S27 1375EC	19	q	27747993	59097854	18354	-0.048
S27 1375EC	20	p	61305	26305579	20211	-0.0205
S27 1375EC	20	q	29420352	52644868	15095	-0.0256
S27 1375EC	20	q	52647808	52658651	38	-0.833
S27 1375EC	20	q	52662574	62956153	7546	-0.0388
S27 1375EC	21	q	14345669	48096957	24583	0.0628

S27	1375EC	22	q	16052528	51234455	23948	-0.0946
S27	1375EC	X	p	168477	2694240	427	0.1595
S27	1375EC	X	p	2703391	46805207	28527	-0.2754
S27	1375EC	X	p	46805294	58561930	5080	-0.2289
S27	1375EC	X	q	61728829	155233846	51137	-0.2539
S27	1375EC	Y	p	179542	10076242	2745	0.1632
S27	1375EC	Y	q	13134531	59018259	6034	0.1471
S28	3113EC	1	p	61735	86341	16	-0.6427
S28	3113EC	1	p	98588	43168141	23988	0.1383
S28	3113EC	1	p	43171773	100997095	37809	0.1696
S28	3113EC	1	p	100997544	121482979	12836	0.1316
S28	3113EC	1	q	143982530	152757690	1924	0.2114
S28	3113EC	1	q	152759678	152768259	33	-0.4823
S28	3113EC	1	q	152768281	249224388	66226	0.1368
S28	3113EC	2	p	12784	15828750	12001	0.0893
S28	3113EC	2	p	15831498	89131067	48430	0.1569
S28	3113EC	2	q	95327887	111717678	8996	0.1245
S28	3113EC	2	q	111720884	133548427	13233	-0.1538
S28	3113EC	2	q	133549115	133700309	121	-0.7064
S28	3113EC	2	q	133701284	242733123	67981	-0.1601
S28	3113EC	2	q	242771411	243089456	151	-0.4793
S28	3113EC	3	p	60345	45547745	32707	0.0889
S28	3113EC	3	p	45549463	66745004	14221	0.1262
S28	3113EC	3	p	66746475	90502862	14652	0.0791
S28	3113EC	3	q	93519478	148962992	33871	0.1068
S28	3113EC	3	q	148963395	148968121	25	-0.7766

S28	3113EC	3	q	148971080	197896118	30484	0.0872
S28	3113EC	4	p	68821	49658612	32066	-0.1812
S28	3113EC	4	q	52685699	191027923	86343	-0.1712
S28	3113EC	5	p	15532	46389273	31096	0.1276
S28	3113EC	5	q	49432831	95404843	27187	0.1707
S28	3113EC	5	q	95405538	97043591	1026	0.0579
S28	3113EC	5	q	97045121	97099320	44	-0.7914
S28	3113EC	5	q	97099684	167849331	46094	0.1507
S28	3113EC	5	q	167851168	180790320	8532	0.115
S28	3113EC	6	p	149661	10386902	8402	0.0971
S28	3113EC	6	p	10387991	58774716	31408	-0.1513
S28	3113EC	6	q	61886440	73492028	7322	-0.2118
S28	3113EC	6	q	73493802	132376574	37046	-0.1424
S28	3113EC	6	q	132376802	171051005	26774	-0.1816
S28	3113EC	7	p	43259	11295119	7038	0.1096
S28	3113EC	7	p	11297124	35148425	18292	0.1659
S28	3113EC	7	p	35150097	58019983	15719	0.1305
S28	3113EC	7	q	61063974	81827390	10337	0.1607
S28	3113EC	7	q	81827631	84551527	1886	0.2783
S28	3113EC	7	q	84554451	150086430	40006	0.1559
S28	3113EC	7	q	150088720	159127004	6052	0.1069
S28	3113EC	8	p	31254	43824048	33696	0.117
S28	3113EC	8	q	46847534	114418955	40837	0.1397
S28	3113EC	8	q	114419090	116113355	1067	0.2761
S28	3113EC	8	q	116116608	131188265	10483	0.1378
S28	3113EC	8	q	131193067	146298155	10436	0.0734

S28 3113EC	9	p	46587	12097002	11471	-0.6347
S28 3113EC	9	p	12097677	39110886	20068	-0.1439
S28 3113EC	9	q	71006575	141091394	48558	-0.1449
S28 3113EC	10	p	72759	19869633	16402	-0.1878
S28 3113EC	10	p	19869674	22879987	2039	-0.0694
S28 3113EC	10	p	22880787	39076221	11061	-0.1711
S28 3113EC	10	q	42433540	46931589	2439	-0.141
S28 3113EC	10	q	46932195	47743516	383	0.4053
S28 3113EC	10	q	47771030	68155999	13607	-0.1777
S28 3113EC	10	q	68156539	106416916	24806	-0.1296
S28 3113EC	10	q	106417796	135506704	21317	-0.1765
S28 3113EC	11	p	198510	51564427	34743	-0.1643
S28 3113EC	11	q	54701645	78010544	12227	-0.1305
S28 3113EC	11	q	78010830	134944770	41041	-0.1703
S28 3113EC	12	p	150442	15584624	10573	0.777
S28 3113EC	12	p	15584855	21787253	3976	0.8651
S28 3113EC	12	p	21787605	31113611	7019	0.7834
S28 3113EC	12	p	31115126	34231115	2126	0.8614
S28 3113EC	12	p	34231416	34275794	23	0.0456
S28 3113EC	12	p	34276055	34854498	200	0.7788
S28 3113EC	12	q	37857751	83712975	28484	0.1643
S28 3113EC	12	q	83714883	133778189	33492	0.1278
S28 3113EC	13	q	19026949	48327155	20999	-0.1745
S28 3113EC	13	q	48328647	61896332	8072	-0.1193
S28 3113EC	13	q	61897207	115108397	36054	-0.1802
S28 3113EC	14	q	20425911	106008645	54802	-0.1516

S28 3113EC	15	q	22752520	102469040	52124	-0.3369
S28 3113EC	16	p	60777	31960151	20324	0.135
S28 3113EC	16	q	46463782	78403146	21286	0.1508
S28 3113EC	16	q	78403585	90287535	10757	0.0862
S28 3113EC	17	p	526	18319862	11622	0.1423
S28 3113EC	17	p	18355392	18465221	30	-0.7319
S28 3113EC	17	p	18469197	22235650	1585	0.1682
S28 3113EC	17	q	25270411	72273204	28060	0.1566
S28 3113EC	17	q	72274155	81048659	4542	0.0964
S28 3113EC	18	p	11543	15402408	10584	-0.1608
S28 3113EC	18	q	18529353	66956043	32667	-0.1589
S28 3113EC	18	q	66956704	78015057	8125	-0.2209
S28 3113EC	19	p	90910	19556926	8565	-0.1181
S28 3113EC	19	p	19557861	24596750	2876	-0.0201
S28 3113EC	19	q	27747993	33637264	4122	-0.1679
S28 3113EC	19	q	33638869	59097854	14232	-0.1043
S28 3113EC	20	p	61305	26305579	20211	0.1481
S28 3113EC	20	q	29420352	58694144	20024	0.1381
S28 3113EC	20	q	58694418	62956153	2655	0.075
S28 3113EC	21	q	14345669	27724762	9556	-0.0274
S28 3113EC	21	q	27724918	41529188	10481	0.0266
S28 3113EC	21	q	41529369	48096957	4546	-0.0335
S28 3113EC	22	q	16052528	46050587	20077	-0.131
S28 3113EC	22	q	46057366	51234455	3871	-0.2285
S28 3113EC	X	p	168477	2694240	427	0.458
S28 3113EC	X	p	2703391	22446730	13384	-0.1553

S28 3113EC	X	p	22454537	23193486	635	-0.6726
S28 3113EC	X	p	23196329	45309085	13726	-0.1835
S28 3113EC	X	p	45312536	58561930	5862	-0.1252
S28 3113EC	X	q	61728829	81477034	8904	-0.146
S28 3113EC	X	q	81479804	88435254	4229	-0.2228
S28 3113EC	X	q	88438261	140727884	28979	-0.1465
S28 3113EC	X	q	140727917	146177414	3872	-0.2576
S28 3113EC	X	q	146177489	154963439	5117	-0.1442
S28 3113EC	X	q	154964055	155233846	36	0.4071
S28 3113EC	Y	p	179542	3825719	337	0.7358
S28 3113EC	Y	p	3827532	10076242	2408	0.3859
S28 3113EC	Y	q	13134531	59018259	6034	0.3693
S4 H14 norm	1	p	61735	121482979	74649	-0.0069
S4 H14 norm	1	q	143982530	187533144	25578	-0.0212
S4 H14 norm	1	q	187533238	195602655	5432	-0.0891
S4 H14 norm	1	q	195606174	249224388	37173	-0.0129
S4 H14 norm	2	p	12784	89131067	60431	-0.0216
S4 H14 norm	2	q	95327887	242783396	90340	-0.0249
S4 H14 norm	2	q	242915466	243089456	142	-0.4869
S4 H14 norm	3	p	60345	44301537	32006	-0.0358
S4 H14 norm	3	p	44303124	90502862	29574	-0.0049
S4 H14 norm	3	q	93519478	197896118	64380	-0.025
S4 H14 norm	4	p	68821	49658612	32066	-0.0394
S4 H14 norm	4	q	52685699	191027923	86343	-0.033
S4 H14 norm	5	p	15532	46389273	31096	-0.0383
S4 H14 norm	5	q	49432831	180790320	82883	-0.0199

S4 H14 norm	6	p	149661	58774716	39810	-0.0218
S4 H14 norm	6	q	61886440	95442489	21472	-0.0389
S4 H14 norm	6	q	95442761	95533338	62	-0.7999
S4 H14 norm	6	q	95540519	171051005	49608	-0.0268
S4 H14 norm	7	p	43259	58019983	41049	-0.0222
S4 H14 norm	7	q	61063974	159127004	58281	-0.0141
S4 H14 norm	8	p	31254	24974476	21955	-0.0325
S4 H14 norm	8	p	24974522	24984333	25	-1.3405
S4 H14 norm	8	p	24991104	43824048	11716	-0.0089
S4 H14 norm	8	q	46847534	146298155	62823	-0.0396
S4 H14 norm	9	p	46587	39110886	31539	-0.0246
S4 H14 norm	9	q	71006575	141091394	48558	-0.0099
S4 H14 norm	10	p	72759	39076221	29502	-0.0337
S4 H14 norm	10	q	42433540	106632242	41405	-0.0145
S4 H14 norm	10	q	106632478	114124709	5482	-0.0904
S4 H14 norm	10	q	114124840	135506704	15665	-0.027
S4 H14 norm	11	p	198510	5783923	3319	-0.0507
S4 H14 norm	11	p	5787405	5809139	33	-1.6419
S4 H14 norm	11	p	5809338	51564427	31391	-0.0291
S4 H14 norm	11	q	54701645	134944770	53268	-0.0211
S4 H14 norm	12	p	150442	34854498	23917	-0.0073
S4 H14 norm	12	q	37857751	133778189	61976	-0.0216
S4 H14 norm	13	q	19026949	115108397	65125	-0.0219
S4 H14 norm	14	q	20425911	106008645	54802	-0.0204
S4 H14 norm	15	q	22752520	102469040	52124	-0.0119
S4 H14 norm	16	p	60777	31960151	20324	-0.0196

S4 H14 norm	16	q	46463782	90287535	32043	-0.0196
S4 H14 norm	17	p	526	22235650	13237	0.0025
S4 H14 norm	17	q	25270411	81048659	32602	0.0088
S4 H14 norm	18	p	11543	15402408	10584	-0.021
S4 H14 norm	18	q	18529353	66961867	32675	-0.0171
S4 H14 norm	18	q	66968006	78015057	8117	-0.0736
S4 H14 norm	19	p	90910	24596750	11441	-0.0063
S4 H14 norm	19	q	27747993	59097854	18354	-0.0081
S4 H14 norm	20	p	61305	26305579	20211	-0.016
S4 H14 norm	20	q	29420352	62956153	22679	-0.0237
S4 H14 norm	21	q	14345669	48096957	24583	-0.0336
S4 H14 norm	22	q	16052528	47262729	20787	0.0051
S4 H14 norm	22	q	47263508	51234455	3161	-0.0689
S4 H14 norm	X	p	168477	2693624	426	-0.0084
S4 H14 norm	X	p	2694240	58561930	33608	-0.5574
S4 H14 norm	X	q	61728829	155233846	51137	-0.5522
S4 H14 norm	Y	p	179542	10076242	2745	0.0241
S4 H14 norm	Y	q	13134531	59018259	6034	-0.0044
S5 Shef6 norm	1	p	61735	61992247	36000	0.0089
S5 Shef6 norm	1	p	61994077	65821164	2878	0.0884
S5 Shef6 norm	1	p	65822382	121482979	35771	-0.0044
S5 Shef6 norm	1	q	143982530	152757690	1924	0.0323
S5 Shef6 norm	1	q	152759678	152768700	38	-1.381
S5 Shef6 norm	1	q	152773905	249224388	66221	-0.0082
S5 Shef6 norm	2	p	12784	89131067	60431	-0.0067
S5 Shef6 norm	2	q	95327887	212585065	70775	-0.0034

S5 Shef6 norm	2	q	212585422	242783396	19565	-0.0376
S5 Shef6 norm	2	q	242915466	243089456	142	-0.4934
S5 Shef6 norm	3	p	60345	44303124	32007	-0.0179
S5 Shef6 norm	3	p	44304517	58666294	8055	0.0284
S5 Shef6 norm	3	p	58667380	61439273	2521	-0.0493
S5 Shef6 norm	3	p	61439743	66700816	4307	0.0828
S5 Shef6 norm	3	p	66700959	90502862	14690	-0.0057
S5 Shef6 norm	3	q	93519478	129766327	21805	-0.013
S5 Shef6 norm	3	q	129766586	129806236	53	-0.9595
S5 Shef6 norm	3	q	129806924	197896118	42522	-0.0047
S5 Shef6 norm	4	p	68821	49658612	32066	-0.0201
S5 Shef6 norm	4	q	52685699	157437060	64117	-0.0038
S5 Shef6 norm	4	q	157441782	191027923	22226	-0.0283
S5 Shef6 norm	5	p	15532	46389273	31096	-0.0169
S5 Shef6 norm	5	q	49432831	145210955	58803	-0.0028
S5 Shef6 norm	5	q	145211029	147496483	1593	0.1123
S5 Shef6 norm	5	q	147498652	180790320	22487	-0.019
S5 Shef6 norm	6	p	149661	58774716	39810	-0.0103
S5 Shef6 norm	6	q	61886440	171051005	71142	-0.011
S5 Shef6 norm	7	p	43259	58019983	41049	-0.0101
S5 Shef6 norm	7	q	61063974	159127004	58281	0.0052
S5 Shef6 norm	8	p	31254	14585338	13506	-0.0174
S5 Shef6 norm	8	p	14585521	15037969	398	0.3045
S5 Shef6 norm	8	p	15038569	43824048	19792	-0.0052
S5 Shef6 norm	8	q	46847534	134169627	54721	-0.0143
S5 Shef6 norm	8	q	134169695	146298155	8102	-0.0705

S5 Shef6 norm	9	p	46587	39110886	31539	-0.0041
S5 Shef6 norm	9	q	71006575	141091394	48558	0.0014
S5 Shef6 norm	10	p	72759	19884654	16414	-0.03
S5 Shef6 norm	10	p	19885268	22604694	1842	0.0735
S5 Shef6 norm	10	p	22612275	39076221	11246	-0.0176
S5 Shef6 norm	10	q	42433540	106628998	41397	0.001
S5 Shef6 norm	10	q	106629471	113877813	5300	-0.0662
S5 Shef6 norm	10	q	113878582	135506704	15855	-0.0134
S5 Shef6 norm	11	p	198510	51564427	34743	-0.0107
S5 Shef6 norm	11	q	54701645	134944770	53268	-0.0078
S5 Shef6 norm	12	p	150442	34854498	23917	0.0035
S5 Shef6 norm	12	q	37857751	133778189	61976	-0.0047
S5 Shef6 norm	13	q	19026949	51569961	23026	0.002
S5 Shef6 norm	13	q	51570161	57801201	3478	0.0907
S5 Shef6 norm	13	q	57801654	115108397	38621	-0.0118
S5 Shef6 norm	14	q	20425911	106008645	54802	-0.0044
S5 Shef6 norm	15	q	22752520	102469040	52124	4.00E-04
S5 Shef6 norm	16	p	60777	31960151	20324	-0.0059
S5 Shef6 norm	16	q	46463782	81903315	25148	-7.00E-04
S5 Shef6 norm	16	q	81904575	90287535	6895	-0.0442
S5 Shef6 norm	17	p	526	22235650	13237	-4.00E-04
S5 Shef6 norm	17	q	25270411	81048659	32602	0.0017
S5 Shef6 norm	18	p	11543	15402408	10584	-0.003
S5 Shef6 norm	18	q	18529353	66961867	32675	-0.002
S5 Shef6 norm	18	q	66968006	78015057	8117	-0.0575
S5 Shef6 norm	19	p	90910	24596750	11441	0.0012

S5 Shef6 norm	19	q	27747993	59097854	18354	-0.0047
S5 Shef6 norm	20	p	61305	26305579	20211	-0.0018
S5 Shef6 norm	20	q	29420352	52644868	15095	-0.0012
S5 Shef6 norm	20	q	52647808	52654644	31	-0.6925
S5 Shef6 norm	20	q	52657717	62956153	7553	-0.043
S5 Shef6 norm	21	q	14345669	48096957	24583	-0.0188
S5 Shef6 norm	22	q	16052528	47195178	20736	0.0146
S5 Shef6 norm	22	q	47195274	51234455	3212	-0.074
S5 Shef6 norm	X	p	168477	58561930	34034	0.0084
S5 Shef6 norm	X	q	61728829	90727403	14086	-0.0144
S5 Shef6 norm	X	q	90729580	116298740	13914	0.0577
S5 Shef6 norm	X	q	116299171	127969409	6915	-0.0033
S5 Shef6 norm	X	q	127969596	139917555	6785	0.0713
S5 Shef6 norm	X	q	139917682	155233846	9437	-0.0298
S5 Shef6 norm	Y	p	179542	6101425	834	-1.1635
S5 Shef6 norm	Y	p	6107901	10076242	1911	-1.5876
S5 Shef6 norm	Y	q	13134531	59018259	6034	-1.6221
S6 NCCIT	1	p	61735	10421878	5324	0.3122
S6 NCCIT	1	p	10428090	62046056	30714	0.3886
S6 NCCIT	1	p	62047663	65887508	2885	0.4576
S6 NCCIT	1	p	65888534	121482979	35726	0.3693
S6 NCCIT	1	q	143982530	159616281	6112	0.5157
S6 NCCIT	1	q	159619356	249224388	62071	0.1406
S6 NCCIT	2	p	12784	89131067	60431	0.1496
S6 NCCIT	2	q	95327887	243089456	90482	0.1355
S6 NCCIT	3	p	60345	1955081	1762	0.3168

S6 NCCIT	3	p	1955132	76898393	52319	-0.0814
S6 NCCIT	3	p	76901035	77283872	234	0.4277
S6 NCCIT	3	p	77285530	80549595	1901	0.1558
S6 NCCIT	3	p	80551182	83068067	1430	0.2626
S6 NCCIT	3	p	83071349	90502862	3934	0.1241
S6 NCCIT	3	q	93519478	94113097	264	-0.6031
S6 NCCIT	3	q	94116047	94785401	322	-0.1771
S6 NCCIT	3	q	94788556	95654115	508	-0.5929
S6 NCCIT	3	q	95659595	96167707	277	0.0113
S6 NCCIT	3	q	96182184	176402304	49489	-0.1855
S6 NCCIT	3	q	176404198	182727097	3745	0.783
S6 NCCIT	3	q	182730041	186248190	2108	0.5479
S6 NCCIT	3	q	186249731	197896118	7667	0.3446
S6 NCCIT	4	p	68821	32052675	21381	-0.1595
S6 NCCIT	4	p	32054591	49658612	10685	-0.2044
S6 NCCIT	4	q	52685699	93276485	25343	-0.1715
S6 NCCIT	4	q	93280082	93530262	136	-0.6076
S6 NCCIT	4	q	93531361	191027923	60864	-0.1816
S6 NCCIT	5	p	15532	36034665	25194	0.1354
S6 NCCIT	5	p	36036719	36494393	343	0.3785
S6 NCCIT	5	p	36494477	46389273	5559	0.1178
S6 NCCIT	5	q	49432831	146690369	59819	0.1478
S6 NCCIT	5	q	146690479	147496276	575	0.4822
S6 NCCIT	5	q	147496402	180790320	22489	0.1487
S6 NCCIT	6	p	149661	15441910	12225	0.18
S6 NCCIT	6	p	15442646	58774716	27585	-0.1589

S6 NCCIT	6	q	61886440	63425302	794	0.0837
S6 NCCIT	6	q	63426649	68486914	3277	-0.2675
S6 NCCIT	6	q	68488461	73653870	3385	-0.5667
S6 NCCIT	6	q	73654658	171051005	63686	-0.1582
S6 NCCIT	7	p	43259	51202250	36561	0.0961
S6 NCCIT	7	p	51203019	51598709	232	0.3642
S6 NCCIT	7	p	51605045	58019983	4256	-0.1748
S6 NCCIT	7	q	61063974	61888128	57	-0.168
S6 NCCIT	7	q	61987173	63321290	359	0.3762
S6 NCCIT	7	q	63322036	69589422	3298	0.757
S6 NCCIT	7	q	69589590	70152637	366	0.4446
S6 NCCIT	7	q	70153068	153869471	50533	0.0765
S6 NCCIT	7	q	153876160	159127004	3668	-0.177
S6 NCCIT	8	p	31254	14817641	13691	-0.2413
S6 NCCIT	8	p	14818788	43824048	20005	-0.1562
S6 NCCIT	8	q	46847534	61556425	8999	-0.1783
S6 NCCIT	8	q	61558678	61677526	83	-0.7337
S6 NCCIT	8	q	61678330	95942244	20486	-0.1776
S6 NCCIT	8	q	95942792	96128512	117	0.2417
S6 NCCIT	8	q	96137852	146298155	33138	-0.1793
S6 NCCIT	9	p	46587	4439526	4644	-0.6877
S6 NCCIT	9	p	4442275	39110886	26895	0.0428
S6 NCCIT	9	q	71006575	141091394	48558	-0.1433
S6 NCCIT	10	p	72759	39076221	29502	-0.1098
S6 NCCIT	10	q	42433540	135506704	62552	0.0974
S6 NCCIT	11	p	198510	10227059	6427	-0.1299

S6 NCCIT	11	p	10227393	10542861	247	0.1845
S6 NCCIT	11	p	10546286	35016611	18073	-0.1401
S6 NCCIT	11	p	35019789	51564427	9996	0.1264
S6 NCCIT	11	q	54701645	134944770	53268	0.1427
S6 NCCIT	12	p	150442	8006506	5437	0.3515
S6 NCCIT	12	p	8007799	8134717	61	0.869
S6 NCCIT	12	p	8135092	18127924	6652	0.3261
S6 NCCIT	12	p	18128339	34854498	11767	0.1642
S6 NCCIT	12	q	37857751	133778189	61976	-0.1446
S6 NCCIT	13	q	19026949	115108397	65125	-0.168
S6 NCCIT	14	q	20425911	76129287	35158	-0.1599
S6 NCCIT	14	q	76135546	77814142	1158	-0.6385
S6 NCCIT	14	q	77835912	106008645	18486	-0.1512
S6 NCCIT	15	q	22752520	56813406	21342	0.2338
S6 NCCIT	15	q	56820911	60718265	2784	0.424
S6 NCCIT	15	q	60718525	75775724	9558	0.2538
S6 NCCIT	15	q	75780257	79266660	1780	0.4075
S6 NCCIT	15	q	79268211	99222682	14242	0.2193
S6 NCCIT	15	q	99227224	102469040	2418	-0.0537
S6 NCCIT	16	p	60777	21588788	14689	-0.1466
S6 NCCIT	16	p	21591340	21827798	118	-0.6185
S6 NCCIT	16	p	21839340	29422409	4644	-0.1734
S6 NCCIT	16	p	29422513	30191907	220	-0.5996
S6 NCCIT	16	p	30226943	31960151	653	-0.1483
S6 NCCIT	16	q	46463782	70839157	15701	-0.1466
S6 NCCIT	16	q	70839337	71202489	241	0.1926

S6 NCCIT	16	q	71202797	90287535	16101	-0.1426
S6 NCCIT	17	p	526	22235650	13237	0.1323
S6 NCCIT	17	q	25270411	28094761	1363	0.0785
S6 NCCIT	17	q	28096498	36456523	5112	-0.0166
S6 NCCIT	17	q	36466027	37082081	313	0.2598
S6 NCCIT	17	q	37084620	59867439	13218	-0.0117
S6 NCCIT	17	q	59869311	81048659	12596	-0.0596
S6 NCCIT	18	p	11543	15402408	10584	-0.1578
S6 NCCIT	18	q	18529353	78015057	40792	-0.1674
S6 NCCIT	19	p	90910	24596750	11441	-0.1436
S6 NCCIT	19	q	27747993	41445949	8572	-0.153
S6 NCCIT	19	q	41446569	41508020	50	-0.7616
S6 NCCIT	19	q	41508080	59097854	9732	-0.1511
S6 NCCIT	20	p	61305	14086211	10797	-0.0897
S6 NCCIT	20	p	14088188	14455497	183	-0.4676
S6 NCCIT	20	p	14459573	26305579	9231	-0.0943
S6 NCCIT	20	q	29420352	62956153	22679	-0.0889
S6 NCCIT	21	q	14345669	48096957	24583	0.1959
S6 NCCIT	22	q	16052528	16506460	98	-0.385
S6 NCCIT	22	q	16553466	51234455	23850	-0.0661
S6 NCCIT	X	p	168477	15773943	10103	-0.5554
S6 NCCIT	X	p	15775884	16240684	267	-0.0791
S6 NCCIT	X	p	16240783	36565375	12895	-0.5542
S6 NCCIT	X	p	36567824	36607451	33	-1.4254
S6 NCCIT	X	p	36609787	44772981	4609	-0.5579
S6 NCCIT	X	p	44773272	44887027	55	-1.2948

S6 NCCIT	X	p	44887450	45320287	216	0.1441
S6 NCCIT	X	p	45330249	52682094	3320	-0.1417
S6 NCCIT	X	p	52683372	58561930	2536	0.343
S6 NCCIT	X	q	61728829	124454416	32958	0.1546
S6 NCCIT	X	q	124459168	133517508	4935	-0.077
S6 NCCIT	X	q	133524940	155233846	13244	0.1415
S6 NCCIT	Y	p	179542	6101425	834	-1.1211
S6 NCCIT	Y	p	6107901	10076242	1911	-1.5637
S6 NCCIT	Y	q	13134531	59018259	6034	-1.5851
S7 NT2	1	p	61735	5873402	2733	-0.2687
S7 NT2	1	p	5879132	6486039	328	-0.7596
S7 NT2	1	p	6489345	8008071	974	-0.2302
S7 NT2	1	p	8008968	24229998	9513	0.0469
S7 NT2	1	p	24232747	32955870	4501	0.4921
S7 NT2	1	p	32955925	85805613	33996	0.3872
S7 NT2	1	p	85808151	110138600	15307	0.1274
S7 NT2	1	p	110139417	110778745	418	0.381
S7 NT2	1	p	110779084	114283963	2601	0.1286
S7 NT2	1	p	114284132	121482979	4278	0.3826
S7 NT2	1	q	143982530	190384638	27530	0.3689
S7 NT2	1	q	190389223	192282838	1148	0.2474
S7 NT2	1	q	192283229	195815418	2479	0.1087
S7 NT2	1	q	195815564	214473794	12568	0.3752
S7 NT2	1	q	214476561	214813487	307	0.6022
S7 NT2	1	q	214813716	218122560	2429	0.3866
S7 NT2	1	q	218123345	218460644	256	0.126

S7 NT2	1	q	218461034	248736075	21284	0.372
S7 NT2	1	q	248737447	248810646	40	-0.5746
S7 NT2	1	q	248814080	249224388	142	0.3615
S7 NT2	2	p	12784	89131067	60431	0.1221
S7 NT2	2	q	95327887	239488802	88462	0.117
S7 NT2	2	q	239490253	243089456	2020	0.0371
S7 NT2	3	p	60345	1422437	1206	0.0174
S7 NT2	3	p	1424718	61176361	41216	0.1239
S7 NT2	3	p	61176788	66797568	4555	0.1881
S7 NT2	3	p	66799019	90502862	14603	0.1148
S7 NT2	3	q	93519478	129762859	21803	0.1075
S7 NT2	3	q	129763698	129806236	55	-0.6494
S7 NT2	3	q	129806924	197896118	42522	0.124
S7 NT2	4	p	68821	31901050	21294	0.1086
S7 NT2	4	p	31901580	38946053	4659	-0.2808
S7 NT2	4	p	38946852	49658612	6113	-0.2277
S7 NT2	4	q	52685699	191027923	86343	-0.2251
S7 NT2	5	p	15532	16596501	12965	-0.2405
S7 NT2	5	p	16597174	16793151	133	0.1393
S7 NT2	5	p	16794133	46389273	17998	-0.2223
S7 NT2	5	q	49432831	180790320	82883	-0.2143
S7 NT2	6	p	149661	58774716	39810	-0.2181
S7 NT2	6	q	61886440	171051005	71142	-0.2239
S7 NT2	7	p	43259	10674898	6615	0.3328
S7 NT2	7	p	10675914	45225102	25584	0.3776
S7 NT2	7	p	45225708	58019983	8850	0.3332

S7 NT2	7	q	61063974	67570488	2420	0.3459
S7 NT2	7	q	67571889	88538493	12104	0.1377
S7 NT2	7	q	88538949	89524849	617	-0.2439
S7 NT2	7	q	89532079	115106676	15221	0.1418
S7 NT2	7	q	115109554	141763124	16741	0.3833
S7 NT2	7	q	141769643	141792107	42	-0.4597
S7 NT2	7	q	141795281	159127004	11136	0.3536
S7 NT2	8	p	31254	16340013	15046	0.1073
S7 NT2	8	p	16341028	16452703	107	-0.1842
S7 NT2	8	p	16456761	43824048	18543	0.1353
S7 NT2	8	q	46847534	67344005	12493	0.0877
S7 NT2	8	q	67344195	71104541	2328	0.162
S7 NT2	8	q	71105892	79253946	5123	-0.1963
S7 NT2	8	q	79257562	92867120	7738	-0.7812
S7 NT2	8	q	92871381	128824096	23054	-0.2207
S7 NT2	8	q	128828698	129013871	124	0.2121
S7 NT2	8	q	129013985	135567046	5156	-0.2368
S7 NT2	8	q	135567804	146298155	6807	-0.2883
S7 NT2	9	p	46587	39110886	31539	0.1229
S7 NT2	9	q	71006575	90885502	14594	0.1419
S7 NT2	9	q	90885659	91263040	205	0.4345
S7 NT2	9	q	91263519	136445712	31533	0.1413
S7 NT2	9	q	136445749	141091394	2226	0.0596
S7 NT2	10	p	72759	20004996	16520	-0.2397
S7 NT2	10	p	20005918	22738382	1812	-0.1461
S7 NT2	10	p	22738816	30548293	5720	0.1233

S7 NT2	10	p	30558712	35412384	3335	-0.1941
S7 NT2	10	p	35419875	35485441	35	-0.83
S7 NT2	10	p	35492937	39076221	2080	-0.234
S7 NT2	10	q	42433540	58512855	9542	-0.2267
S7 NT2	10	q	58516375	58526837	41	-1.6167
S7 NT2	10	q	58532752	106632242	31822	-0.2112
S7 NT2	10	q	106632478	135506704	21147	-0.2398
S7 NT2	11	p	198510	2793865	1034	0.0235
S7 NT2	11	p	2796519	8238500	4154	0.1259
S7 NT2	11	p	8242629	9280171	707	-0.1988
S7 NT2	11	p	9280462	9823804	286	0.1233
S7 NT2	11	p	9827021	36002308	19425	-0.2224
S7 NT2	11	p	36003851	37449395	1140	0.1174
S7 NT2	11	p	37459661	46732143	6167	0.3536
S7 NT2	11	p	46739146	51564427	1830	0.1021
S7 NT2	11	q	54701645	66251369	6178	0.1126
S7 NT2	11	q	66251796	67269125	357	0.385
S7 NT2	11	q	67273835	68241863	378	0.1275
S7 NT2	11	q	68246555	134944770	46355	-0.2206
S7 NT2	12	p	150442	865970	427	0.6685
S7 NT2	12	p	869109	874029	18	-0.2102
S7 NT2	12	p	875354	34854498	23472	0.8169
S7 NT2	12	q	37857751	133778189	61976	0.1248
S7 NT2	13	q	19026949	27627133	6082	-0.1976
S7 NT2	13	q	27630571	67399367	26278	0.1228
S7 NT2	13	q	67402339	72571957	3305	-0.7588

S7 NT2	13	q	72575295	114295647	29228	-0.2292
S7 NT2	13	q	114297034	115108397	232	0.0311
S7 NT2	14	q	20425911	77243667	35908	-0.2064
S7 NT2	14	q	77248479	77488547	160	0.1404
S7 NT2	14	q	77488920	106008645	18734	-0.2298
S7 NT2	15	q	22752520	57068546	21452	-0.2045
S7 NT2	15	q	57070095	102469040	30672	0.1252
S7 NT2	16	p	60777	31960151	20324	0.1118
S7 NT2	16	q	46463782	85428915	28852	0.1146
S7 NT2	16	q	85428947	90287535	3191	0.0515
S7 NT2	17	p	526	22235650	13237	-0.2096
S7 NT2	17	q	25270411	81048659	32602	-0.2126
S7 NT2	18	p	11543	15402408	10584	-0.2113
S7 NT2	18	q	18529353	22471384	2287	-0.1784
S7 NT2	18	q	22475636	27573792	3244	-0.7551
S7 NT2	18	q	27575411	46723233	12763	-0.2125
S7 NT2	18	q	46726435	46854802	61	-0.8082
S7 NT2	18	q	46857487	78015057	22437	-0.2398
S7 NT2	19	p	90910	19245511	8430	0.0914
S7 NT2	19	p	19245771	24596750	3011	0.1811
S7 NT2	19	q	27747993	29899558	1441	0.1051
S7 NT2	19	q	29899675	59097854	16913	-0.2066
S7 NT2	20	p	61305	11561247	8968	0.1206
S7 NT2	20	p	11561471	26305579	11243	-0.2283
S7 NT2	20	q	29420352	32199198	1306	-0.205
S7 NT2	20	q	32199872	62956153	21373	0.1049

S7	NT2	21	q	14345669	42622657	21024	0.3507
S7	NT2	21	q	42623331	48096957	3559	0.2995
S7	NT2	22	q	16052528	47195178	20736	-0.213
S7	NT2	22	q	47195274	51234455	3212	-0.289
S7	NT2	X	p	168477	2694240	427	0.1126
S7	NT2	X	p	2703391	25851564	15711	-0.1815
S7	NT2	X	p	25852089	38182902	8000	-0.2346
S7	NT2	X	p	38184578	53354043	7582	-0.1668
S7	NT2	X	p	53356021	53798275	169	0.1929
S7	NT2	X	p	53803748	57349621	1711	-0.16
S7	NT2	X	p	57355652	57524579	86	-0.7039
S7	NT2	X	p	57526389	58561930	348	-0.1885
S7	NT2	X	q	61728829	121102370	30862	-0.1795
S7	NT2	X	q	121111625	154929498	20238	-0.7364
S7	NT2	X	q	154963439	155233846	37	-0.2261
S7	NT2	Y	p	179542	10076242	2745	-0.2028
S7	NT2	Y	q	13134531	59018259	6034	-0.2363
S8	TERA1	1	p	61735	86341	16	-0.7188
S8	TERA1	1	p	98588	5611729	2483	0.0675
S8	TERA1	1	p	5612956	24239078	11056	-0.2278
S8	TERA1	1	p	24241045	25583341	805	0.1213
S8	TERA1	1	p	25593128	25646986	29	-0.8048
S8	TERA1	1	p	25661501	28864435	1369	0.1286
S8	TERA1	1	p	28866143	54962365	14788	0.3731
S8	TERA1	1	p	54962448	62210714	5630	0.1161
S8	TERA1	1	p	62212604	63810383	1127	0.3834

S8 TERA1	1	p	63812269	66239685	1864	0.2034
S8 TERA1	1	p	66239699	89600145	15482	0.0934
S8 TERA1	1	p	89600387	121482979	20000	-0.2345
S8 TERA1	1	q	143982530	152761923	1926	0.3248
S8 TERA1	1	q	152761939	152768700	36	-0.7966
S8 TERA1	1	q	152773905	174797226	15012	0.3138
S8 TERA1	1	q	174797315	182663035	5445	0.2334
S8 TERA1	1	q	182664810	183405542	472	0.5295
S8 TERA1	1	q	183405664	216636950	22375	0.2396
S8 TERA1	1	q	216637487	216950504	277	-0.0242
S8 TERA1	1	q	216951064	234956342	12517	0.2398
S8 TERA1	1	q	234957051	246358308	8469	0.4114
S8 TERA1	1	q	246364256	246382970	21	-0.4114
S8 TERA1	1	q	246384914	249224388	1633	0.4681
S8 TERA1	2	p	12784	2644343	1807	-0.1954
S8 TERA1	2	p	2644566	5465706	2197	0.0621
S8 TERA1	2	p	5466323	15663754	7875	-0.018
S8 TERA1	2	p	15664325	15886840	165	0.3246
S8 TERA1	2	p	15887323	35243687	12647	0.0036
S8 TERA1	2	p	35243701	41187626	4464	-0.1966
S8 TERA1	2	p	41188587	42953345	1386	-0.0027
S8 TERA1	2	p	42954494	50861450	5664	0.111
S8 TERA1	2	p	50863213	51065562	154	-0.2613
S8 TERA1	2	p	51066831	65657041	9450	0.1
S8 TERA1	2	p	65667126	89131067	14622	-0.2161
S8 TERA1	2	q	95327887	97898583	892	-0.0338

S8 TERA1	2	q	97898653	232196597	82759	-0.2415
S8 TERA1	2	q	232196986	232584807	182	0.1192
S8 TERA1	2	q	232596620	243089456	6649	-0.2664
S8 TERA1	3	p	60345	12885018	10813	0.1714
S8 TERA1	3	p	12885606	36904783	16490	0.1025
S8 TERA1	3	p	36905901	76649542	26591	-0.0408
S8 TERA1	3	p	76652914	77056294	303	-0.5483
S8 TERA1	3	p	77059840	90502862	7383	-0.067
S8 TERA1	3	q	93519478	111257138	10327	-0.0875
S8 TERA1	3	q	111257405	116481414	3366	-0.0204
S8 TERA1	3	q	116482965	116776817	244	-0.232
S8 TERA1	3	q	116779448	116852872	80	-0.7571
S8 TERA1	3	q	116853143	129762859	7786	-0.0612
S8 TERA1	3	q	129763698	129798735	49	-0.7236
S8 TERA1	3	q	129798781	138013197	5091	-0.056
S8 TERA1	3	q	138014972	180584571	26498	0.0963
S8 TERA1	3	q	180584746	189045853	5409	0.1705
S8 TERA1	3	q	189048167	194362721	3764	0.0938
S8 TERA1	3	q	194364005	197896118	1766	-0.0033
S8 TERA1	4	p	68821	533399	206	0.3403
S8 TERA1	4	p	540025	31158655	20598	0.0989
S8 TERA1	4	p	31158917	49658612	11262	0.0638
S8 TERA1	4	q	52685699	77293958	15293	0.0907
S8 TERA1	4	q	77294546	78243186	759	0.2561
S8 TERA1	4	q	78246967	91428124	8227	0.0999
S8 TERA1	4	q	91433714	93198974	1025	-0.2252

S8 TERA1	4	q	93200038	93566850	194	-0.7347
S8 TERA1	4	q	93569306	120263064	16177	-0.2484
S8 TERA1	4	q	120265259	122013042	1136	0.1071
S8 TERA1	4	q	122013475	189395694	42478	-0.2606
S8 TERA1	4	q	189396075	191027923	1054	-0.3941
S8 TERA1	5	p	15532	46389273	31096	0.0604
S8 TERA1	5	q	49432831	57980139	5594	-0.0584
S8 TERA1	5	q	57980670	72150141	7810	0.09
S8 TERA1	5	q	72152374	83979884	7605	0.1377
S8 TERA1	5	q	83982445	87211315	1754	-0.0082
S8 TERA1	5	q	87215161	145083686	35976	0.1029
S8 TERA1	5	q	145086940	147857150	1869	0.1853
S8 TERA1	5	q	147858171	150565638	1882	0.3685
S8 TERA1	5	q	150565724	180790320	20393	0.0979
S8 TERA1	6	p	149661	7919724	6594	0.0911
S8 TERA1	6	p	7922104	8207365	217	-0.3065
S8 TERA1	6	p	8210052	10418570	1605	0.0965
S8 TERA1	6	p	10421807	10692521	233	0.4165
S8 TERA1	6	p	10693124	51193913	26531	0.108
S8 TERA1	6	p	51193953	56813186	3822	-0.2468
S8 TERA1	6	p	56822328	58774716	808	0.0968
S8 TERA1	6	q	61886440	78273405	10404	0.0791
S8 TERA1	6	q	78273521	88018462	6067	-0.2403
S8 TERA1	6	q	88023479	88072864	39	-0.9284
S8 TERA1	6	q	88074204	98552535	6706	-0.2203
S8 TERA1	6	q	98557364	100346319	1245	0.096

S8 TERA1	6	q	100347254	107485668	4446	-0.229
S8 TERA1	6	q	107492252	136345796	18290	0.1069
S8 TERA1	6	q	136349307	139576499	2249	-0.272
S8 TERA1	6	q	139576736	161893625	14940	-0.0604
S8 TERA1	6	q	161894229	171051005	6756	-0.8173
S8 TERA1	7	p	43259	2349608	769	0.166
S8 TERA1	7	p	2358638	43932337	30793	0.3098
S8 TERA1	7	p	43932714	58019983	9487	0.4755
S8 TERA1	7	q	61063974	89572966	15179	0.5118
S8 TERA1	7	q	89576659	91206666	1179	0.6285
S8 TERA1	7	q	91206783	111287998	11846	0.2341
S8 TERA1	7	q	111288089	131198528	11822	-0.0475
S8 TERA1	7	q	131199903	131613277	327	0.2124
S8 TERA1	7	q	131613412	133785177	1480	-0.0289
S8 TERA1	7	q	133785195	133795634	26	-1.0317
S8 TERA1	7	q	133795665	152602044	11807	-0.0477
S8 TERA1	7	q	152602163	157801400	3795	-0.2653
S8 TERA1	7	q	157801764	159127004	820	0.0511
S8 TERA1	8	p	31254	12234400	11310	0.1295
S8 TERA1	8	p	12239835	12412578	81	0.5811
S8 TERA1	8	p	12412802	24974476	10564	0.1575
S8 TERA1	8	p	24974522	24984333	25	-1.1479
S8 TERA1	8	p	24991104	37491554	8275	0.1434
S8 TERA1	8	p	37492668	43824048	3441	0.2614
S8 TERA1	8	q	46847534	95083101	29027	0.3265
S8 TERA1	8	q	95083587	134097522	25608	0.2076

S8 TERA1	8	q	134098665	141086152	5868	0.1284
S8 TERA1	8	q	141086326	142404619	742	0.3783
S8 TERA1	8	q	142410823	146298155	1578	0.2282
S8 TERA1	9	p	46587	34915618	29009	-0.2228
S8 TERA1	9	p	34915735	35227541	165	0.1207
S8 TERA1	9	p	35227684	39110886	2365	-0.2241
S8 TERA1	9	q	71006575	123835199	38747	-0.228
S8 TERA1	9	q	123836653	124650611	465	-0.0048
S8 TERA1	9	q	124651204	133403955	5021	-0.2209
S8 TERA1	9	q	133404894	133471270	32	-0.7831
S8 TERA1	9	q	133471731	134336602	636	0.0816
S8 TERA1	9	q	134337587	140842780	3481	-0.2537
S8 TERA1	9	q	140846158	140993462	107	-0.7877
S8 TERA1	9	q	140994213	141091394	69	-0.1964
S8 TERA1	10	p	72759	39076221	29502	-0.2417
S8 TERA1	10	q	42433540	93906662	33790	-0.2379
S8 TERA1	10	q	93906791	104167651	6133	-0.1861
S8 TERA1	10	q	104170841	123620313	14076	0.0711
S8 TERA1	10	q	123624305	135506704	8553	0.1324
S8 TERA1	11	p	198510	14101750	9797	-0.2206
S8 TERA1	11	p	14102118	14567538	252	0.0443
S8 TERA1	11	p	14568771	34013886	13807	-0.1967
S8 TERA1	11	p	34013967	46176705	8827	0.114
S8 TERA1	11	p	46177084	51564427	2060	0.2914
S8 TERA1	11	q	54701645	61496272	3967	0.308
S8 TERA1	11	q	61499000	81014652	10693	0.1313

S8 TERA1	11	q	81014707	113670052	22649	-0.2472
S8 TERA1	11	q	113671506	123850550	7412	0.1168
S8 TERA1	11	q	123850803	129947102	4615	0.1839
S8 TERA1	11	q	129947739	130485864	347	0.4216
S8 TERA1	11	q	130488342	134944770	3585	0.1069
S8 TERA1	12	p	150442	865736	426	0.834
S8 TERA1	12	p	865970	874029	19	-0.2066
S8 TERA1	12	p	875354	13788475	8967	0.9799
S8 TERA1	12	p	13788498	27188132	9124	1.1899
S8 TERA1	12	p	27192179	32968923	4330	1.0524
S8 TERA1	12	p	32970577	34246785	836	0.8975
S8 TERA1	12	p	34249814	34854498	215	1.2358
S8 TERA1	12	q	37857751	54880191	10003	0.1451
S8 TERA1	12	q	54884972	59258906	2642	0.3249
S8 TERA1	12	q	59259628	83676241	15825	-0.0064
S8 TERA1	12	q	83682307	95666843	7530	-0.2589
S8 TERA1	12	q	95666994	129725576	23275	-0.2166
S8 TERA1	12	q	129726005	130168561	435	0.0141
S8 TERA1	12	q	130177217	133778189	2266	0.1937
S8 TERA1	13	q	19026949	23056291	2539	-0.048
S8 TERA1	13	q	23056553	48550319	18601	0.2276
S8 TERA1	13	q	48553650	115108397	43985	-0.0592
S8 TERA1	14	q	20425911	23018858	1381	0.1248
S8 TERA1	14	q	23019209	50292137	16943	-0.2242
S8 TERA1	14	q	50303593	50645767	206	0.1636
S8 TERA1	14	q	50645974	59050776	5787	-0.2182

S8 TERA1	14	q	59051142	63191927	2682	-0.3626
S8 TERA1	14	q	63194136	80355099	11198	0.037
S8 TERA1	14	q	80355837	106008645	16605	-0.0345
S8 TERA1	15	q	22752520	69119238	29806	-0.2181
S8 TERA1	15	q	69119963	102469040	22318	-0.0223
S8 TERA1	16	p	60777	7954970	5624	-0.0551
S8 TERA1	16	p	7957829	8248660	283	-0.4542
S8 TERA1	16	p	8249274	30687789	13890	-0.0586
S8 TERA1	16	p	30695458	31960151	527	0.2202
S8 TERA1	16	q	46463782	77911738	20795	-0.2326
S8 TERA1	16	q	77912843	78371608	442	0.1161
S8 TERA1	16	q	78372440	78384765	17	-1.0314
S8 TERA1	16	q	78385001	90287535	10789	-0.2635
S8 TERA1	17	p	526	22235650	13237	0.0887
S8 TERA1	17	q	25270411	25667413	201	0.4064
S8 TERA1	17	q	25672660	38530107	7274	0.0363
S8 TERA1	17	q	38536983	38702442	84	0.5219
S8 TERA1	17	q	38707125	41208042	1480	0.0256
S8 TERA1	17	q	41208229	41258761	76	-0.4643
S8 TERA1	17	q	41258791	43208626	977	0.2222
S8 TERA1	17	q	43217165	81048659	22510	0.4288
S8 TERA1	18	p	11543	7111510	5190	0.3528
S8 TERA1	18	p	7120241	7818887	513	0.9242
S8 TERA1	18	p	7821378	9728453	1420	0.6974
S8 TERA1	18	p	9729789	10878261	1009	0.5211
S8 TERA1	18	p	10879214	13704828	1774	0.3189

S8 TERA1	18	p	13705730	15402408	678	-0.2125
S8 TERA1	18	q	18529353	30260012	7350	0.1002
S8 TERA1	18	q	30260489	78015057	33442	-0.2514
S8 TERA1	19	p	90910	8497282	3030	0.0708
S8 TERA1	19	p	8498099	11153126	1324	0.2269
S8 TERA1	19	p	11154129	20124695	4540	0.0804
S8 TERA1	19	p	20124820	22988128	1637	0.2824
S8 TERA1	19	p	22991586	24596750	910	0.1499
S8 TERA1	19	q	27747993	38890858	7191	0.0441
S8 TERA1	19	q	38891509	43228137	2231	-0.331
S8 TERA1	19	q	43228173	49206145	3053	-0.2273
S8 TERA1	19	q	49208052	51772071	1159	0.054
S8 TERA1	19	q	51772194	54157581	1740	0.1689
S8 TERA1	19	q	54159372	57970193	2328	-0.2096
S8 TERA1	19	q	57970811	59097854	652	-0.4764
S8 TERA1	20	p	61305	17849291	14136	-0.3473
S8 TERA1	20	p	17851254	26305579	6075	0.0256
S8 TERA1	20	q	29420352	30023369	164	0.1132
S8 TERA1	20	q	30024180	30287950	125	0.5185
S8 TERA1	20	q	30289824	62956153	22390	0.0863
S8 TERA1	21	q	14345669	19660354	3404	-0.2542
S8 TERA1	21	q	19661872	48096957	21179	0.086
S8 TERA1	22	q	16052528	16491549	82	0.1315
S8 TERA1	22	q	16492713	41506593	16766	-0.2274
S8 TERA1	22	q	41506608	41853729	301	0.1218
S8 TERA1	22	q	41856048	42545221	366	-0.2234

S8	TERA1	22	q	42552875	51234455	6433	-0.4998
S8	TERA1	X	p	168477	9896067	5962	-0.7824
S8	TERA1	X	p	9896441	10112202	165	-0.1966
S8	TERA1	X	p	10112338	11439955	782	-0.7896
S8	TERA1	X	p	11446790	13775368	1859	0.0273
S8	TERA1	X	p	13775930	14388893	439	0.846
S8	TERA1	X	p	14392222	24541884	6161	0.5703
S8	TERA1	X	p	24545160	31475190	4418	0.3266
S8	TERA1	X	p	31475568	32211773	503	0.0095
S8	TERA1	X	p	32211790	32388364	168	0.3992
S8	TERA1	X	p	32388666	32931776	460	0.0913
S8	TERA1	X	p	32932984	42437367	5669	0.3348
S8	TERA1	X	p	42439361	42776949	209	0.6412
S8	TERA1	X	p	42779627	48996681	3167	0.3445
S8	TERA1	X	p	49002780	49150664	108	0.761
S8	TERA1	X	p	49157398	58561930	3964	0.3969
S8	TERA1	X	q	61728829	95428567	16601	-0.2279
S8	TERA1	X	q	95430893	95523547	55	-0.7826
S8	TERA1	X	q	95530690	103006763	4011	-0.2036
S8	TERA1	X	q	103007196	114745067	6406	-0.7341
S8	TERA1	X	q	114745305	155233846	24064	-0.2156
S8	TERA1	Y	p	179542	6101425	834	-1.2429
S8	TERA1	Y	p	6107901	10076242	1911	-1.6004
S8	TERA1	Y	q	13134531	14554683	347	-1.4549
S8	TERA1	Y	q	14559343	59018259	5687	-0.5252
S9	2102Ep	1	p	61735	7984733	4015	0.1171

S9 2102Ep	1	p	7988418	33699363	14360	0.1809
S9 2102Ep	1	p	33699914	121482979	56274	0.2345
S9 2102Ep	1	q	143982530	152757690	1924	0.232
S9 2102Ep	1	q	152759678	152768700	38	-0.5492
S9 2102Ep	1	q	152773905	249224388	66221	0.2095
S9 2102Ep	2	p	12784	34642549	24320	0.2034
S9 2102Ep	2	p	34644109	52540639	13050	0.2358
S9 2102Ep	2	p	52540726	75709560	15033	-0.0935
S9 2102Ep	2	p	75710745	84221705	5648	-0.0278
S9 2102Ep	2	p	84222217	89131067	2380	-0.0949
S9 2102Ep	2	q	95327887	142249331	27977	-0.1093
S9 2102Ep	2	q	142251661	142319071	57	-0.6551
S9 2102Ep	2	q	142333495	212651827	42781	-0.0873
S9 2102Ep	2	q	212652438	239488802	17647	-0.122
S9 2102Ep	2	q	239490253	243089456	2020	-0.1985
S9 2102Ep	3	p	60345	8751601	7791	-0.1528
S9 2102Ep	3	p	8756392	61061697	34521	0.2026
S9 2102Ep	3	p	61061861	61190394	127	-0.1445
S9 2102Ep	3	p	61205838	90502862	19141	0.2211
S9 2102Ep	3	q	93519478	95024768	734	0.2305
S9 2102Ep	3	q	95028650	118123274	14150	-0.104
S9 2102Ep	3	q	118126801	118216344	68	-0.6978
S9 2102Ep	3	q	118216635	192874747	46678	-0.1042
S9 2102Ep	3	q	192877902	192882903	20	0.7626
S9 2102Ep	3	q	192886435	197896118	2730	-0.1436
S9 2102Ep	4	p	68821	49658612	32066	-0.113

S9 2102Ep	4	q	52685699	191027923	86343	-0.0997
S9 2102Ep	5	p	15532	15467656	12174	-0.1378
S9 2102Ep	5	p	15469511	46389273	18922	-0.0966
S9 2102Ep	5	q	49432831	104015009	32320	-0.0993
S9 2102Ep	5	q	104018041	104354166	243	-0.6159
S9 2102Ep	5	q	104355954	118690998	9963	-0.1022
S9 2102Ep	5	q	118698609	171140880	34354	0.2214
S9 2102Ep	5	q	171141491	180790320	6003	0.1747
S9 2102Ep	6	p	149661	32452929	23247	-0.1147
S9 2102Ep	6	p	32454275	32570365	33	0.5817
S9 2102Ep	6	p	32573771	58774716	16530	-0.1081
S9 2102Ep	6	q	61886440	134764428	46076	-0.0856
S9 2102Ep	6	q	134767743	171051005	25066	-0.1244
S9 2102Ep	7	p	43259	2479790	822	0.1031
S9 2102Ep	7	p	2486368	10501031	5676	0.2117
S9 2102Ep	7	p	10501436	14966522	3650	0.3013
S9 2102Ep	7	p	14968163	34945464	15102	0.2459
S9 2102Ep	7	p	34952037	58019983	15799	0.2082
S9 2102Ep	7	q	61063974	81807082	10327	0.2222
S9 2102Ep	7	q	81808615	85360158	2355	0.3267
S9 2102Ep	7	q	85361076	88609298	1910	0.2163
S9 2102Ep	7	q	88609352	138013432	30185	-0.099
S9 2102Ep	7	q	138018587	141763124	2326	0.2403
S9 2102Ep	7	q	141769643	141799117	46	-0.3929
S9 2102Ep	7	q	141799160	142499677	453	0.1886
S9 2102Ep	7	q	142500467	149610781	4429	-0.0987

S9 2102Ep	7	q	149612796	159127004	6250	-0.1545
S9 2102Ep	8	p	31254	30488280	25923	-0.6467
S9 2102Ep	8	p	30495586	37856947	4456	0.2292
S9 2102Ep	8	p	37864455	43824048	3317	-0.1188
S9 2102Ep	8	q	46847534	67974475	12757	-0.1212
S9 2102Ep	8	q	67974782	134021212	41791	-0.0897
S9 2102Ep	8	q	134021388	146298155	8275	-0.1471
S9 2102Ep	9	p	46587	8636004	8419	0.1969
S9 2102Ep	9	p	8639600	39110886	23120	0.2397
S9 2102Ep	9	q	71006575	116485434	32784	-0.0949
S9 2102Ep	9	q	116487457	116537108	46	-0.6529
S9 2102Ep	9	q	116548430	128975777	9078	-0.08
S9 2102Ep	9	q	128975791	141091394	6650	-0.1645
S9 2102Ep	10	p	72759	7926226	6946	-0.1497
S9 2102Ep	10	p	7928278	8047550	76	-0.6738
S9 2102Ep	10	p	8047948	18526451	8305	-0.1186
S9 2102Ep	10	p	18527039	18670717	130	-0.6924
S9 2102Ep	10	p	18671792	39076221	14045	-0.1079
S9 2102Ep	10	q	42433540	47132459	2606	-0.1077
S9 2102Ep	10	q	47133101	47948060	223	0.2233
S9 2102Ep	10	q	47960070	128867850	54941	-0.1031
S9 2102Ep	10	q	128867901	135506704	4782	-0.1568
S9 2102Ep	11	p	198510	3398568	1337	-0.1844
S9 2102Ep	11	p	3398662	51564427	33406	-0.0999
S9 2102Ep	11	q	54701645	55376280	251	-0.1301
S9 2102Ep	11	q	55376498	55442318	42	-1.5051

S9 2102Ep	11	q	55444211	90172749	20404	-0.1043
S9 2102Ep	11	q	90174653	114184192	17013	-0.6374
S9 2102Ep	11	q	114184389	134944770	15558	-0.112
S9 2102Ep	12	p	150442	8556693	5683	1.0406
S9 2102Ep	12	p	8558488	8584672	22	0.2353
S9 2102Ep	12	p	8594894	11503975	1771	1.1027
S9 2102Ep	12	p	11511577	11543406	32	0.0134
S9 2102Ep	12	p	11552192	14694202	2483	1.0508
S9 2102Ep	12	p	14694571	24319028	6294	1.1477
S9 2102Ep	12	p	24319163	27645992	2554	1.068
S9 2102Ep	12	p	27647741	27651362	28	-0.022
S9 2102Ep	12	p	27657161	34854498	5050	1.0906
S9 2102Ep	12	q	37857751	70466545	20012	0.2315
S9 2102Ep	12	q	70467612	89797611	12010	0.2647
S9 2102Ep	12	q	89815873	133778189	29954	0.1979
S9 2102Ep	13	q	19026949	31107818	8679	-0.1179
S9 2102Ep	13	q	31108508	108779455	52212	-0.6176
S9 2102Ep	13	q	108779750	115108397	4234	-0.6693
S9 2102Ep	14	q	20425911	92752904	46290	-0.0901
S9 2102Ep	14	q	92753783	106008645	8512	-0.1485
S9 2102Ep	15	q	22752520	24674887	1009	-0.0811
S9 2102Ep	15	q	24675133	24718173	39	-0.8474
S9 2102Ep	15	q	24718596	76869929	33170	-0.1005
S9 2102Ep	15	q	76878362	76895775	23	-0.8258
S9 2102Ep	15	q	76902179	102469040	17883	-0.1237
S9 2102Ep	16	p	60777	2712141	914	0.0823

S9 2102Ep	16	p	2712155	7026531	3501	0.1956
S9 2102Ep	16	p	7029936	7169430	194	-0.1238
S9 2102Ep	16	p	7170088	31960151	15715	0.1983
S9 2102Ep	16	q	46463782	59884248	9157	0.2048
S9 2102Ep	16	q	59884626	60063094	102	-0.1477
S9 2102Ep	16	q	60063873	74247910	8641	0.2256
S9 2102Ep	16	q	74248789	85428915	10952	-0.13
S9 2102Ep	16	q	85428947	90287535	3191	-0.189
S9 2102Ep	17	p	526	3683214	1992	-0.1515
S9 2102Ep	17	p	3686003	22235650	11245	0.2032
S9 2102Ep	17	q	25270411	39421708	8040	0.2053
S9 2102Ep	17	q	39423014	39430531	24	-0.5972
S9 2102Ep	17	q	39432724	72472079	20091	0.2147
S9 2102Ep	17	q	72474608	81048659	4447	0.1479
S9 2102Ep	18	p	11543	8244335	6006	-0.0988
S9 2102Ep	18	p	8250948	8383646	130	-2.0214
S9 2102Ep	18	p	8387680	15402408	4448	-0.1227
S9 2102Ep	18	q	18529353	72254867	36749	-0.0999
S9 2102Ep	18	q	72256692	78015057	4043	-0.1647
S9 2102Ep	19	p	90910	20038309	8821	0.1661
S9 2102Ep	19	p	20038967	24596750	2620	0.2636
S9 2102Ep	19	q	27747993	43315611	9450	-0.128
S9 2102Ep	19	q	43321137	43740965	209	-0.5604
S9 2102Ep	19	q	43753301	59097854	8695	-0.1078
S9 2102Ep	20	p	61305	10444545	8082	0.44
S9 2102Ep	20	p	10444992	26305579	12129	0.2165

S9 2102Ep	20	q	29420352	60307431	21536	0.1993
S9 2102Ep	20	q	60308845	62956153	1143	0.0925
S9 2102Ep	21	q	14345669	20342268	3915	0.4452
S9 2102Ep	21	q	20343858	34761822	10840	0.2479
S9 2102Ep	21	q	34762384	36309981	1066	0.4301
S9 2102Ep	21	q	36310106	48096957	8762	0.1763
S9 2102Ep	22	q	16052528	46043202	20073	-0.1222
S9 2102Ep	22	q	46044787	51234455	3875	-0.1935
S9 2102Ep	X	p	168477	2694240	427	0.1692
S9 2102Ep	X	p	2703391	58561930	33607	-0.0782
S9 2102Ep	X	q	61728829	155233846	51137	-0.0595
S9 2102Ep	Y	p	179542	10076242	2745	-0.1018
S9 2102Ep	Y	q	13134531	59018259	6034	-0.1389
